# Synthesis, Characterization and Toxicity Evaluation of Some New Heterocyclic Compounds from Oxazole and 1,2,4-Triazine Classes

**DOI:** 10.3390/molecules31101580

**Published:** 2026-05-09

**Authors:** Stefania-Felicia Barbuceanu, Elena-Valentina Rosca, Laura-Ileana Socea, Lavinia Liliana Ruta, Alexandra Carlan, Ileana Cornelia Farcasanu, Constantin Draghici, George Mihai Nitulescu, Elena-Mihaela Pahontu, Rica Boscencu, Octavian Tudorel Olaru, Lucian Iscrulescu, Theodora-Venera Apostol

**Affiliations:** 1 Faculty of Pharmacy, “Carol Davila” University of Medicine and Pharmacy, 6 Traian Vuia Street, 020956 Bucharest, Romania; elenavalentinarosca90@gmail.com (E.-V.R.); george.nitulescu@umfcd.ro (G.M.N.); elena.pahontu@umfcd.ro (E.-M.P.); rica.boscencu@umfcd.ro (R.B.); octavian.olaru@umfcd.ro (O.T.O.); lucian.iscrulescu@umfcd.ro (L.I.); theodora.apostol@umfcd.ro (T.-V.A.); 2 Department of Organic Chemistry, Biochemistry and Catalysis, Faculty of Chemistry, University of Bucharest, 90–92 Sos. Panduri Street, 050663 Bucharest, Romania; lavinia.ruta@chimie.unibuc.ro (L.L.R.); alexandra.carlan@s.unibuc.ro (A.C.); ileana.farcasanu@chimie.unibuc.ro (I.C.F.); 3 “C. D. Nenitescu” Institute of Organic and Supramolecular Chemistry Romanian Academy, 202B Splaiul Independenței, 060023 Bucharest, Romania; cst.drag@yahoo.com

**Keywords:** oxazol-5(4*H*)-one, 1,2,4-triazin-6(5*H*)-one, 4-chlorophenylsulfonylphenyl, arylidene, *Saccharomyces cerevisiae*, *BLH1*, structural similarity analysis, ChEMBL, DrugBank

## Abstract

This study presents the synthesis of novel heterocyclic derivatives from oxazol-5(4*H*)-ones and 1,2,4-triazin-6(5*H*)-ones classes containing the 4-chlorophenylsulfonylphenyl and arylidene motifs as potential bioactive molecules. The synthesis of new oxazol-5(4*H*)-ones was conducted by cyclocondensation of 2-(4-(4-chlorophenylsulfonyl)benzamido)acetic acid with several aromatic aldehydes**.** The reaction of oxazol-5(4*H*)-ones with phenylhydrazine afforded the new 1,2,4-triazin-6(5*H*)-ones. Spectroscopic techniques (IR, ^1^H-, ^13^C-NMR, and MS) and elemental analysis were used to confirm the structures of all new compounds. The compounds were tested against *Saccharomyces cerevisiae*, and cells lacking the *BLH1* gene were more susceptible to compound toxicity. Moreover, the compounds increased bleomycin toxicity against yeast cells. Structural similarity analysis against the ChEMBL database and approved drugs from DrugBank was performed to evaluate the structural novelty of the synthesized compounds and to obtain preliminary information regarding their potential pharmacological profiles.

## 1. Introduction

**Figure 1:**
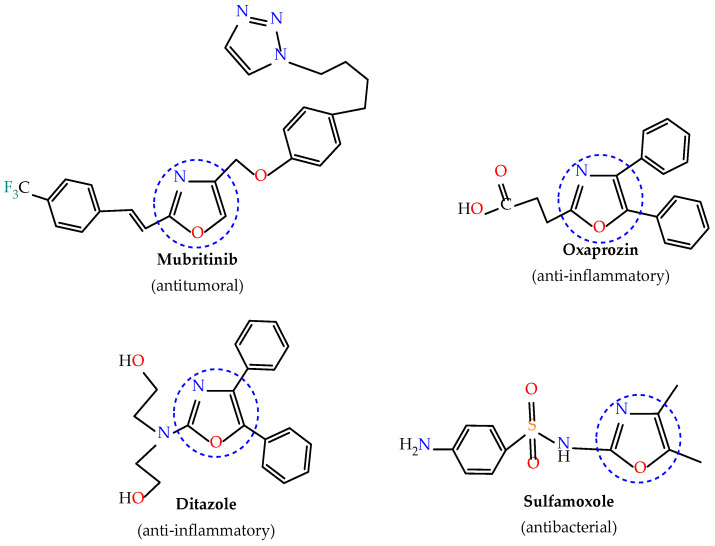
Structures of some oxazole bioactive agents.

The increasing number of people suffering from various diseases as a consequence of modern lifestyles is a major concern worldwide. Allocating additional efforts to intensify research into the discovery of new drugs is on the priority list of governments around the world. Interdisciplinary collaboration between researchers from different fields, including chemists, biochemists, and physicians, offers promising prospects for the development of new drugs, superior to those currently used, to save as many suffering humans as possible. In this context, heterocyclic compounds represent a large family of organic compounds known for their huge potential as new therapeutic agents [1]. Among these, heterocycles containing nitrogen and oxygen heteroatoms exhibit unique chemical and biological properties, and are known for a wide range of applications in various fields, such as the pharmaceutical, agrochemical, and dye industries [2]. This class includes 1,3-oxazoles, whose moiety is a versatile scaffold in the development of drugs with different actions. Mubritinib, Oxaprozin, Ditazole, and Sulfamoxole are some examples of bioactive substances containing the oxazole core (Figure 1) [3,4,5]. Unsaturated oxazol-5(4*H*)-ones (azlactones), which have an exocyclic double bond at position 4, are building blocks for the synthesis of various acyclic compounds (e.g., amides and amino acid derivatives) or heterocycles, including five- and six-membered rings, such as triazinones, imidazolones, and triazoles [6,7,8,9,10,11,12,13,14]. Various unsaturated azlactones are cited in literature for their antibacterial [15,16,17,18], antifungal [15,16], anticancer [15,19,20,21,22], anti-inflammatory [23,24], antioxidant [10,17,18,25], anti-Alzheimer [26], antidiabetic and anti-obesity [27] activities.

**Figure 2:**
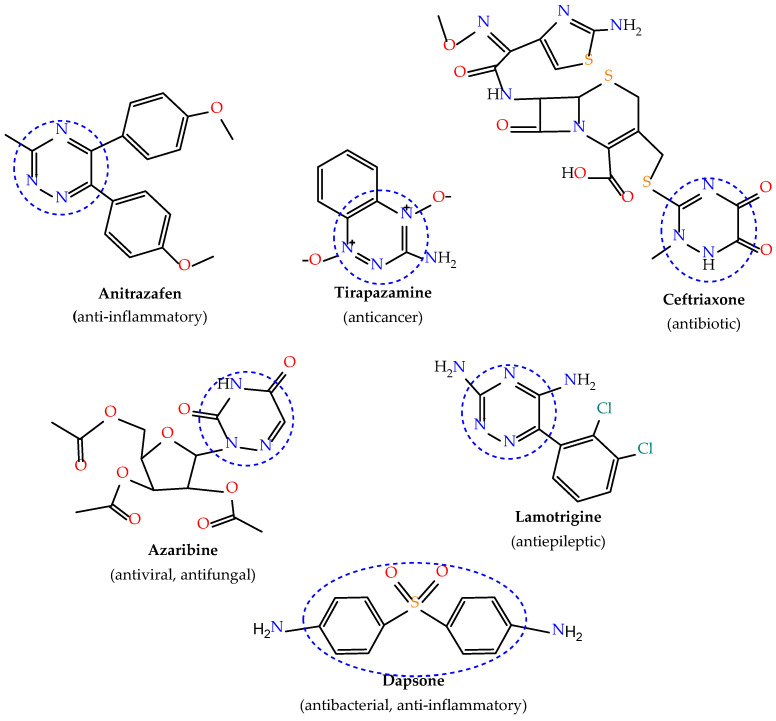
Structures of some 1,2,4-triazine pharmacologically active compounds and of Dapsone drug.

Another important class of heterocycles is that of the 1,2,4-triazines, compounds with a six-membered skeleton containing three nitrogen atoms, which are of particular interest in medicinal chemistry due to their therapeutic effects. Numerous derivatives are bioactive substances, among which are: Anitrazafen, Tirapazamine, Ceftriaxone, Azaribine, and Lamotrigine [28,29,30,31,32,33,34] (Figure 2). Among the above-mentioned compounds that can be obtained from oxazolones, 1,2,4-triazin-6(5*H*)-one derivatives stand out for their biological potential. The 1,2,4-Triazin-6(5*H*)-one ring is a unique pharmacophore associated with antibacterial [9,18,35], antimalarial [35], antifungal [9,35], antioxidant [18], anticancer [14,36,37,38,39], anticonvulsant [40], and anti-inflammatory [24,41] properties.

Other privileged molecules are sulfones, compounds that have gained increasing interest from a chemical point of view, being described as “chemical chameleons”, due to the wide variety of chemical transformations in which the sulfonyl group participates through the electron-withdrawing effect, as well as biologically, because this important motif is embedded in a large number of bioactive agents [41,42,43,44,45]. Various diaryl sulfone derivatives are cited as having antibacterial [46,47,48], antifungal [48], anticancer [49], antiviral [50], antioxidant [48,51], and anti-inflammatory [52] effects, with Dapsone being a well-known drug in this class (Figure 2).

Inspired by the biological value of these molecules and the important role they play in drug discovery, motivated by our continuous efforts in this direction and encouraged by the results of our previous research [53,54,55,56,57], this work aimed to obtain novel heterocyclic compounds with oxazol-5(4*H*)-one or 1,2,4-triazin-6(5*H*)-one nuclei containing the 4-chlorophenylsulfonylphenyl and arylidene fragments, with potential biological activity. The obtained derivatives were tested for toxicity against *Saccharomyces cerevisiae* cells. Comparison with compounds from the ChEMBL database and with approved drugs from DrugBank allows positioning of the new derivatives within the known chemical space and may suggest potential pharmacological properties based on structural resemblance to previously reported molecules.

## 2. Results

### 2.1. Chemistry

**Scheme 1:**
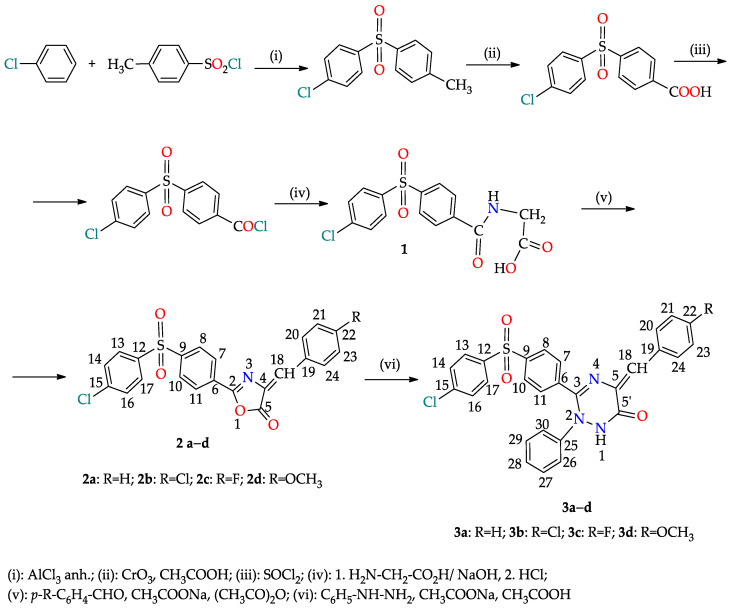
Synthesis of new oxazol-5(4*H*)-ones **2a**–**d** and 1,2,4-triazin-6(5*H*)-ones **3a**–**d** derivatives with 4-chlorophenylsulfonylphenyl and arylidene scaffolds.

Guided by the structural features and biological properties of oxazolones, triazinones, and diarylsulfones, new heterocyclic derivatives from these classes were obtained. As shown in Scheme 1, the synthesis of new oxazolones involved the preparation of the hippuric acid derivative **1**, which consisted of four successive steps: the reaction of 4-methylbenzene-1-sulfonyl chloride with chlorobenzene, followed by oxidation of the formed 1-chloro-4-tosylbenzene, then by transformation of the corresponding acid into the acyl chloride and, finally, acylation of the latter product with glycine [58]. Subsequently, Erlenmeyer condensation of the key intermediate, 2-(4-(4-chlorophenylsulfonyl)benzamido)acetic acid **1**, with aromatic aldehydes (i.e., benzaldehyde or 4-chloro-/4-fluoro-/4-methoxybenzaldehide), led to the new 4-(4-R-benzylidene)-2-(4-(4-chlorophenylsulfonyl)phenyl)oxazol-5(4*H*)-ones **2a**–**d**. The synthesis of new 5-(4-R-benzylidene)-3-(4-(4-chlorophenylsulfonyl)phenyl)-2-phenyl-1,2-dihydro-1,2,4-triazin-6(5*H*)-ones **3a**–**d** was accomplished by refluxing the new oxazolones **2** with phenylhydrazine in glacial acetic acid and in the presence of sodium acetate.

The elemental analyses and ^1^H-NMR, ^13^C-NMR, MS, and IR spectral techniques confirmed the structures of the newly obtained heterocycles. In the IR spectra of the new oxazolones **2a**–**d**, two absorption bands appeared in the 1770–1797 cm^−1^ region, due to the Fermi resonance, corresponding to the stretching vibration of the C=O group [53]. The ^1^H-NMR spectra displayed a new single singlet at 7.41 ppm for **2a** and **2d** or at 7.46 ppm for **2b** and **2c,** characteristic of H-18. The ^13^C-NMR spectra exhibited a signal assigned to C-18 from the aromatic aldehyde fragment, in the range of 131.3–135.2 ppm. The C=O carbon (C-5) from the oxazolone ring was noticed at *δ* between 166.5 and 168.9 ppm. The proton and carbon signals from NMR spectra assigned to the phenyl motif from aldehydes confirmed the cyclocondensation reaction of the hippuric acid derivative **1** with the corresponding aromatic aldehydes. The IR spectra of the new triazinones **3a**–**d** showed two absorption bands in the ranges 3280–3287 cm^−1^ and 1709–1721 cm^−1^ characteristic of the stretching vibrations of the NH and C=O groups, respectively. The ^1^H-NMR spectra highlighted a downfield singlet signal at *δ* 8.98 ppm for **3a**/**3d** and 9.00 ppm for **3b**/**3c** assigned to the NH proton generated from the hydrazine moiety. The H-18 proton was assigned at ≈7.34 ppm as a single singlet signal. The ^13^C-NMR spectra confirmed the structure of the triazinones by the appearance of new signals corresponding to the newly introduced phenylhydrazine motif. The C-18 and C=O (C-5′) signals occurred in the region at δ 128.2–129.6 ppm and 168.6–168.8 ppm, respectively. Another support that confirmed the structure of the new heterocyclic derivatives was the mass spectra, with the chlorine isotopes ^35^Cl/^37^Cl being found in molecular ions or other fragments. The NMR, IR, and mass spectra of the new compounds are included in the Supplementary Materials.

### 2.2. Toxicity Evaluation

#### 2.2.1. Toxicity Screening Against *Saccharomyces cerevisiae*

**Figure 3:**
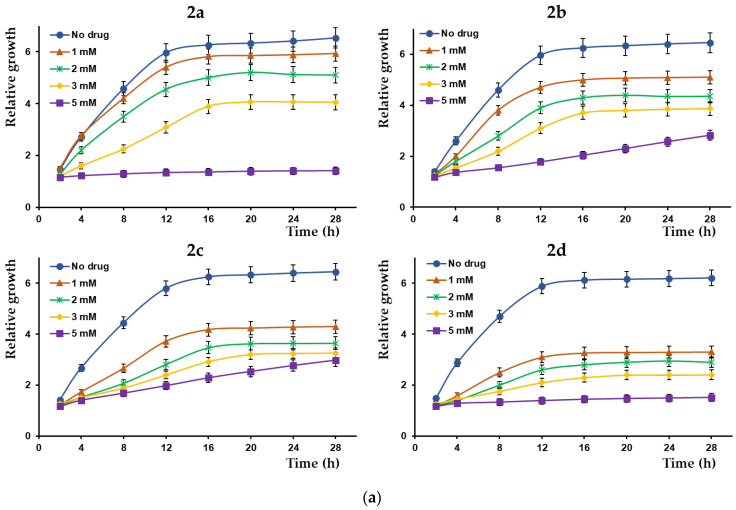

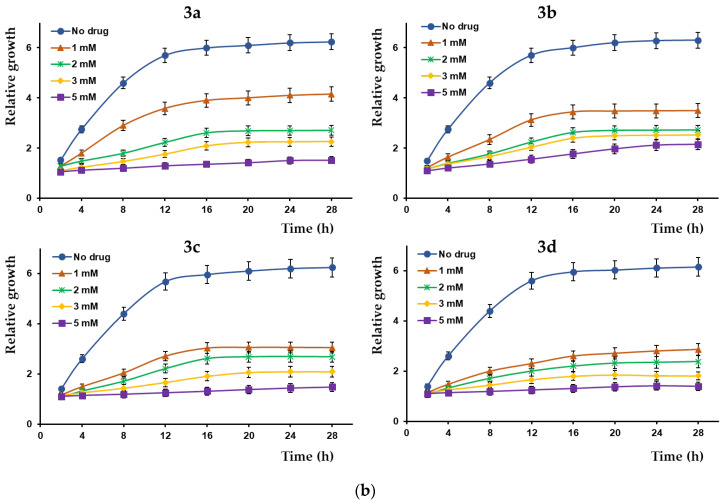
Effect of compounds **2a**–**d** (**a**) and **3a**–**d** (**b**) on the proliferation of *S. cerevisiae* cells. Early log phase BY4741 cells cultured in SC medium were exposed to various concentrations of **2a**–**d** or **3a**–**d** and incubated at 28 °C with shaking (200 rpm). Cell proliferation at various times was calculated as the ratio between OD_600_ measured for cells grown in the presence of each individual compound and the OD_600_ of cells at the time when compounds were added (considered time 0). Values are mean ± SEM of triplicate determinations performed in three biological repeats.

We monitored the eventual toxicity of the compounds **2a**–**d** and **3a**–**d** against *Saccharomyces cerevisiae* cells. As depicted in Figure 3, these compounds had a moderate inhibitory effect on *S. cerevisiae* growth and not in the concentration range that would recommend them as potential antifungals. In fact, compounds **2a**–**d** (Figure 3a) and **3a**–**d** (Figure 3b) have a significant inhibitory effect on cell proliferation only when reaching or even surpassing a 5 mM concentration in the growth medium.

It was noted that in the oxazole series, compound **2d** caused the strongest inhibition of cell proliferation (Figure 3a), while in the triazine series, compounds **3c** and **3d** were more efficient (Figure 3b).

We further calculated the inhibitory concentration of 50% (IC50%) and 90% (IC90%) of yeast cell proliferation after 24 h of cell exposure to drugs. As shown in Table 1, IC50 ranged from 1.25 mM (**2d**) to 7.4 mM (**2a**), observed at a much higher concentration than for a demonstrated antifungal, such as fluconazole (IC50 = 0.006 mM). Similarly, IC90 was higher than 7 mM for all compounds, considerably higher than IC90 for fluconazole (0.53 mM).

#### 2.2.2. Effect on the Growth of *S. cerevisiae* Cells Lacking the Gene for Bleomycin Hydrolase BLH1

**Figure 4:**
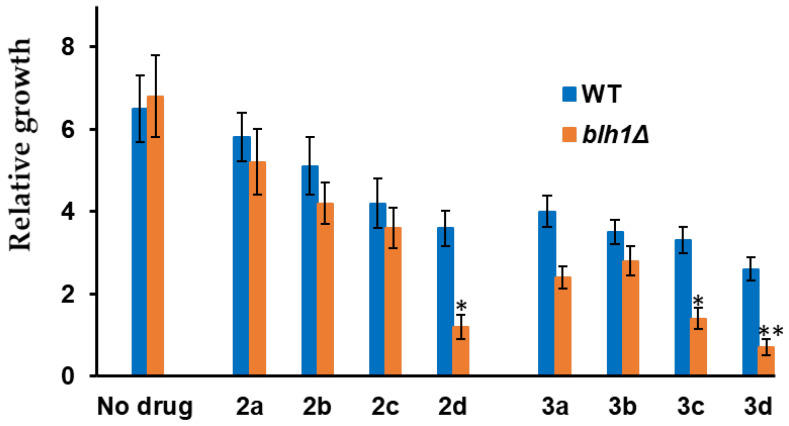
Effect of *BLH1* deletion on the susceptibility of *S. cerevisiae* cells to compounds **2a**–**d** and **3a**–**d**. Early log phase yeast cells (WT and *blh1Δ*) cultured in SC medium were exposed to **2a**–**d** or **3a**–**d** and incubated at 28 °C with shaking (200 rpm). Relative cell growth was determined 16 h after drug addition, as the ratio between OD_600_ measured for cells grown in the presence of each individual compound to the OD_600_ of cells at the time when compounds were added (considered time 0). The concentration of compounds was 1 mM each. Values are mean ± SEM of triplicate determinations completed on three biological repeats. Two-way ANOVA, * *p* < 0.05; ** *p* < 0.01.

Compounds **2a**–**d** and **3a**–**d** are not significantly toxic to yeast cells, but they clearly perturb cell proliferation (Figure 3). To identify potential biological targets of **2a**–**d** or **3a**–**d,** we screened the compounds against a number of yeast mutants lacking individual genes. In the screening experiment, cells were exposed to moderate concentrations (e.g., 1 mM) that would inhibit cell proliferation by less than 50%. We identified *blh1Δ* as being more sensitive than the wild type. *BLH1* encodes for a bleomycin hydrolase and is orthologous to human BLMH, a cysteine-aminopeptidase with bleomycin hydrolase activity in vitro [59,60]. We noticed that yeast cells lacking the *BLH1* gene were more susceptible to **2a**–**d** or **3a**–**d** exposure, especially **2d** and **3d** (Figure 4).

This observation suggests that oxazolone **2d** or triazinones **3c** and **3d** may be the target of Blh1 action through hydrolysis or another mechanism. If that were the case, the absence of Blh1 from yeast cells may allow the compounds to linger inside yeast cells, exerting their toxicity. Further studies are necessary to confirm such a hypothesis.

#### 2.2.3. Effect of Bleomycin Addition on Compounds Toxicity

**Figure 5:**
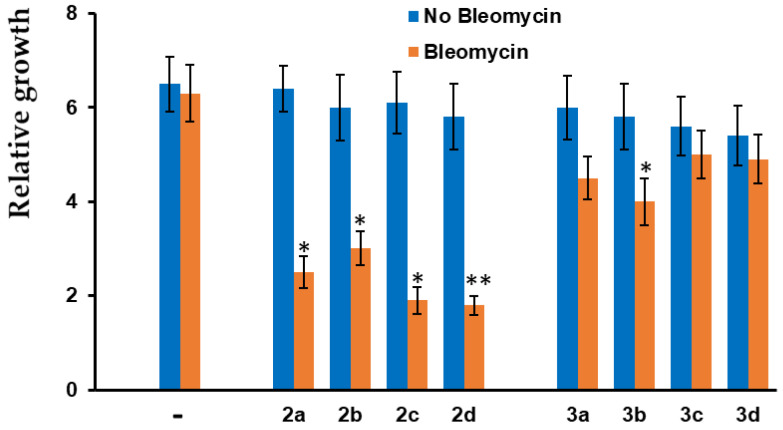
Effect of bleomycin addition on the toxicity of compounds **2a**–**d** and **3a**–**d**. Early log phase BY4741 cells cultured in SC medium were exposed to 0.2 mM **2a**–**d** or **3a**–**d** in the absence or presence of 0.2 mM bleomycin and incubated at 28 °C with shaking (200 rpm). Relative cell growth was determined 16 h after drug addition, as the ratio between OD_600_ measured for cells grown in the presence of each compound and OD_600_ of cells at the time when compounds were added (considered time 0). Values are mean ± SEM of triplicate determinations performed on three biological repeats. Two-way ANOVA, * *p* < 0.05; ** *p* < 0.01.

Bleomycin, an anticancer drug, is highly effective in the treatment of certain cancers when used in combination therapy; therefore, we wondered if a combination of bleomycin and compounds **2a**–**d** or **3a**–**d** would result in alteration of either drug effect. To test this possibility, we exposed the wild-type cells to combinations of bleomycin and each individual compound. For this purpose, a virtually non-toxic concentration was used for each tested compound (i.e., 0.2 mM). It was shown that bleomycin has no significant toxicity against yeast cells [60]. Nevertheless, when used in combination with **2a**–**d** or **3a**–**d**, its toxicity was considerably elevated, especially by compounds **2a**–**d** (Figure 5).

Conversely, the toxicity of compounds **2a**–**d** and **3b** was enhanced by the presence of a non-toxic concentration of bleomycin, indicating a combined action of these compounds with bleomycin.

### 2.3. Structural Similarity to ChEMBL Compounds

The similarity search identified a total of 61 unique ChEMBL compounds exhibiting ≥50% structural similarity to at least one of the eight synthesized compounds. The majority of similarity values clustered within the 50–60% range, indicating moderate structural resemblance to known compounds. A substantial proportion of matches were located near the 50% cutoff, suggesting limited but detectable scaffold similarity.

The highest similarity score was observed between compound **2a** and CHEMBL2003639, reaching approximately 82.5%. Only six compound pairs exceeded a similarity threshold of 70%, indicating that high structural resemblance to known ChEMBL entries is relatively low within the dataset.

Series 2 (**2a**–**2d**) shares significant structural features with previously reported chemotypes and occupies a relatively well-explored region of chemical space within the ChEMBL database. Compounds **2a** and **2b** yielded the highest numbers of analogous compounds (35 and 34 hits, respectively), followed by **2d** (23 hits) and **2c** (17 hits). In contrast, the Series 3 (**3a**–**3d**) exhibited markedly fewer matches. Only 1 up to 4 similar compounds were identified for **3a**, **3b**, and **3d**, while no detectable analogues were found for **3c** at the 50% similarity threshold. This pattern suggests that Series 3 displays a higher degree of structural novelty compared to Series 2.

Consistent with these observations, the Series 2 exhibited a higher overall mean similarity to ChEMBL compounds than the Series 3 (57.12% vs. 54.24%). Although the difference is moderate, it supports the conclusion that Series 2 structures show greater scaffold resemblance to previously reported compounds.

A clear positive association was observed between the average similarity score and the number of target compounds matched. Compounds with high average similarity values (63–70%) were typically similar to four target compounds. Moderately high averages (56–59%) corresponded to similarity with three targets, while intermediate values (~53–55%) were associated with similarity to two targets. Similarity scores close to the 50–52% threshold were predominantly linked to similarity with only a single target compound.

This trend indicates that structurally conserved chemotypes within the dataset tend to exhibit broader cross-compound similarity, whereas more structurally unique compounds display limited overlap with known chemical entities.

### 2.4. Similarity Biological Profile

The data extracted from ChEMBL represent 1727 data points for the 61 structurally similar ChEMBL compounds. Among these, 275 entries reported quantitative values with the standard relation “=”. The most frequent standard type endpoints were inhibition, activity, and IC_50_ values.

The manual analysis of these data revealed several active compounds, as described below.

Compound CHEMBL4753913 emerged as a promising anticancer lead with very high potency against the MCF-7 breast cancer cell line and HCT-116 colon cancer cells, and with medium selectivity versus the normal fibroblast line WI-38. The compound also presented good EGFR T790M and HER2 inhibition and clear apoptosis/cell cycle effects in MCF7 cells. This compound shares a 50% structural similarity with the newly synthesized compound **3d**, indicating a potential anticancer effect for this compound.

Compound CHEMBL4797445 also shows high potency in cancer cells and strong anticonvulsant biomarker effects but appears less selective than CHEMBL4753913 because it is also toxic to WI-38 cells. This compound shares a 52.4% structural similarity with **3d**, in line with the observations for CHEMBL4753913.

Compound CHEMBL4865603 is a very potent cyclooxygenase (COX) inhibitor, especially COX-2 (11 nM), with roughly 9-fold selectivity over COX-1. Its similarity with **3d** (64.1%) and **3b** (51.6%).

Compound CHEMBL2205188 was tested across a large human tumor panel at 10 µM but showed only weak growth inhibition in a few lines, indicating no significant anticancer potential. Compound **2a** shares a 62.2% similarity, while **2b** shows a similarity value of 54.3%, pointing to low anticancer potential for these two molecules. This prediction is confirmed by the low anticancer effect registered for CHEMBL1988306 (similarity 82.5% with **2a**, 73.2% with **2b**) and CHEMBL2003639 (similarity 73.8% with **2a**, 73.2% with **2b**) on the NCI60 cell panel [61].

### 2.5. Similarity Profile with Approved Drugs

The similarity analysis identified 4648 pairs formed between the newly synthesized compounds (Series 2 and Series 3) and approved drugs from DrugBank. In total, 801 unique approved drugs were found among the matched pairs. A total of 202 drugs appear in at least one pair with all compounds from Series 2, and 364 drugs with the compounds from Series 3. The mean similarity values calculated across the six similarity metrics ranged between 0.526 and 0.545.

**Figure 6:**
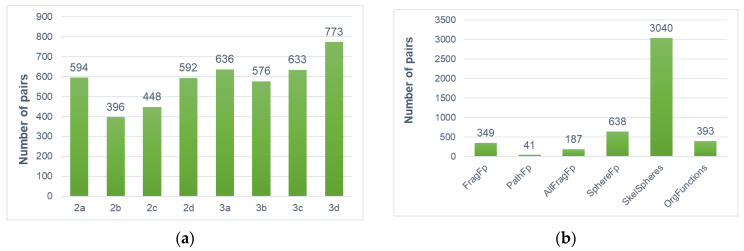
Similarity profile: (**a**) Distribution of similarity pairs between the synthesized compounds (**2a**–**2d** and **3a**–**3d**) and approved drugs from the DrugBank database; (**b**) Number of similarity pairs generated by each fingerprint-based similarity metric used in the analysis.

Figure 6a presents the distribution of similarity pairs between the synthesized compounds (**2a**–**2d** and **3a**–**3d**) and approved drugs from DrugBank, calculated using six fingerprint-based similarity metrics. The number of pairs obtained for each compound reflects its degree of structural similarity to clinically validated molecules.

Figure 6b shows the number of similarity pairs generated by each of the six similarity metrics used in the analysis (FragFp, PathFp, AllFragFp, SphereFp, SkelSpheres, and OrgFunctions). The SkelSpheres fingerprint produced the highest number of similarity pairs, indicating higher sensitivity to scaffold-level similarity, whereas PathFp generated the lowest number of matches, suggesting a stricter structural comparison.

Using the FragFp similarity metric, Omidenepag isopropyl, an antiglaucoma drug, exhibited the highest similarity scores, showing consistent similarity with all compounds from Series 3 (0.63–0.62), while no significant similarity was observed with any member of Series 2.

Notably, five nonsteroidal anti-inflammatory drugs containing the oxicam scaffold (Piroxicam, Lornoxicam, Tenoxicam, Meloxicam, and Isoxicam) also displayed high similarity values with Series 3 compounds, but not with those from Series 2. These results indicate that Series 3 compounds share structural features compatible with the benzothiazine core, whereas Series 2 compounds appear to lack this structural similarity. In contrast, the compounds from Series 2, but not those from Series 3, showed high similarity values with Parecoxib, as well as with antibacterial sulfonamide drugs such as Phthalylsulfathiazole and Sulfamethoxazole.

The PathFp similarity metric produced only a small number of highly similar matches. Under this metric, Series 2 compounds showed the strongest similarity to Rofecoxib, whereas Series 3 compounds were most similar to the diuretic drug Indapamide.

The AllFragFp, which considers all possible substructural fragments of the molecules, allows the detection of partial structural resemblance between compounds with different cores. The compounds from Series 3 had higher similarity values when compared with those from Series 2. The most similar drugs in terms of structure were: Deferasirox, Indapamide, Vardenafil, Taletrectinib, and Celecoxib. For Series 2, Rofecoxib presented significant similarity.

SphereFp evaluates local atomic environments, and since the similarity values obtained with this metric were relatively low, the detected matches likely correspond to small common structural portions, indicating that the compared molecules share only limited local features, while their overall structures differ significantly. For example, compound **2b** showed similarity to drugs containing two 4-chlorophenyl fragments, whereas compound **2c** was more similar to drugs bearing one 4-chlorophenyl and one 4-fluorophenyl substituent.

The SkelSpheres similarity results suggest that Series 2 compounds are structurally closer to sulfone- and diaryl-containing drugs, including several anti-inflammatory and antibacterial derivatives, whereas Series 3 compounds show higher similarity to larger, lipophilic heterocyclic drugs often associated with CNS-active agents and receptor ligands; however, these relationships should be interpreted cautiously due to the topology-based nature of this fingerprint.

According to the OrgFunctions similarity analysis, Series 2 compounds showed functional group patterns similar to sulfone- and diaryl-containing drugs, including sulfonamides and COX inhibitor-like structures, with representative similarities to Dapsone (0.65–0.64) and Etoricoxib (0.63). In contrast, Series 3 compounds exhibited higher similarity to sulfonamide-containing diuretics and lipophilic heterocyclic drugs, with the highest values observed for Indapamide (0.71–0.69) and Vismodegib (0.70631–0.63199), as well as to other receptor-modulating or enzyme-targeting agents such as Boscalid (0.68) and Tolvaptan (0.60), indicating a broader and more functionally diverse pharmacological space.

## 3. Discussion

The present study describes the synthesis and preliminary toxicity evaluation of a new series of heterocyclic derivatives belonging to the oxazol-5(4*H*)-one (**2a**–**d**) and 1,2,4-triazin-6(5*H*)-one (**3a**–**d**) classes containing the 4-chlorophenylsulfonylphenyl and arylidene scaffolds. The choice of these structural motifs was based on the pharmacological relevance of oxazolones, triazinones, and diaryl sulfones. Also, for the construction of these heterocyclic compounds, the choice in our synthesis involved aldehydes containing 4-methoxyphenyl or 4-chloro/fluoro-phenyl fragments was motivated by the presence of the methoxy group and these halogens in numerous drugs, due to the electronic effects and the important role they play in biological activity [62,63].

In agreement with the literature data [14,26,27,37], the NMR spectra of the synthesized compounds showed a single signal for the H-18 proton, confirming the presence of a single geometric isomer, namely the *Z* isomer, which is thermodynamically more stable [27].

The newly synthesized compounds were evaluated in wild-type and *BLH1*-deficient *S. cerevisiae* strains as a preliminary toxicity assay. At 1 mM, the compounds produced a moderate inhibitory effect on yeast growth. Notably, oxazol-5(4*H*)-one **2d**, having the methoxy group grafted onto the benzylidene moiety**,** and triazin-6(5*H*)-ones **3c**, with a fluorine atom, and **3d,** with the methoxy substituent, exhibited significantly higher toxicity in the *BLH1*-deficient strain than in wild-type cells. These findings suggest that the cellular damage induced by these compounds may be alleviated by bleomycin hydrolase; moreover, the combination of the tested compounds with bleomycin inhibited yeast growth more strongly (at least for compounds **2a**–**d** and **3b**), suggesting that these compounds sensitize the cells to bleomycin-induced damage, potentially through mechanisms that require further investigation.

Similarity search against the ChEMBL database showed mainly moderate similarity (50–60%) to known molecules, indicating that the synthesized compounds occupy a partially explored region of chemical space. Oxazolone derivatives (Series 2) had more analogues, suggesting a well-represented scaffold, whereas triazinone derivatives (Series 3) showed fewer matches, including one with no analogue at the selected threshold. The introduction of the phenylhydrazine fragment in Series 3 generated a more extensive heteroaromatic system, which may explain the differences observed in both similarity analysis and biological effects compared to Series 2. This modification enhanced the lipophilicity and the hydrogen-bonding capacity.

The similarity-based biological profile analysis suggested a potential antiproliferative activity for compound **3d**, whereas compounds **2a** and **2b** were predicted to have weak or negligible anticancer effects. These predictions are consistent with the results obtained on *S. cerevisiae*, where **3d** significantly reduced yeast proliferation, while **2a** and **2b** showed only minor effects. The anticancer potential for the Series 3 compounds is also suggested by the similarity observed with approved anticancer drugs, such as Vismodegib and Taletrectinib.

Both the ChEMBL and DrugBank similarity analyses suggested a potential anti-inflammatory effect through COX inhibition, based on the structural similarity with several oxicams and drugs from the coxib class. These findings may indicate a possible selectivity toward COX-2.

Similarity to compounds from ChEMBL or DrugBank can suggest possible pharmacological profiles, but such predictions are indirect and depend on the chosen fingerprint method. Structural resemblance does not necessarily imply identical biological activity, and the predicted relationships should therefore be considered as hypotheses rather than proof of a specific mechanism of action. However, the consistent trends observed in the analogy analysis, together with the biological data, may help guide the future development of these derivatives. The structural profile of Series 2 compounds, which showed closer relationships to sulfonamide and anti-inflammatory drugs, suggests that this series may be better suited for further investigation as potential anti-inflammatory agents, whereas the higher structural novelty and similarity of Series 3 derivatives to heterocyclic drugs with reported anticancer activity indicate that this series may represent a more promising scaffold for the development of anticancer compounds.

## 4. Materials and Methods

### 4.1. Chemistry

#### 4.1.1. General Information for the Synthesis and Characterization of New Compounds

All chemicals were purchased from Merck (Darmstadt, Germany). Melting points are uncorrected and were determined using a Boëtius hot-plate microscope (VEB Wägetechnik Rapido, PHMK 81/3026, Radebeul, Germany). Elemental analysis was performed using a Perkin-Elmer 2400 Series II CHNS/O elemental analyzer (Waltham, MA, USA). IR spectra (KBr pellets) were recorded with a Vertex 70 spectrometer (Bruker Optik GmbH, Ettlingen, Germany). The relative intensity of bands is abbreviated as follows: vs—very strong; s—strong; m—medium; w—weak. NMR spectra were registered on a Gemini 300 BB spectrometer (Varian, Inc., Palo Alto, CA, USA) at 300 MHz for ^1^H-NMR and 75 MHz for ^13^C-NMR at room temperature, using DMSO-*d*_6_ or CDCl_3_ + TFA as solvent. The values of chemical shifts (*δ*), reported relative to tetramethylsilane as an internal standard, were expressed in ppm, and the coupling constants (*J*) in Hz. The abbreviation of signal multiplicity is as follows: s, singlet; d, doublet; dd, doublet of doublets; t, triplet; m, multiplet; b, broad. The mass spectra were recorded using a triple-quadrupole Varian 1200 LC/MS/MS mass spectrometer (Varian, Inc., Walnut Creek, CA, USA) with an APCI interface. Chloroform solutions of obtained compounds (0.5 mg/mL) were diluted 10-fold with methanol, adding 1% formic acid for oxazolones and 1% ammonium carbonate for triazinones. Compounds **2** were positively ionized while **3** were negatively ionized using a Reodyne 7725 solution injection system at a 50 µL/min flow rate. The resulting molecular ions were fragmented at 1.5 mTorr in an argon collision cell.

#### 4.1.2. General Procedure for the Preparation of 4-(4-R-Benzylidene)-2-(4-(4-chlorophenylsulfonyl)phenyl)oxazol-5(4*H*)-ones **2a**–**d**

To 2-(4-(4-chlorophenylsulfonyl)benzamido)acetic acid **1** (2.83 g, 8 mmol) and 4-R-benzaldehyde (8 mmol) mixture, 0.66 g of anhydrous sodium acetate (8 mmol) and 15 mL of acetic anhydride were added. The obtained suspension was heated at reflux for 4 h, under magnetic stirring. Then, 3 mL of ethanol was added, and the mixture was left to stand overnight at low temperature. The obtained precipitate was filtered off, washed with hot water, and then with cold ethanol. The product was recrystallized from ethanol–chloroform (1:2, *v*/*v*).

(*Z*)-4-Benzylidene-2-(4-((4-chlorophenyl)sulfonyl)phenyl)oxazol-5(4*H*)-one (**2a**), pale yellow crystals, m.p. = 241–243 °C; Yield = 80%; FT-IR (KBr, *ν* cm^−1^): 3090 m, 3066 m, 1796 vs, 1773 s, 1653 vs, 1582 m, 1552 m, 1327 vs, 1293 s, 1161 vs, 853 s, 764 s; ^1^H-NMR (DMSO-*d*_6_, *δ* ppm, *J* Hz): 7.41 (s, 1H, H-18), 7.52–7.58 (m, 3H, H-21, H-22, H-23), 7.72 (d, 8.5, 2H, H-14, H-16), 8.01 (d, 8.8, 2H, H-13, H-17), 8.17 (d, 8.5, 2H, H-8, H-10), 8.28 (d, 8.5, 2H, H-7, H-11), 8.24–8.36 (m, 2H, H-20, H-24); ^13^C-NMR (DMSO-*d*_6_, *δ* ppm, *J* Hz): 128.3 (C-8, C-10, C-13, C-17), 129.1 (C-7, C-11, C-21, C-23), 129.9 (C-6 or C-22), 130.1 (C-14, C-16), 131.7 (C-22 or C-6), 132.5 (C-20, C-24), 132.6 (C-18), 132.9 (C-19), 133.1 (C-4),138.5 (C-15), 139.4 (C-12), 144.2 (C-9), 161.7 (C-2), 166.5 (C-5); Anal. (%): Calcd. for C_22_H_14_ClNO_4_S (423.87 g/mol): C, 62.34; H, 3.33; N, 3.30; S, 7.56. Found: C, 62.36; H, 3.45; N, 3.47; S, 7.74; +APCI-MS, *m*/*z* (%): 424 [^35^Cl M + H]^+^, 426 [^37^Cl M + H]^+^, 279 [^35^ClC_6_H_4_SO_2_C_6_H_4_CO]^+^, 281 [^37^ClC_6_H_4_SO_2_C_6_H_4_CO]^+^, 159 [^35^ClC_6_H_4_SO]^+^, 161 [^37^ClC_6_H_4_SO]^+^.

(*Z*)-4-(4-Chlorobenzylidene)-2-(4-((4-chlorophenyl)sulfonyl)phenyl)oxazol-5(4*H*)-one (**2b**), yellow crystals, m.p. = 270–272 °C; Yield = 50%; FT-IR (KBr, *ν* cm^−1^): 3088 m, 3062 w, 3043 w, 1794 vs, 1770 s, 1653 vs, 1588 s, 1547 m, 1324 vs, 1289 s, 1155 vs, 853 s, 764 s; ^1^H-NMR (CDCl_3_ + TFA, *δ* ppm, *J* Hz): 7.46 (s, 1H, H-18), 7.60 (d, 8.8, 2H, H-21, H-23), 7.74 (d, 8.8, 2H, H-14, H-16), 8.03 (d, 8.8, 2H, H-13, H-17), 8.20 (d, 8.2, 2H, H-8, H-10), 8.31 (d, 8.2, 4H, H-7, H-11, H-20, H-24); ^13^C-NMR (CDCl_3_ + TFA, *δ* ppm, *J* Hz): 128.3 (C-13, C-17), 129.6 (C-8, C-10), 129.8 (C-7, C-11, C-19), 130.3 (C-6), 130.5 (C-14, C-16), 130.5 (C-21, C-23), 134.4 (C-20, C-24), 135.2 (C-18), 135.8 (C-15), 137.9 (C-4, C-22), 141.8 (C-12), 144.3 (C-9), 162.1 (C-2), 168.9 (C-5); Anal. (%): Calcd. for C_22_H_13_Cl_2_NO_4_S (458.31 g/mol): C, 57.65; H, 2.86; N, 3.06; S, 7.00. Found: C, 57.54; H, 2.90; N, 3.10; S, 6.88; +APCI-MS, *m*/*z* (%): 458 [^35^Cl M + H]^+^, 460 [^37^Cl M + H]^+^, 279 [^35^ClC_6_H_4_SO_2_C_6_H_4_CO]^+^, 281 [^37^ClC_6_H_4_SO_2_C_6_H_4_CO]^+^, 159 [^35^ClC_6_H_4_SO]^+^, 161 [^37^ClC_6_H_4_SO]^+^.

(*Z*)-2-(4-((4-Chlorophenyl)sulfonyl)phenyl)-4-(4-fluorobenzylidene)oxazol-5(4*H*)-one (**2c**), yellow crystals, m.p. = 251–253 °C; Yield = 50%; FT-IR (KBr, *ν* cm^−1^): 3090 m, 3078 m, 3046 w, 3002 w, 1797 vs, 1773 s, 1657 s, 1596 s, 1579 s, 1329 s, 1296 s, 1163 vs, 1091 s, 848 s, 763 s; ^1^H-NMR (DMSO-*d*_6_, *δ* ppm, *J* Hz): 7.37 (t, 8.7, 2H, H-21, H-23), 7.46 (s, 1H, H-18), 7.73 (d, 8.5, 2H, H-14, H-16), 8.02 (d, 8.5, 2H, H-13, H-17), 8.18 (d, 8.2, 2H, H-8, H-10), 8.31 (d, 8.2, 2H, H-7, H-11), 8.37 (dd, 8.7, 5.8, 2H, H-20, H-24); ^13^C-NMR (DMSO-*d*_6_, *δ* ppm, *J* Hz): 116.2 (d, *J*^3^*_C-F_* = 21.9, C-21, C-23), 128.3 (C-13, C-17), 129.2 (C-8, C-10), 129.6 (C-6), 129.9 (C-19), 130.2 (C-7, C-11), 131.3 (C-18), 132.5 (C-4), 134.1 (C-14, C-16), 135.0 (d, *J*^4^*_C-F_* = 8.9, C-20, C-24), 139.0 (C-15), 139.4 (C-12), 144.23 (C-9), 161.5 (C-2), 163.3 (d, *J*^1^*_C-F_* = 285.7, C-22), 166.5 (C-5); C_22_H_13_ClFNO_4_S (441.86 g/mol): C, 59.80; H, 2.97; N, 3.17; S, 7.26. Found: C, 59.60; H, 3.02; N, 3.16; S, 7.26; +APCI-MS, *m*/*z* (%): 442 [^35^Cl M + H]^+^, 444 [^37^Cl M + H]^+^, 279 [^35^ClC_6_H_4_SO_2_C_6_H_4_CO]^+^, 281 [^37^ClC_6_H_4_SO_2_C_6_H_4_CO]^+^, 159 [^35^ClC_6_H_4_SO]^+^, 161 [^37^ClC_6_H_4_SO]^+^.

(*Z*)-2-(4-((4-Chlorophenyl)sulfonyl)phenyl)-4-(4-methoxybenzylidene)oxazol-5(4*H*)-one (**2d**), yellow-orange crystals, m.p. = 243–245 °C; Yield = 62%; FT-IR (KBr, *ν* cm^−1^): 3089 m, 3060 w, 2944 w, 2841 w, 1794 vs, 1775 s, 1650 vs, 1595 s, 1580 s, 1325 vs, 1292 vs, 1251 vs, 1161 vs, 1026 m, 847 s, 764 vs; ^1^H-NMR (DMSO-*d*_6_, *δ* ppm, *J* Hz): 3.88 (s, OCH_3_), 7.12 (d, 8.8, 2H, H-21, H-23), 7.41 (s, 1H, H-18), 7.77 (d, 8.4, 2H, H-14, H-16), 8.02 (d, 8.4, 2H, H-13, H-17), 8.17 (d, 8.3, 2H, H-8, H-10), 8.28 (bd, 8.8, 2H, H-7, H-11), 8.29 (d, 8.8 Hz, 2H, H-20, H-24); ^13^C-NMR (DMSO-*d*_6_, *δ* ppm, *J* Hz): 55.6 (OCH_3_), 114.7 (C-21, C-23), 125.9 (C-19), 128.3 (C-14, C-16), 128.9 (C-13, C-17), 129.5 (C-8, C-10), 130.0 (C-6, C-7, C-11), 130.2 (C-4), 133.0 (C-18), 134.8 (C-20, C-24), 139.0 (C-15), 139.3 (C-12), 143.8 (C-9), 160.3 (C-22), 162.2 (C-2), 166.6 (C-5); Anal. (%): Calcd. for C_23_H_16_ClNO_5_S (453.89 g/mol): C, 60.89; H, 3.55; N, 3.09; S, 7.06. Found: C, 60.91; H, 3.57; N, 3.00; S, 6.91; +APCI-MS, *m*/*z* (%): 454 [^35^Cl M + H]^+^, 456 [^37^Cl M + H]^+^, 279 [^35^ClC_6_H_4_SO_2_C_6_H_4_CO]^+^, 281 [^37^ClC_6_H_4_SO_2_C_6_H_4_CO]^+^, 159 [^35^ClC_6_H_4_SO]^+^, 161 [^37^ClC_6_H_4_SO]^+^.

#### 4.1.3. General Procedure for the Preparation of 5-(4-R-Benzylidene)-3-(4-(4-chlorophenylsulfonyl)phenyl)-2-phenyl-1,2-dihydro-1,2,4-triazin-6(5*H*)-ones **3a**–**d**

Oxazol-5(4*H*)-one **2** (3.5 mmol) was dissolved in 9 mL of glacial acetic acid. To the resulting solution, 0.35 mL of phenylhydrazine (3.5 mmol) and 43.5 mg of anhydrous sodium acetate (0.53 mmol) were added. The reaction mixture was refluxed for 5 h; then, the obtained precipitate was filtered off and washed with hot water. The product was purified by recrystallization from ethanol.

(*Z*)-5-Benzylidene-3-(4-((4-chlorophenyl)sulfonyl)phenyl)-2-phenyl-1,2-dihydro-1,2,4-triazin-6(5*H*)-one (**3a**), yellow crystals, m.p. = 259–261 °C; Yield = 78%; FT-IR (KBr, *ν* cm^−1^): 3280 m, 3066 w, 3045 w, 1709 vs, 1643 s, 1597 m, 1495 s, 1326 s, 1290 vs, 1157 vs, 822 w, 757s; ^1^H-NMR (DMSO-*d*_6_, *δ* ppm, *J* Hz): 6.72 (d, 7.4, 2H, H-26, H-30), 6.83 (t, 7.4, 1H, H-28), 7.19 (t, 7.4, 2H, H-27, H-29), 7.33 (s, 1H, H-18), 7.44–7.54 (m, 3H, H-21, H-22, H-23), 7.69 (d, 8.5, 2H, H-14, H-16), 8.02 (d, 8.5, 2H, H-13, H-17), 8.12 (d, 8.5, 2H, H-8, H-10), 8.32 (d, 8.5, 2H, H-7, H-11), 8.34–8.38 (m, 2H, H-20, H-24), 8.98 (s, 1H, NH); ^13^C-NMR (DMSO-*d*_6_, *δ* ppm, *J* Hz): 112.5 (C-26, C-30), 120.4 (C-28), 127.7 (C-8, C-10), 128.9 (C-21, C-23), 129.3 (C-27, C-29),129.6 (C-18), 129.8 (C-13, C-17), 130.0 (C-7, C-11), 131.1 (C-14, C-16, C-22), 132.3 (C-6), 132.8 (C-20, C-24), 133.7 (C-5), 136.1 (C-19), 139.1 (C-15), 139.2 (C-12), 143.3 (C-9), 146.2 (C-25), 159.6 (C-3), 168.8 (C-5′); Anal. (%): Calcd. for C_28_H_20_ClN_3_O_3_S (513.99 g/mol): C, 65.43; H, 3.92; N, 8.18; S, 6.24. Found: C, 65.45; H, 3.94; N, 8.20; S, 6.24; −APCI-MS, *m*/*z* (%): 512 [^35^Cl M − H]^−^, 514 [^37^Cl M-H]^−^, 421 [^35^Cl M-H-C_6_H_5_N]^−^, 423 [^37^ClM-H-C_6_H_5_N]^−^.

(*Z*)-5-(4-Chlorobenzylidene)-3-(4-((4-chlorophenyl)sulfonyl)phenyl)-2-phenyl-1,2-dihydro-1,2,4-triazin-6(5*H*)-one (**3b**), yellow crystals, m.p. = 254–256 °C; Yield = 94%; FT-IR (KBr, *ν* cm^−1^): 3285 s, 3088 w, 3070 w, 3033 w, 1721 vs, 1637 vs, 1585 vs, 1495 vs, 1327 vs, 1287 vs, 1158 vs, 844 s, 759 vs; ^1^H-NMR (DMSO-*d*_6_, *δ* ppm, *J* Hz): 6.71 (d, 7.5, 2H, H-26, H-30), 6.83 (t, 7.0, 1H, H-28), 7.19 (t, 7.5, 2H, H-27, H-29), 7.35 (s, 1H, H-18), 7.58 (d, 8.8, 2H, H-21, H-23), 7.71 (t, 8.7, 2H, H-14, H-16), 8.00 (d, 8.7, 2H, H-13, H-17), 8.11 (d, 8.4, 2H, H-8, H-10), 8.32 (d, 8.4, 2H, H-7, H-11), 8.37 (d, 8.8, 2H, H-20, H-24), 9.00 (s, 1H, NH); ^13^C-NMR (DMSO-*d*_6_, *δ* ppm, *J* Hz): 112.5 (C-26, C-30), 120.4 (C-28), 127.7 (C-8, C-10), 128.2 (C-18), 129.0 (C-13, C-17), 129.3 (C-27, C-29), 129.6 (C-7, C-11), 129.9 (C-21, C-23), 130.0 (C-14, C-16), 132.2 (C-6), 132.8 (C-19), 134.3 (C-20, C-24), 135.7 (C-5), 136.4 (C-22), 139.1 (C-15), 139.2 (C-12), 143.3 (C-9), 146.2 (C-25), 160.0 (C-3), 168.7 (C-5′); Anal. (%): Calcd. for C_28_H_19_Cl_2_N_3_O_3_S (548.44 g/mol): C, 61.32; H, 3.49; N, 7.66; S, 5.85. Found: C, 61.33; H, 3.39; N, 7.60; S, 5.83; −APCI-MS, *m*/*z* (%): 546 [^35^Cl,^35^Cl M-H]^−^, 550 [^37^Cl,^37^Cl M-H]^−^, 548 [^35^Cl,^37^Cl M-H]^−^, 455 [^35^Cl,^35^Cl M-H-C_6_H_5_N]^−^, 459 [^37^Cl,^37^Cl M-H-C_6_H_5_N]^−^, 457 [^35^Cl,^37^Cl/^37^Cl,^35^Cl M-H-C_6_H_5_N^−^.

(*Z*)-3-(4-((4-Chlorophenyl)sulfonyl)phenyl)-5-(4-fluorobenzylidene)-2-phenyl-1,2-dihydro-1,2,4-triazin-6(5*H*)-one (**3c**), yellow crystals, m.p. = 259–261 °C; Yield = 89%; FT-IR (KBr, *ν* cm^−1^): 3280 s, 3093 m, 3076 m, 3043 m, 1720 vs, 1637 vs, 1593 vs, 1493 vs, 1327 vs, 1282 vs, 1157 vs, 1089 s, 845 vs, 765 s; ^1^H-NMR (DMSO-*d*_6_, *δ* ppm, *J* Hz): 6.72 (d, 8.1, 2H, H-26, H-30), 6.82 (t, 7.5, 1H, H-28), 7.19 (t, 7.5, 2H, H-27, H-29), 7.37 (s, 1H, H-18), 7.37 (t, 8.7, 2H, H-21, H-23), 7.69 (d, 8.6, 2H, H-14, H-16), 8.00 (d, 8.6, 2H, H-13, H-17), 8.12 (d, 8.5, 2H, H-8, H-10), 8.31 (d, 8.5, 2H, H-7, H-11), 8.45 (dd, 8.7, 5.9, 2H, H-20, H-24), 9.00 (s, 1H, NH); ^13^C-NMR (DMSO-*d*_6_, *δ* ppm, *J* Hz): 112.5 (C-26, C-30), 116.2 (d, *J*^3^*_C-F_* = 21.8, C-21, C-23), 120.4 (C-28), 127.7 (C-8, C-10), 128.5 (C-18), 129.3 (C-27, C-29), 129.6 (C-13, C-17), 129.8 (C-7, C-11), 130.0 (C-14, C-16), 130.5 (C-6), 132.3 (C-19), 135.4 (d, *J*^4^*_C-F_* = 8.3, C-20, C-24), 135.7 (C-5), 139.1 (C-15), 139.2 (C-12), 143.3 (C-9), 146.2 (C-25), 159.6 (C-3), 164.4 (d, *J*^1^*_C-F_* = 251.8, C-22), 168.7 (C-5′); Anal. (%): Calcd. for C_28_H_19_ClFN_3_O_3_S (531.99 g/mol): C, 63.22; H, 3.60; N, 7.90; S, 6.03. Found: C, 63.14; H, 3.68; N, 7.82; S, 5.90; −APCI-MS, *m*/*z* (%): 530 [^35^Cl M-H]^−^, 532 [^37^Cl M-H]^−^, 437 [^35^Cl M-H-C_6_H_5_N]^−^, 439 [^37^Cl M-H-C_6_H_5_N]^−^.

(*Z*)-3-(4-((4-Chlorophenyl)sulfonyl)phenyl)-5-(4-methoxybenzylidene)-2-phenyl-1,2-dihydro-1,2,4-triazin-6(5*H*)-one (**3d**), yellow crystals, m.p. = 264–267 °C; Yield = 95%; FT-IR (KBr, *ν* cm^−1^): 3287 m, 3092 w, 3076 w, 3029 w, 3010 w, 2934 w, 2837 w, 1719 vs, 1632 vs, 1590 vs, 1564 vs, 1327 vs, 1284 vs, 1254 vs, 1160 vs, 1021 s, 845s, 763 s; ^1^H-NMR (DMSO-*d*_6_, *δ* ppm, *J* Hz): 3.86 (s, 3H, OCH_3_), 6.68 (d, 7.5, 2H, H-26, H-30), 6.83 (t, 7.5, 1H, H-28), 7.10 (d, 8.8, 2H, H-21, H-23), 7.19 (t, 7.5, 2H, H-27, H-29), 7.32 (s, 1H, H-18), 7.78 (d, 8.6, 2H, H-14, H-16), 8.00 (d, 8.6, 2H, H-13, H-17), 8.13 (d, 8.8, 2H, H-8, H-10), 8.31 (d, 8.8, 2H, H-20, H-24), 8.32 (d, 8.8, 2H, H-7, H-11), 8.98 (s, 1H, NH); ^13^C-NMR (DMSO-*d*_6_, *δ* ppm, *J* Hz): 55.5 (OCH_3_), 112.4 (C-26, C-30), 114.6 (C-21, C-23), 120.2 (C-28), 126.5 (C-19), 127.7 (C-8, C-10), 128.6 (C-18), 129.3 (C-27, C-29), 129.5 (C-13, C-17), 129.6 (C-7, C-11), 130.0 (C-14, C-16), 132.5 (C-6), 134.0 (C-5), 135.0 (C-20, C-24), 139.1 (C-15, C-12), 142.9 (C-9), 146.3 (C-25), 157.9 (C-22), 161.7 (C-3), 168.6 (C-5′); Anal. (%): Calcd. for C_29_H_22_ClN_3_O_4_S (544.02 g/mol): C, 64.03; H, 4.08; N, 7.72; S, 5.89. Found: C, 64.11; H, 4.05; N, 7.50; S, 5.87; −APCI-MS, *m*/*z* (%): 542 [^35^Cl M-H]^−^, 544 [^37^Cl M-H]^−^, 527 [^35^Cl M-H-CH_3_]^−^, 529 [^37^Cl M-H-CH_3_]^−^.

### 4.2. Toxicity Assessment

#### 4.2.1. Growth Media and Yeast Strains

Yeast strains were pre-grown and maintained between experiments in rich medium, YPD (1% *w*/*v* yeast extract, 2% *w*/*v* peptone, 2% *w*/*v* glucose) or synthetic complete SC (0.67% *w*/*v* yeast nitrogen base with (NH_4_)_2_SO_4_, 2% *w*/*v* glucose, supplemented with the necessary amino acids) [64]. Compounds **2a**–**d** and **3a**–**d** were added to growth medium from stocks in dimethylsulfoxide (DMSO). All reagents were purchased from Merck, Darmstadt, Germany.

The *S. cerevisiae* strains were BY4741 (*MAT*a; *his3Δ1*; *leu2Δ0*; *met15Δ0*; *ura3Δ0*), considered the parental or wild-type (WT) strain, and *blh1Δ* (*MAT*a; *his3Δ1*; *leu2Δ0*; *met15Δ0*; *ura3Δ0*; *blh1::kanMX4*), where the *BLH1* gene open reading frame (ORF) had been replaced with a G418 (geneticin) resistance gene. The strains were obtained from European *S. cerevisiae* Archive for Functional Analysis (EUROSCARF, www.euroscarf.de, accessed on 10 March 2026).

#### 4.2.2. Effect of Compounds on Cell Proliferation

Overnight YPD precultures were inoculated in SC at a density of 10^5^ cells/mL and incubated with shaking (200 rpm) at 28 °C on a multi-amplitude orbital constant temperature shaking incubator (Thermo Max Q 4000, Thermo Fisher Scientific, Marietta, OH, USA) and grown for two hours before compounds were added to the indicated concentration from sterile stocks. The cell density in liquid media was monitored at time intervals by determining the turbidity of the cellular suspension at 600 nm (OD_600_).

#### 4.2.3. Determination of the Inhibitory Concentration of 50% (IC50%) and 90% (IC90%) of Yeast Cell Proliferation

This was completed as described [65]. Yeast cells prepared as above were exposed to various concentrations of the tested compounds for 24 h (28 °C, 200 rpm) before cell proliferation was recorded spectrophotometrically at 600 nm (OD_600_), treated with a logarithmic function to construct a new growth curve, and correlated the log of the colony-forming units (CFU) with the concentrations tested.

#### 4.2.4. Reproducibility of Results and Statistics

All growth experiments were performed at least three times on different days, yielding similar results. All spectrophotometric measurements were conducted in triplicate. For each individual measurement, values were expressed as the mean ± standard error of the mean (SEM). The data were examined using analysis of variance with multiple comparisons (ANOVA) via the statistical software Prism version 6.05 for Windows (GraphPad Software, La Jolla, CA, USA). A one sample *t*-test was used for the statistical analysis of each strain/condition compared with a strain/condition considered as a reference. The differences were considered to be significant when *p* < 0.05.

### 4.3. Structural Similarity Analysis in ChEMBL Database

The eight newly synthesized compounds (**2a**–**d** and **3a**–**d**) were subjected to structural similarity analysis against the ChEMBL database [66] using the Tanimoto coefficient based on the ChEMBL default fingerprint. A threshold of 50% was selected to allow detection of moderate structural resemblance while avoiding excessive retrieval of unrelated compounds. The total number of structurally related compounds, similarity score distributions, and overlap patterns across the target compounds were assessed. Comparative analysis between the two structural series (Series 2 and Series 3) was performed to evaluate differences in chemical space representation and structural novelty.

### 4.4. Structural Similarity Analysis of Approved Drugs

The structural similarity of the target compounds was also evaluated using DataWarrior 6.1.0 [67]. The approved drug dataset was retrieved from DrugBank 6.0 [68] and used as a reference set representing clinically validated molecules. Structural similarity was calculated using the Tanimoto coefficient based on six fingerprint types available in DataWarrior: (i) Fragment (FragFp), (ii) Path fingerprint (PathFp), (iii) AllFragments fingerprint (AllFragFp), (iv) Sphere fingerprint (SphereFp), (v) Skeleton Sphere fingerprint (SkelSpheres), and (vi) Organic Functional Groups (OrgFunctions). The use of multiple similarity metrics reduces bias associated with any single descriptor and provides a more comprehensive assessment of chemical space overlap. Compounds showing high similarity to approved drugs were further examined, as such relationships may indicate potential biological similarities.

## 5. Conclusions

New heterocyclic derivatives from the oxazolone and triazinone classes were prepared and structurally confirmed using ^1^H-, ^13^C-NMR, MS, and IR spectroscopic methods, as well as elemental analyses. The oxazol-5(4*H*)-ones were synthesized by the reaction of a hippuric acid derivative containing the 4-chlorophenylsulfonyl moiety with unsubstituted/substituted benzaldehyde. Heterocycles with a 1,2,4-triazin-6(5*H*)-one ring were obtained by treating oxazol-5(4*H*)-ones with phenylhydrazine. The biological assays performed on *S. cerevisiae* indicated that the compounds are not strongly cytotoxic but can interfere with cellular processes, as demonstrated by the increased sensitivity of *BLH1*-deficient cells and by the combined effect observed when used concomitantly with bleomycin. The similarity analysis against ChEMBL and DrugBank showed that the synthesized compounds occupy a relevant region of bioactive chemical space while maintaining structural novelty. The oxazolone series displayed higher similarity to sulfonamide and anti-inflammatory drugs, whereas the triazinone series was closer to heterocyclic compounds with reported anticancer activity, suggesting different directions for further optimization of the two scaffolds.

## Figures and Tables

**Table 1:** IC50 and IC90 of compounds **2a**–**d** and **3a**–**d** for *Saccharomyces cerevisiae* strain BY4741, calculated after 24 h of exposure to drugs.

Compound	Concentration Range (mM)	IC50 (mM)	IC90 (mM)
**2a**	0.2–10	7.4	8.9
**2b**	0.2–15	2.8	ND ^1^
**2c**	0.2–15	5.5	ND
**2d**	0.2–10	1.25	7.4
**3a**	0.2–12	1.57	9.3
**3b**	0.2–10	1.74	9.8
**3c**	0.2–10	1.55	8.1
**3d**	0.2–10	1.4	7.95
Fluconazole	0.001–1	0.006	0.53

^1^ Not detected.

## Data Availability

The datasets used and/or analyzed during the current study are available from the corresponding authors upon reasonable request.

## Supplementary Materials

The following supporting information can be downloaded at: https://www.mdpi.com/article/10.3390/molecules31101580/s1.

## References

[1] Payamifar S., Abdouss M., Poursattar Marjani A., Sarreshtehdar Aslaheh H. (2025). A Review of the Application of Fibrous Nano-Silica (KCC-1) as Nanocatalysts in the Preparation of Heterocyclic Ring Frameworks. J. Organomet. Chem..

[2] Khan N., Gupta A., Ahamad S., Hussain M.K., Khan M.U., Siddiqui Z.N. (2025). Functionalized Biochar Catalysts: Advancing Green Chemistry in Synthesis of O- and N-Heterocycles. Environ. Res..

[3] Bhat R.M., Hegde V., Shettar A.K., Alam M.R., AlAjmi M.F., Hoskeri J.H., Keri R.S. (2025). Mimics of Benserazide-an Oxazole Derivatives: Synthesis, Molecular Docking/Simulation Study, and Cytotoxicity Assessment on Lung and Cervical Cancer Cell Lines. J. Mol. Struct..

[4] Joshi S., Mehra M., Singh R., Kakar S. (2023). Review on Chemistry of Oxazole Derivatives: Current to Future Therapeutic Prospective. Egypt. J. Basic Appl. Sci..

[5] Patel D., Patel K., Patel S., Patel B., Patel A. (2024). Review on Therapeutic Diversity of Oxazole Scaffold: An Update. ChemistrySelect.

[6] Halimehjani A.Z., Noorakhtar F. (2025). Synthesis of Novel Bisazlactones and Their Applications in the Synthesis of a New Family of Pseudo-Peptide Enamides with Anti-Cancer Properties. Amino Acids.

[7] Panda P.K., Pakeeraiah K., Mal S., Mahapatra M., Bishoyi A.K., Paidesetty S.K. (2025). Design, Synthesis and Computational Approach of Vanillyl–Imidazolidinyl–Sulfamethoxazole Derivatives as Potent Antimicrobial Candidates Tackling Microbial Resistance. RSC Med. Chem..

[8] Algohary A.M., Alhalafi M.H. (2022). Design, Synthesis and Evaluate of Imidazole, Triazine and Metastable Oxazolone Derivatives as Chemosensor for Detecting Metals. J. Saudi Chem. Soc..

[9] Abu-Melha S. (2012). Synthesis, Antimicrobial Evaluation and Spectroscopic Characterization of Novel Imidazolone, Triazole and Triazinone Derivatives. Spectrochim. Acta Part A Mol. Biomol. Spectrosc..

[10] Mavridis E., Bermperoglou E., Pontiki E., Hadjipavlou-Litina D. (2020). 5-(4H)-Oxazolones and Their Benzamides as Potential Bioactive Small Molecules. Molecules.

[11] Kushwaha N., Kushwaha S. (2022). Synthetic Approaches and Biological Significance of Oxazolone Moieties: A Review. Biointerface Res. Appl. Chem..

[12] Wołczański G., Lisowski M. (2018). A General Method for Preparation of N-Boc-Protected or N-Fmoc-Protected α,β-Didehydropeptide Building Blocks and Their Use in the Solid-Phase Peptide Synthesis. J. Pept. Sci..

[13] Ghareeb E.A., Mahmoud N.F.H., El-Bordany E.A., El-Helw E.A.E. (2021). Synthesis, DFT, and Eco-Friendly Insecticidal Activity of Some N-Heterocycles Derived from 4-((2-Oxo-1,2-Dihydroquinolin-3-Yl)Methylene)-2-Phenyloxazol-5(4H)-One. Bioorg. Chem..

[14] Al-Warhi T., Abualnaja M., Abu Ali O.A., Althobaiti F., Alharthi F., Elsaid F.G., Shati A.A., Fayad E., Elghareeb D., Abu Almaaty A.H. (2022). Synthesis and Biological Activity Screening of Newly Synthesized Trimethoxyphenyl-Based Analogues as Potential Anticancer Agents. Molecules.

[15] Almalki A.J., Ibrahim T.S., Taher E.S., Mohamed M.F.A., Youns M., Hegazy W.A.H., Al-Mahmoudy A.M.M. (2022). Synthesis, Antimicrobial, Anti-Virulence and Anticancer Evaluation of New 5(4H)-Oxazolone-Based Sulfonamides. Molecules.

[16] Albelwi F.F., Al-anazi M., Naqvi A., Hritani Z.M., Okasha R.M., Afifi T.H., Hagar M. (2021). Novel Oxazolones Incorporated Azo Dye: Design, Synthesis Photophysical-DFT Aspects and Antimicrobial Assessments with In-Silico and In-Vitro Surveys. J. Photochem. Photobiol..

[17] Saadi L., Adnan S. (2023). Synthesis, Antibacterial and Antioxidant Evaluation of 2-Substituted-4-Arylidene-5(4H)-Oxazolone Derivatives. Indones. J. Chem..

[18] Abdel-Galil E., Moawad E.B., El-Mekabaty A., Said G.E. (2018). Synthesis and Biological Evaluation of New Multifunctional Oxazolone Scaffolds Incorporating Phenyl Benzoate Moiety. J. Heterocycl. Chem..

[19] Fadda A.A., Mohammed R.M., Tawfik E.H., Hammouda M.A.A. (2021). Synthesis and Anticancer Activity of New 2-Aryl-4-(4-Methoxybenzylidene)-5-Oxazolone Scaffolds. Biointerface Res. Appl. Chem..

[20] Nazlı İ.H., Alp S., Topkaya D., Güney Afacan İ., Nalbantsoy A. (2020). Synthesis and Characterization of Two Novel Oxazol-5-Ones Derivatives and Their Multifunctional Properties; PH Sensitivity, Electropolymerizability and Antiproliferative Activity. J. Fluoresc..

[21] Patel U.P., Unjiya P.S., Rathod V.K., Dabhi R.C., Trivedi V.A., Shah M.K. (2025). 2-(3-Iodo-4-Methylphenyl)-4-(Aryl)Oxazol-5(4H)-One Analogs: Synthesis, Anticancer Screening, SAR, in Silico Molecular Docking, and ADMET Studies. ChemistrySelect.

[22] Savariz F.C., Foglio M.A., De Carvalho J.E., Ruiz A.L.T.G., Duarte M.C.T., Da Rosa M.F., Meyer E., Sarragiotto M.H. (2012). Synthesis and Evaluation of New β-Carboline-3-(4-Benzylidene)-4H-Oxazol-5-One Derivatives as Antitumor Agents. Molecules.

[23] Saleem Naz Babari I., Islam M., Saeed H., Nadeem H., Anwer Rathore H. (2024). Pharmacological Investigations of Newly Synthesized Oxazolones and Imidazolones as COX-2 Inhibitors. Saudi Pharm. J..

[24] Mohamed L.W., El-Badry O.M., El-Ansary A.K., Ismael A. (2017). Design & Synthesis of Novel Oxazolone & Triazinone Derivatives and Their Biological Evaluation as COX-2 Inhibitors. Bioorg. Chem..

[25] Kuş C., Uğurlu E., Özdamar E.D., Can-Eke B. (2017). Synthesis and Antioxidant Properties of New Oxazole-5(4H)-One Derivatives. Turk. J. Pharm. Sci..

[26] Ramírez-Ruiz A.M., Ávila-Cossío M.E., Estolano-Cobián A., Cornejo-Bravo J.M., Martinez A.L., Córdova-Guerrero I., Cota-Ramírez B.R., Carranza-Ambriz K.P., Rivero I.A., Serrano-Medina A. (2023). Inhibitory Activity of 4-Benzylidene Oxazolones Derivatives of Cinnamic Acid on Human Acetylcholinesterase and Cognitive Improvements in a Mouse Model. Molecules.

[27] Das Mahapatra A., Queen A., Yousuf M., Khan P., Hussain A., Rehman M.T., Alajmi M.F., Datta B., Hassan M.I. (2022). Design and Development of 5-(4H)-Oxazolones as Potential Inhibitors of Human Carbonic Anhydrase VA: Towards Therapeutic Management of Diabetes and Obesity. J. Biomol. Struct. Dyn..

[28] Hassan A.Y., Abou-Amra E.S., El-Sebaey S.A. (2023). Design and Synthesis of New Series of Chiral Pyrimidine and Purine Analogs as COX-2 Inhibitors: Anticancer Screening, Molecular Modeling, and in Silico Studies. J. Mol. Struct..

[29] Cascioferro S., Parrino B., Spanò V., Carbone A., Montalbano A., Barraja P., Diana P., Cirrincione G. (2017). An Overview on the Recent Developments of 1,2,4-Triazine Derivatives as Anticancer Compounds. Eur. J. Med. Chem..

[30] Rozbicki P., Oğuz E., Wolińska E., Türkan F., Cetin A., Branowska D. (2024). Synthesis and Examination of 1,2,4-Triazine-Sulfonamide Hybrids as Potential Inhibitory Drugs: Inhibition Effects on AChE and GST Enzymes in Silico and in Vitro Conditions. Arch. Pharm..

[31] Ahmed K., Bashir M., Bano R., Sarfraz M., Khan H.U., Khan S., Sharif A., Waseem A., Gilani M.A., Batool K. (2023). Potent Heteroaromatic Hydrazone Based 1,2,4-Triazine Motifs: Synthesis, Anti-Oxidant Activity, Cholinesterase Inhibition, Quantum Chemical and Molecular Docking Studies. J. Mol. Struct..

[32] El-Barbary A.A., Imam D.R., El–Tahawy M.M.T., El-Hallouty S.M., Kheder N.A., Khodair A.I. (2023). Unexpected Synthesis, Characterization, Biological Evaluations, and Computational Details of Novel Nucleosides Containing Triazine-Pyrrole Hybrid. J. Mol. Struct..

[33] Megally Abdo N.Y., Milad Mohareb R., Halim P.A. (2020). Uses of Cyclohexane-1,3-Dione for the Synthesis of 1,2,4-Triazine Derivatives as Anti-Proliferative Agents and Tyrosine Kinases Inhibitors. Bioorg. Chem..

[34] Khodair A.I., El-Barbary A.A., Imam D.R., Kheder N.A., Elmalki F., Ben Hadda T. (2021). Synthesis, Antiviral, DFT and Molecular Docking Studies of Some Novel 1,2,4-Triazine Nucleosides as Potential Bioactive Compounds. Carbohydr. Res..

[35] Verma T., Sinha M., Bansal N. (2019). Triazinone Derivatives as Antibacterial and Antimalarial Agents. Asian Pac. J. Health Sci..

[36] Zaki I., Moustafa A.M.Y., Beshay B.Y., Masoud R.E., Elbastawesy M.A.I., Abourehab M.A.S., Zakaria M.Y. (2022). Design and Synthesis of New Trimethoxylphenyl-Linked Combretastatin Analogues Loaded on Diamond Nanoparticles as a Panel for Ameliorated Solubility and Antiproliferative Activity. J. Enzym. Inhib. Med. Chem..

[37] Nasser Binjawhar D., Al-Salmi F.A., Alghamdi M.A., Alqahtani A.S., Fayad E., Saleem R.M., Zaki I., Youssef Moustafa A.M. (2024). Design, Synthesis, and Biological Evaluation of Newly Synthesized Cinnamide-Fluorinated Containing Compounds as Bioactive Anticancer Agents. ACS Omega.

[38] Salem M.S., El-Helw E.A.E., Derbala H.A.Y. (2020). Development of Chromone–Pyrazole-Based Anticancer Agents. Russ. J. Bioorg. Chem..

[39] Zaki I., Abdelhameid M.K., El-Deen I.M., Abdel Wahab A.H.A., Ashmawy A.M., Mohamed K.O. (2018). Design, Synthesis and Screening of 1, 2, 4-Triazinone Derivatives as Potential Antitumor Agents with Apoptosis Inducing Activity on MCF-7 Breast Cancer Cell Line. Eur. J. Med. Chem..

[40] Kaushik D., Khan S.A., Chawla G. (2010). Design & Synthesis of 2-(Substituted Aryloxy)-5-(Substituted Benzylidene)-3-Phenyl-2,5-Dihydro-1H-[1,2,4] Triazin-6-One as Potential Anticonvulsant Agents. Eur. J. Med. Chem..

[41] Ghanim A.M., Rezq S., Ibrahim T.S., Romero D.G., Kothayer H. (2021). Novel 1,2,4-Triazine-Quinoline Hybrids: The Privileged Scaffolds as Potent Multi-Target Inhibitors of LPS-Induced Inflammatory Response via Dual COX-2 and 15-LOX Inhibition. Eur. J. Med. Chem..

[42] Liu N.W., Liang S., Manolikakes G. (2016). Recent Advances in the Synthesis of Sulfones. Synthesis.

[43] Kast R.E. (2025). UBC4: A Repurposed Drug Regimen for Adjunctive Use During Bladder Cancer Treatment. Biomedicines.

[44] Lovell K.K., Momin R.I., Sangha H.S., Feldman S.R., Pichardo R.O. (2024). Dapsone Use in Dermatology. Am. J. Clin. Dermatol..

[45] Granados A., Cabrera-Afonso M.J., Escolano M., Badir S.O., Molander G.A. (2022). Thianthrenium-Enabled Sulfonylation via Electron Donor-Acceptor Complex Photoactivation. Chem. Catal..

[46] Rashdan H.R.M., Shehadi I.A., Abdelrahman M.T., Hemdan B.A. (2021). Antibacterial Activities and Molecular Docking of Novel Sulfone Biscompound Containing Bioactive 1,2,3-Triazole Moiety. Molecules.

[47] Nematollahi D., Baniardalan M., Khazalpour S., Pajohi-Alamoti M.R. (2016). Product Diversity by Changing the Electrode Potential. Synthesis, Kinetic Evaluation and Antibacterial Activity of Arylsulfonyl-4,4′-Biphenol and Bis-Arylsulfonyl-4,4′-Biphenol Derivatives. Electrochim. Acta.

[48] Mady M.F., Awad G.E.A., Jørgensen K.B. (2014). Ultrasound-Assisted Synthesis of Novel 1,2,3-Triazoles Coupled Diaryl Sulfone Moieties by the CuAAC Reaction, and Biological Evaluation of Them as Antioxidant and Antimicrobial Agents. Eur. J. Med. Chem..

[49] Cabral-Pacheco G.A., Flores-Morales V., Garza-Veloz I., Damián-Sandoval M., Martínez-Flores R.B., Martínez-Vázquez M.C., Delgado-Enciso I., Rodriguez-Sanchez I.P., Martinez-Fierro M.L. (2023). Evaluation of Dapsone and Its Synthetic Derivative DDS-13 in Cancer in Vitro. Exp. Ther. Med..

[50] Lin R., Han J., He Y., Xie L., Gao T., Chen Y., Zhong Y., Ding Q., Cheng K., Yao X. (2025). Design, Synthesis and Biological Evaluation of Dapsone Derivatives with Broad-Spectrum Antiviral Activity. Eur. J. Med. Chem..

[51] Guzmán-Ávila R., Avelar M., Márquez E.A., Rivera-Leyva J.C., Mora J.R., Flores-Morales V., Rivera-Islas J. (2021). Synthesis, In Vitro, and In Silico Analysis of the Antioxidative Activity of Dapsone Imine Derivatives. Molecules.

[52] Kang C., Kim J., Ju S., Park S., Yoo J.W., Yoon I.S., Kim M.S., Jung Y. (2022). Dapsone Azo-Linked with Two Mesalazine Moieties Is a “Me-Better” Alternative to Sulfasalazine. Pharmaceutics.

[53] Barbuceanu S.F., Rosca E.V., Apostol T.V., Socea L.I., Draghici C., Farcasanu I.C., Ruta L.L., Nitulescu G.M., Iscrulescu L., Pahontu E.M. (2023). New Heterocyclic Compounds from Oxazol-5(4H)-One and 1,2,4-Triazin-6(5H)-One Classes: Synthesis, Characterization and Toxicity Evaluation. Molecules.

[54] Roșca E.V., Apostol T.V., Chifiriuc M.C., Pîrcălăbioru G.G., Drăghici C., Socea L.I., Olaru O.T., Nițulescu G.M., Pahonțu E.M., Hrubaru M. (2020). In Silico and Experimental Studies for the Development of Novel Oxazol-5(4h)-Ones with Pharmacological Potential. Farmacia.

[55] Bărbuceanu F., Roşca E.V., Apostol T.V., Şeremet O.C., Drăghici C., Mihai D.P., Negreş S., Niţulescu G.M., Bărbuceanu Ş.F. (2020). New 2-(4-(4-Bromophenylsulfonyl)Phenyl)-4-Arylidene-Oxazol-5(4H)-Ones: Analgesic Activity and Histopathological Assessment. Rom. J. Morphol. Embryol..

[56] Apostol T.-V., Chifiriuc M.C., Socea L.-I., Draghici C., Olaru O.T., Nitulescu G.M., Visan D.-C., Marutescu L.G., Pahontu E.M., Saramet G. (2022). Synthesis, Characterization, and Biological Evaluation of Novel N-{4-[(4-Bromophenyl)Sulfonyl]Benzoyl}-L-Valine Derivatives. Processes.

[57] Apostol T.-V., Chifiriuc M.C., Draghici C., Socea L.-I., Marutescu L.G., Olaru O.T., Nitulescu G.M., Pahontu E.M., Saramet G., Barbuceanu S.-F. (2021). Synthesis, In Silico and In Vitro Evaluation of Antimicrobial and Toxicity Features of New 4-[(4-Chlorophenyl)Sulfonyl]Benzoic Acid Derivatives. Molecules.

[58] Schiketanz I., Draghici C., Saramet I., Balaban A.T. (2002). Aminoketone, Oxazole and Thiazole Synthesis. Part 15.1 2-[4-(4-Halobenzenesulphonyl)-Phenyl]-5-Aryloxazoles. Arkivoc.

[59] Enenkel C., Wolf D.H. (1993). BLH1 Codes for a Yeast Thiol Aminopeptidase, the Equivalent of Mammalian Bleomycin Hydrolase. J. Biol. Chem..

[60] Wang H., Ramotar D. (2002). Cellular Resistance to Bleomycin in Saccharomyces Cerevisiae Is Not Affected by Changes in Bleomycin Hydrolase Levels. Biochem. Cell Biol..

[61] Morris J., Kunkel M.W., White S.L., Wishka D.G., Lopez O.D., Bowles L., Sellers Brady P., Ramsey P., Grams J., Rohrer T. (2023). Targeted Investigational Oncology Agents in the NCI-60: A Phenotypic Systems–Based Resource. Mol. Cancer Ther..

[62] Chiodi D., Ishihara Y. (2024). The Role of the Methoxy Group in Approved Drugs. Eur. J. Med. Chem..

[63] Summerfield C.J.E., Pattison G. (2026). Which Halogen to Choose? Comparing the Effects of Chlorine and Fluorine as Bioisosteric Substituents in Drug Design. Chem. Sci..

[64] Sherman F. (2002). Getting Started with Yeast. Methods Enzymol..

[65] Veiga A., Toledo M.d.G.T., Rossa L.S., Mengarda M., Stofella N.C.F., Oliveira L.J., Gonçalves A.G., Murakami F.S. (2019). Colorimetric Microdilution Assay: Validation of a Standard Method for Determination of MIC, IC50%, and IC90% of Antimicrobial Compounds. J. Microbiol. Methods.

[66] Zdrazil B., Felix E., Hunter F., Manners E.J., Blackshaw J., Corbett S., de Veij M., Ioannidis H., Lopez D.M., Mosquera J.F. (2024). The ChEMBL Database in 2023: A Drug Discovery Platform Spanning Multiple Bioactivity Data Types and Time Periods. Nucleic Acids Res..

[67] Sander T., Freyss J., von Korff M., Rufener C. (2015). DataWarrior: An Open-Source Program for Chemistry Aware Data Visualization and Analysis. J. Chem. Inf. Model..

[68] Knox C., Wilson M., Klinger C.M., Franklin M., Oler E., Wilson A., Pon A., Cox J., Chin N.E.L., Strawbridge S.A. (2024). DrugBank 6.0: The DrugBank Knowledgebase for 2024. Nucleic Acids Res..

