# Supplementary material for: Synthesis, Characterization and Toxicity Evaluation of Some New Heterocyclic Compounds from Oxazole and 1,2,4-Triazine Classes
Source: Molecules. 2026 May 9;31(10):1580. doi: 10.3390/molecules31101580 (PMC13210031; doi:10.3390/molecules31101580)
Supplement: Supplementary file 1 [file molecules-31-01580-s001.zip › molecules-4262149-supplementary.pdf]

molecules

Supplementary Material

Synthesis, Characterization and Toxicity Evaluation of Some New Heterocyclic Compounds from Oxazole and 1,2,4-Triazine Classes

Stefania-Felicia Barbuceanu [1,*], Elena-Valentina Rosca [1], Laura-Ileana Socea [1,*], Lavinia Liliana Ruta [2], Alexandra Carlan [2], Ileana Cornelia Farcasanu [2], Constantin Draghici [3], George Mihai Nitulescu [1], Elena-Mihaela Pahontu [1], Rica Boscencu [1], Octavian Tudorel Olaru [1], Lucian Iscrulescu [1] and Theodora-Venera Apostol [1]

[1] Faculty of Pharmacy, "Carol Davila" University of Medicine and Pharmacy, 6 Traian Vuia Street, 020956 Bucharest, Romania; elenavalentinarosca90@gmail.com (E.-V.R.); george.nitulescu@umfcd.ro (G.M.N.); elena.pahontu@umfcd.ro (E.-M.P.); rica.boscencu@umfcd.ro (R.B.); octavian.olaru@umfcd.ro (O.T.O.); lucian.iscrulescu@umfcd.ro (L.I.); theodora.apostol@umfcd.ro (T.-V.A.)

[2] Department of Organic Chemistry, Biochemistry and Catalysis, Faculty of Chemistry, University of Bucharest, 90–92 Sos. Panduri Street., 050663 Bucharest, Romania; lavinia.ruta@chimie.unibuc.ro (L.L.R.); alexandra.carlan@s.unibuc.ro (A.C.); ileana.farcasanu@chimie.unibuc.ro (I.C.F.)

[3] "C. D. Nenitescu" Institute of Organic and Supramolecular Chemistry Romanian Academy, 202B Splaiul Independenței, 060023 Bucharest, Romania; cst.drag@yahoo.com

* Correspondence: stefania.barbuceanu@umfcd.ro (S.-F.B.); laura.socea@umfcd.ro (L.-I.S.)

 https://doi.org/10.3390/molecules31101580

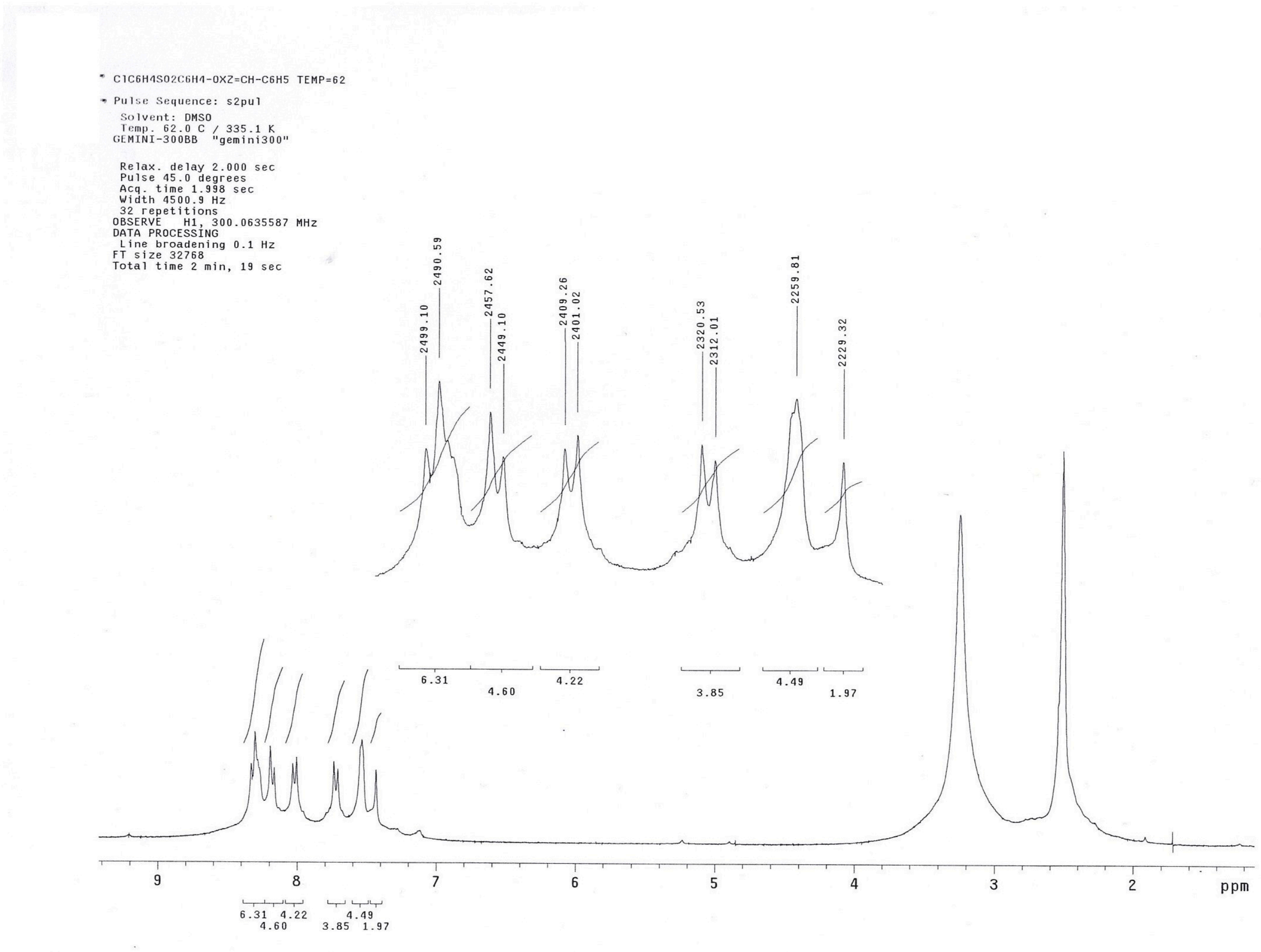

Figure S1. The ^{1}H-NMR spectrum of oxazolone **2a**.

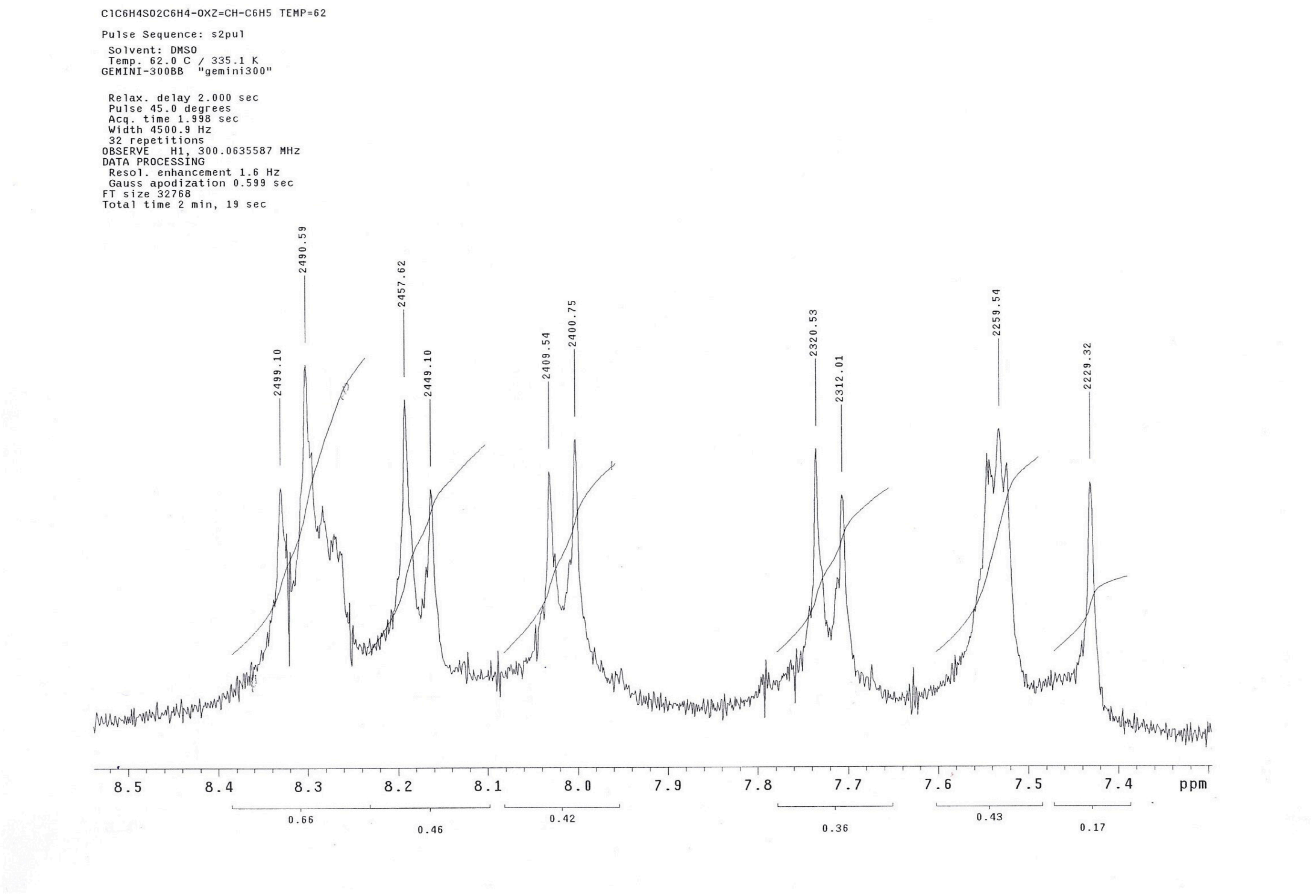

Figure S2. The ^{1}H-NMR spectrum of oxazolone **2a** (resolve).

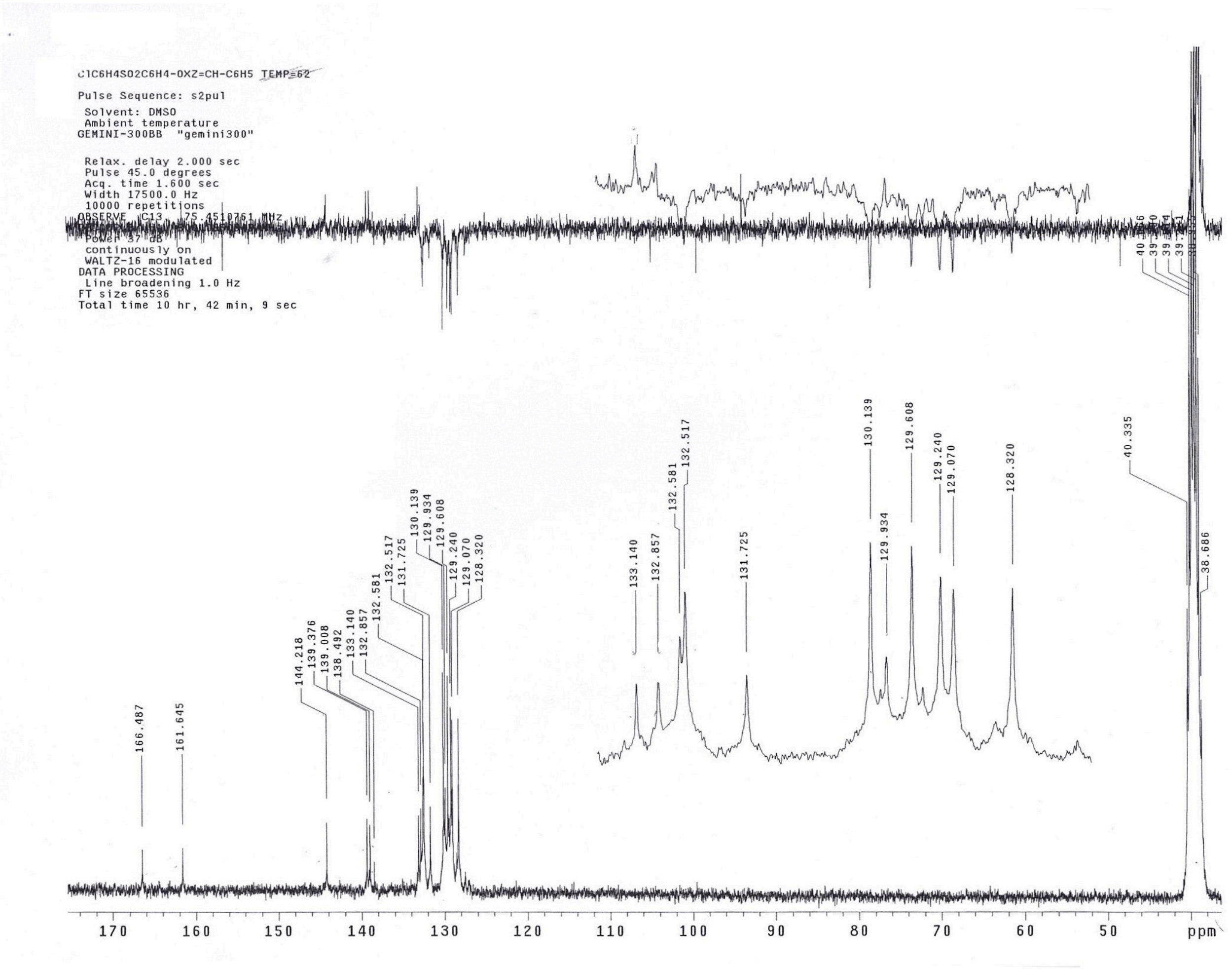

Figure S3. The ^{13}C-NMR spectrum of oxazolone **2a**.

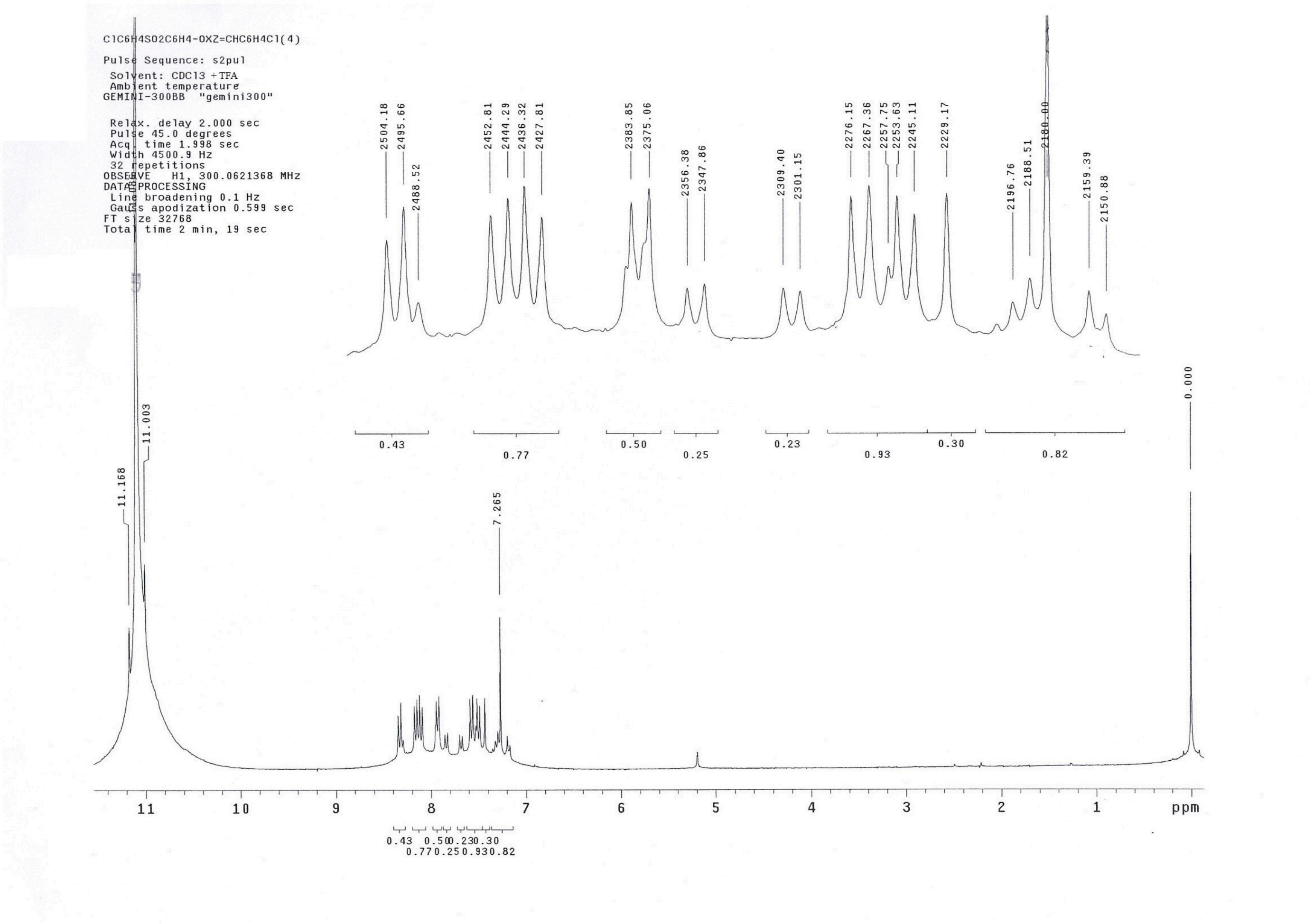

Figure S4. The ^{1}H-NMR spectrum of oxazolone **2b**.

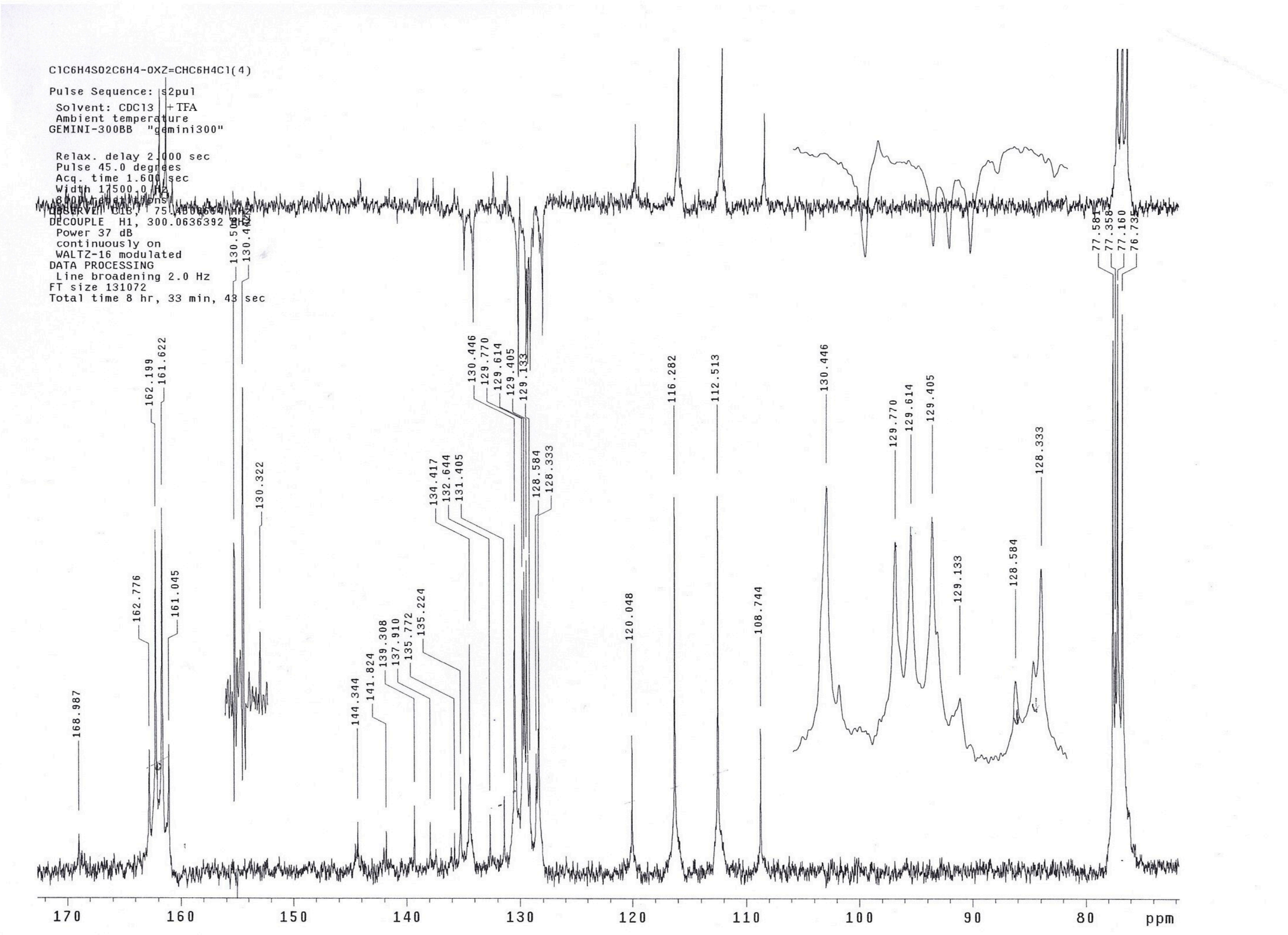

Figure S5. The ^{13}C-NMR spectrum of oxazolone **2b**.

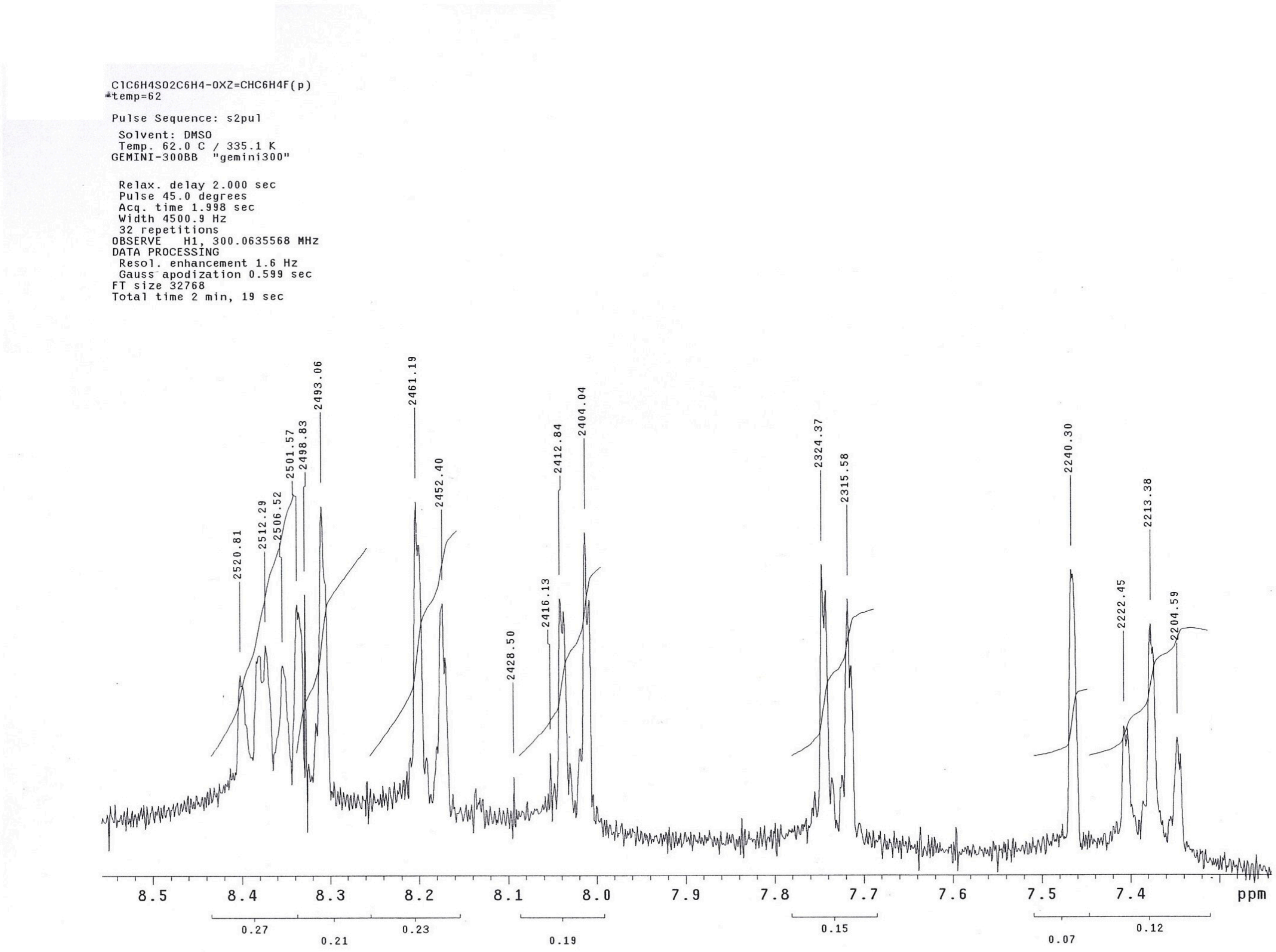

Figure S6. The ^{1}H-NMR spectrum of oxazolone **2c**.

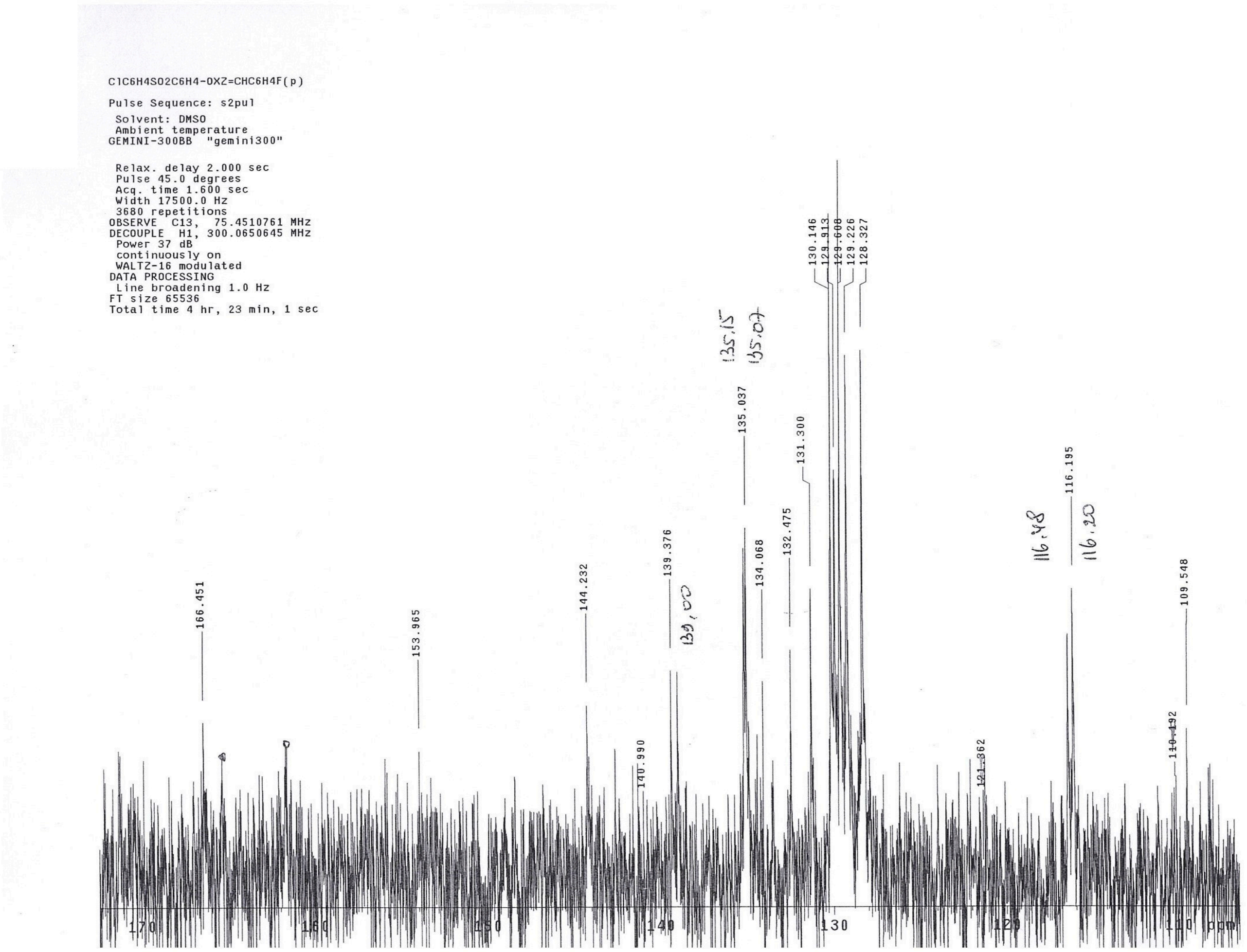

Figure S7. The ^{13}C-NMR spectrum of oxazolone **2c**.

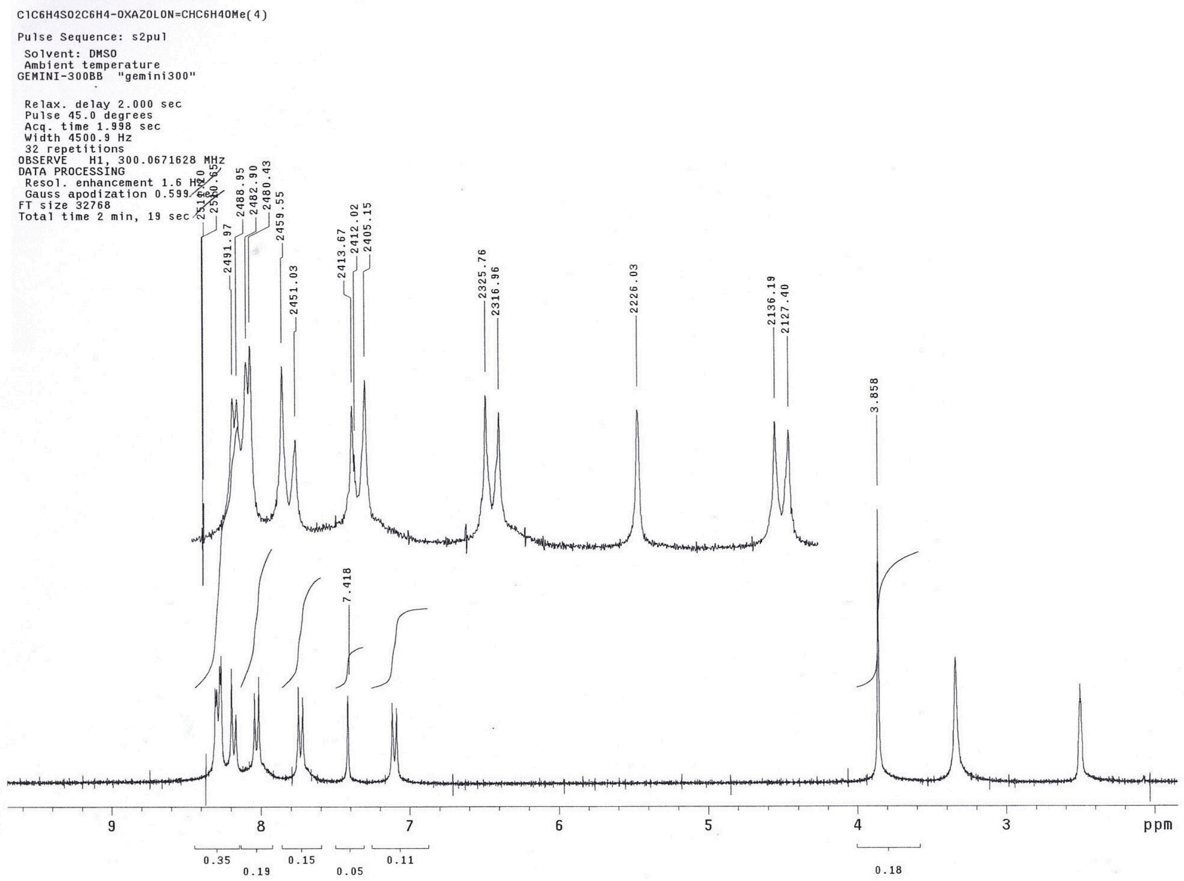

Figure S8. The ^{1}H-NMR spectrum of oxazolone **2d**.

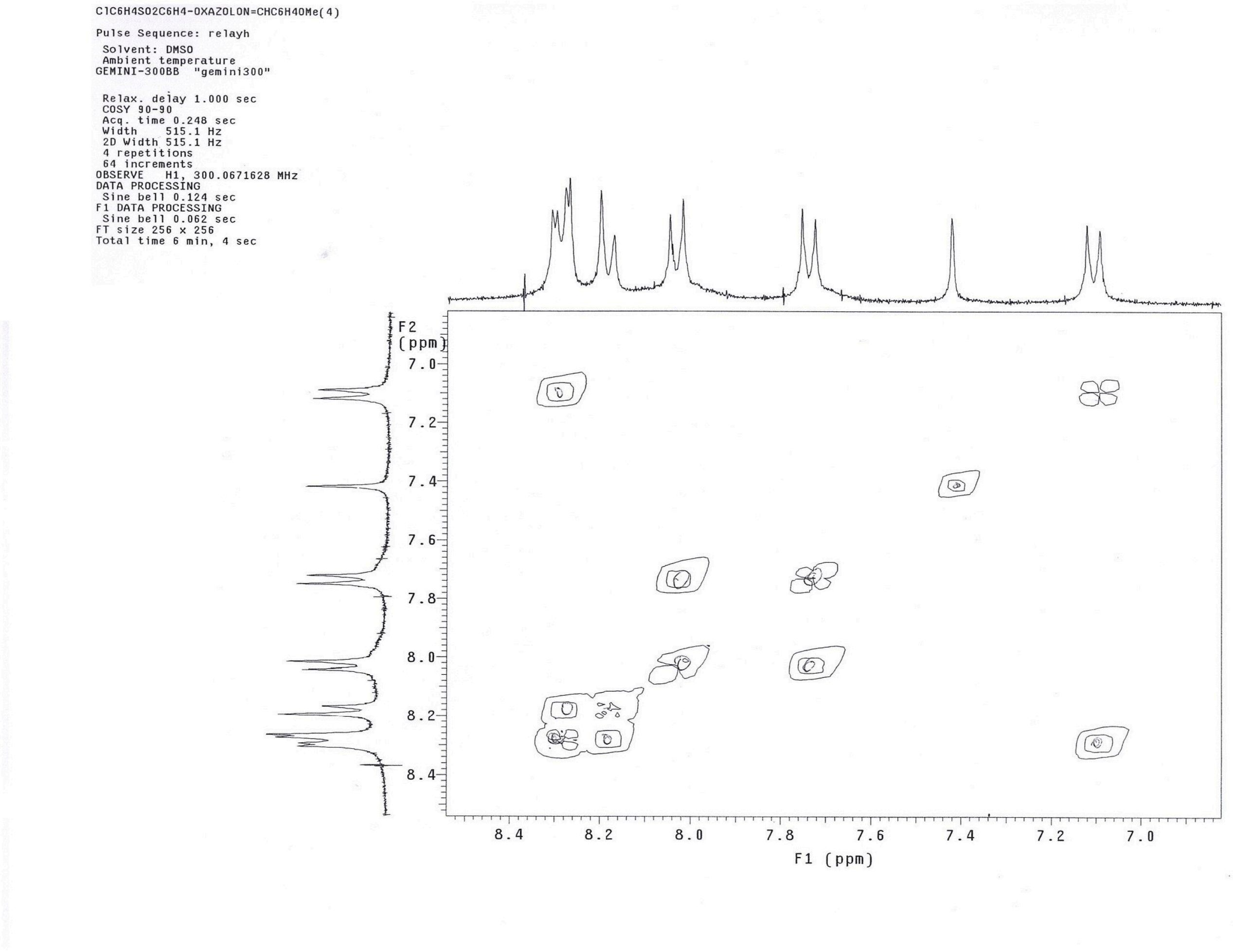

Figure S9. The ^{1}H-^{1}H COSY spectrum of oxazolone **2d**.

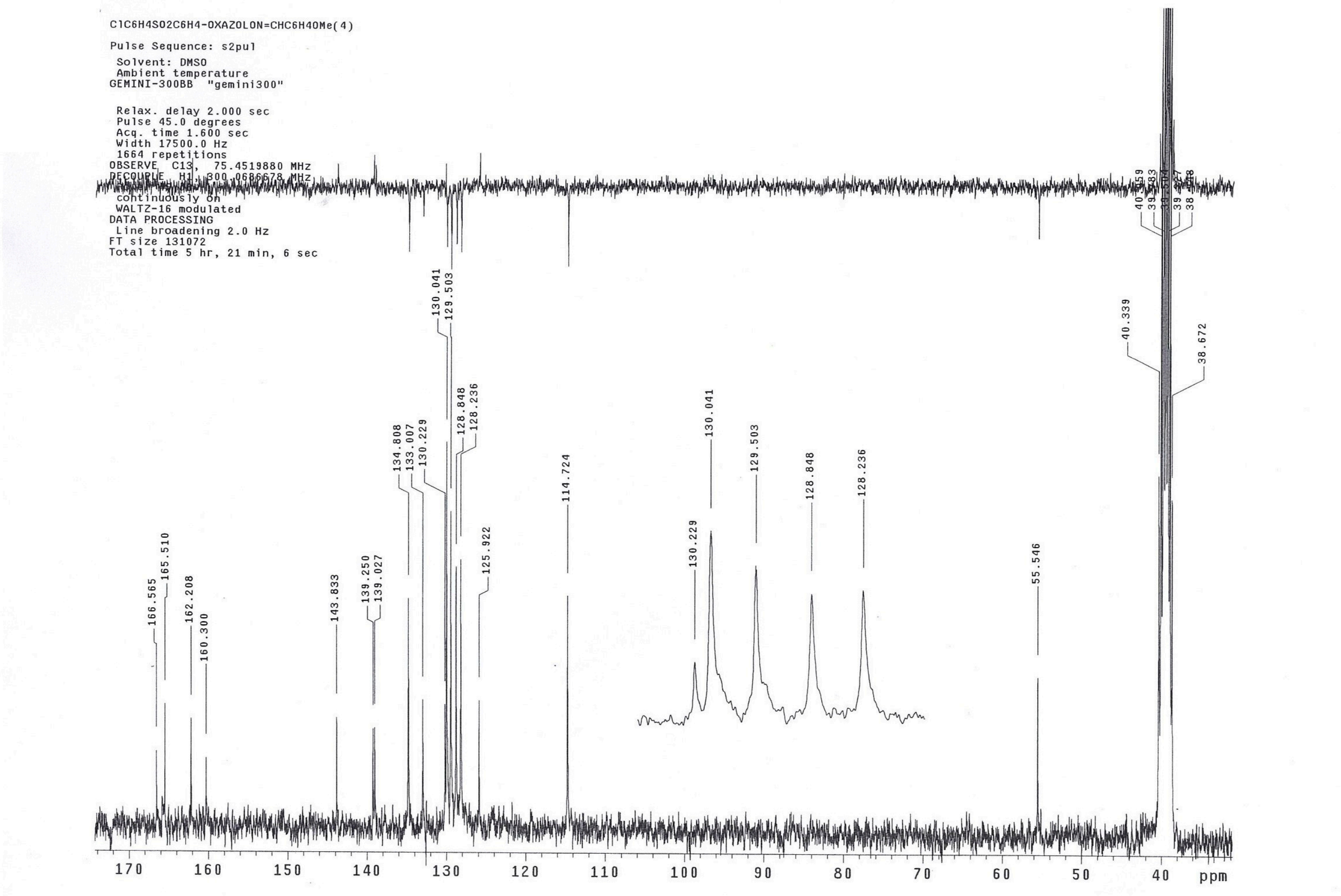

Figure S10. The ^{13}C-NMR spectrum of oxazolone **2d**.

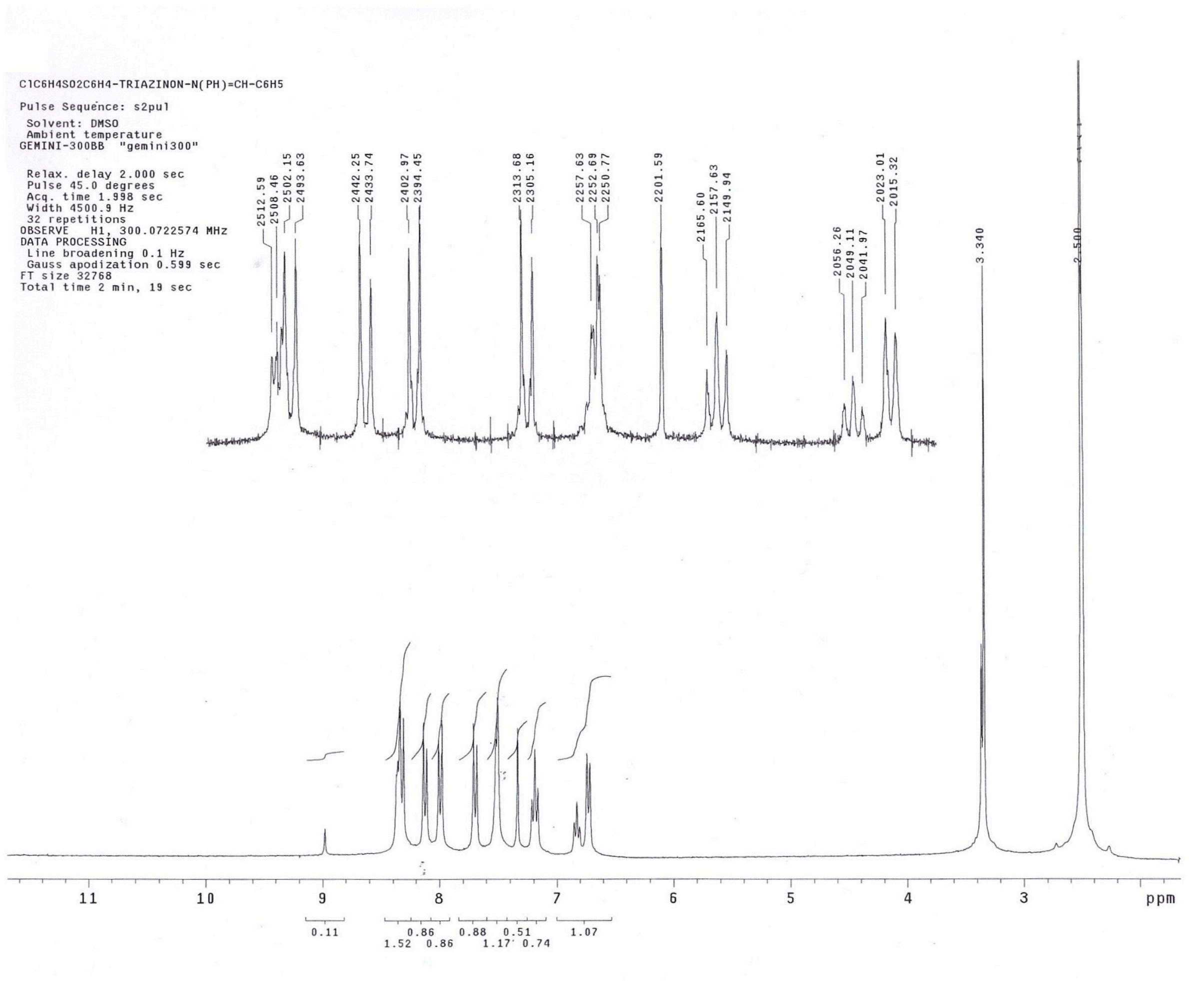

Figure S11. The ^{1}H-NMR spectrum of triazinone **3a**.

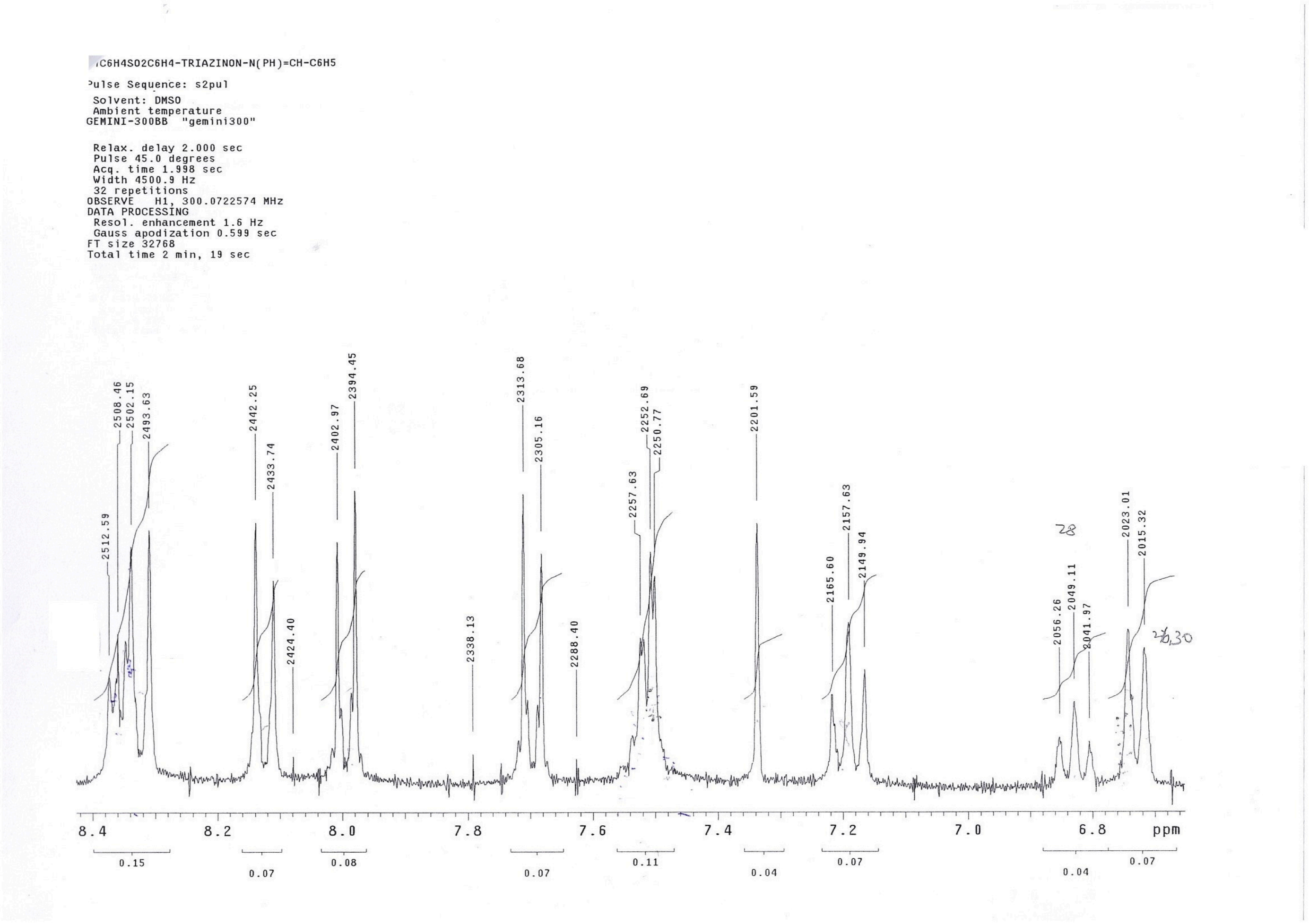

Figure S12. The ^{1}H-NMR spectrum of triazinone **3a** (resolve).

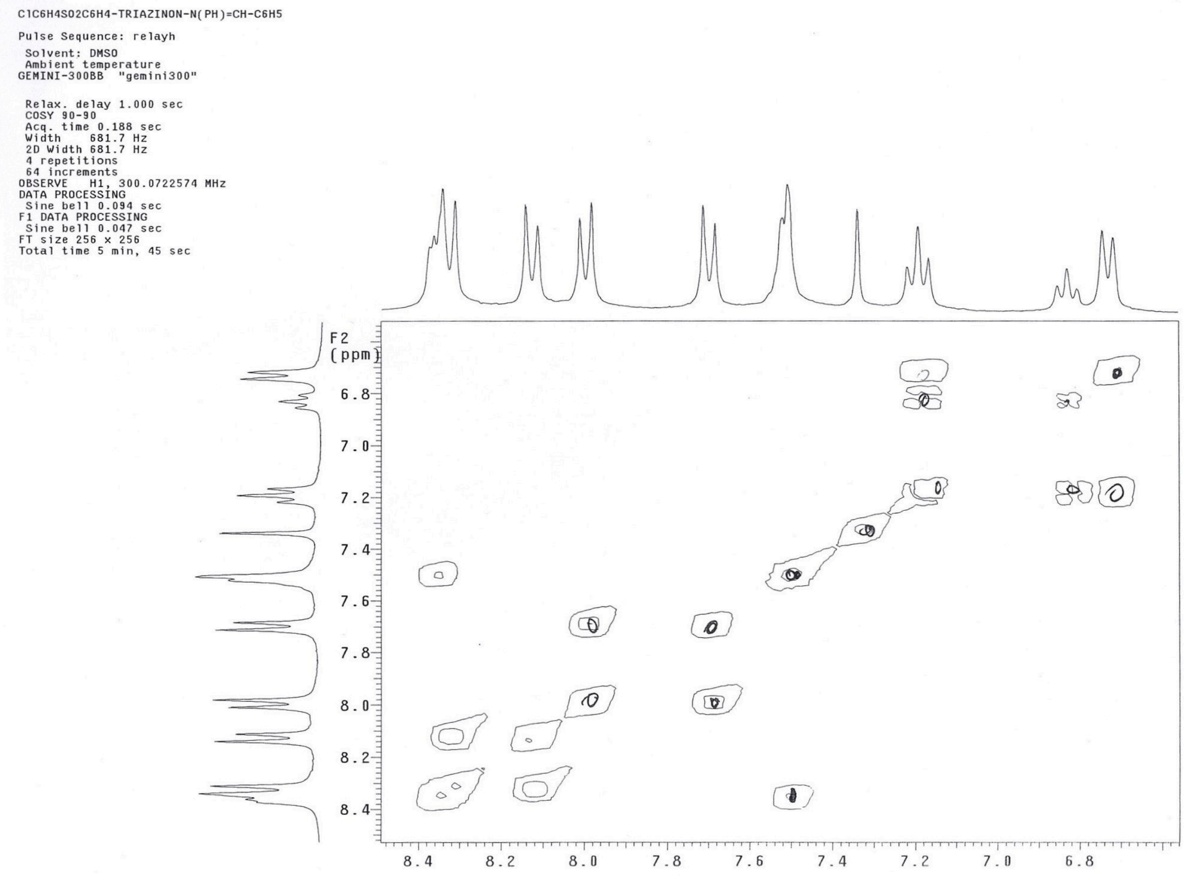

Figure S13. The ^1H-^1H COSY spectrum of triazinone **3a**.

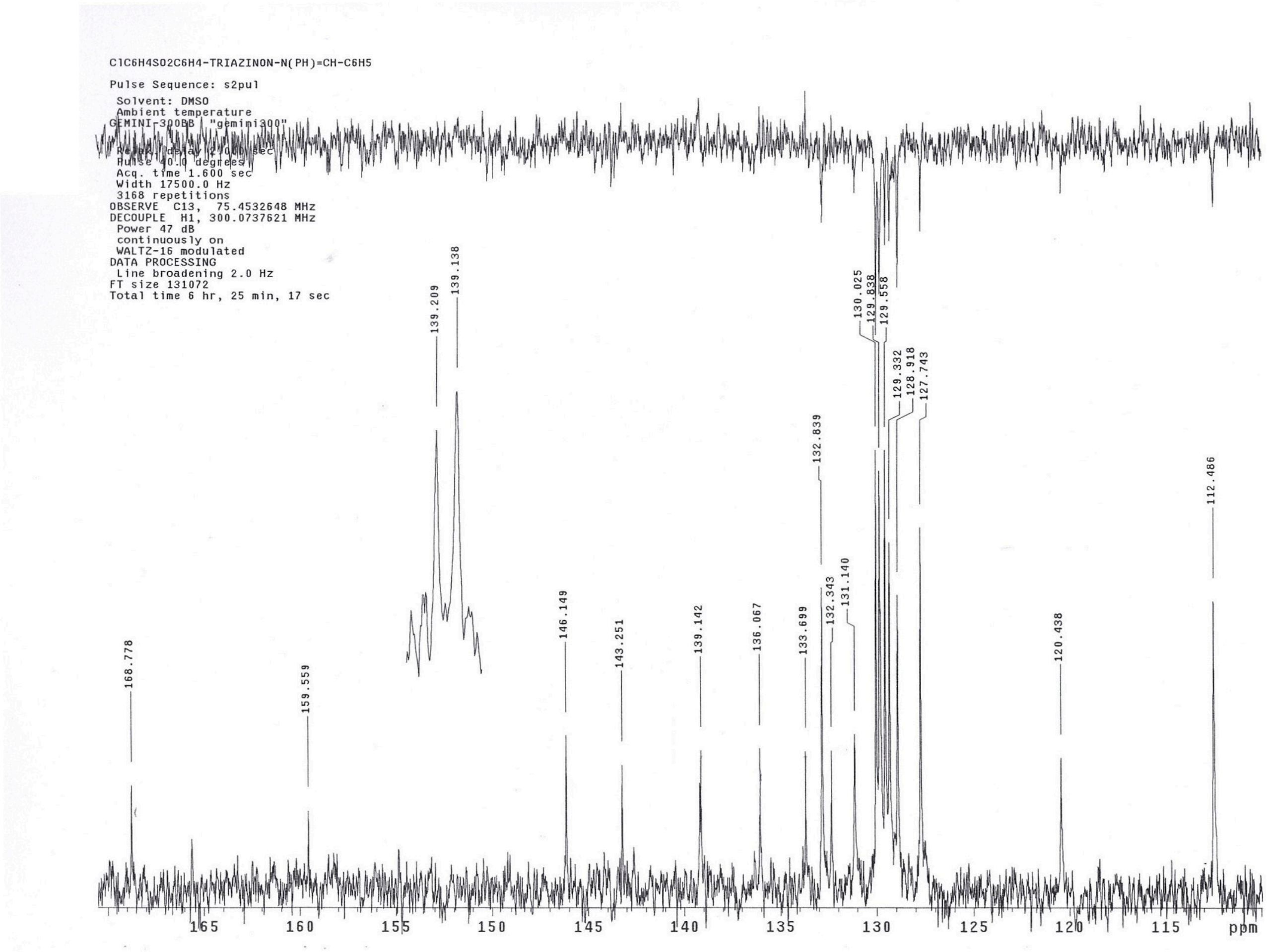

Figure S14. The ^{13}C-NMR spectrum of triazinone **3a**.

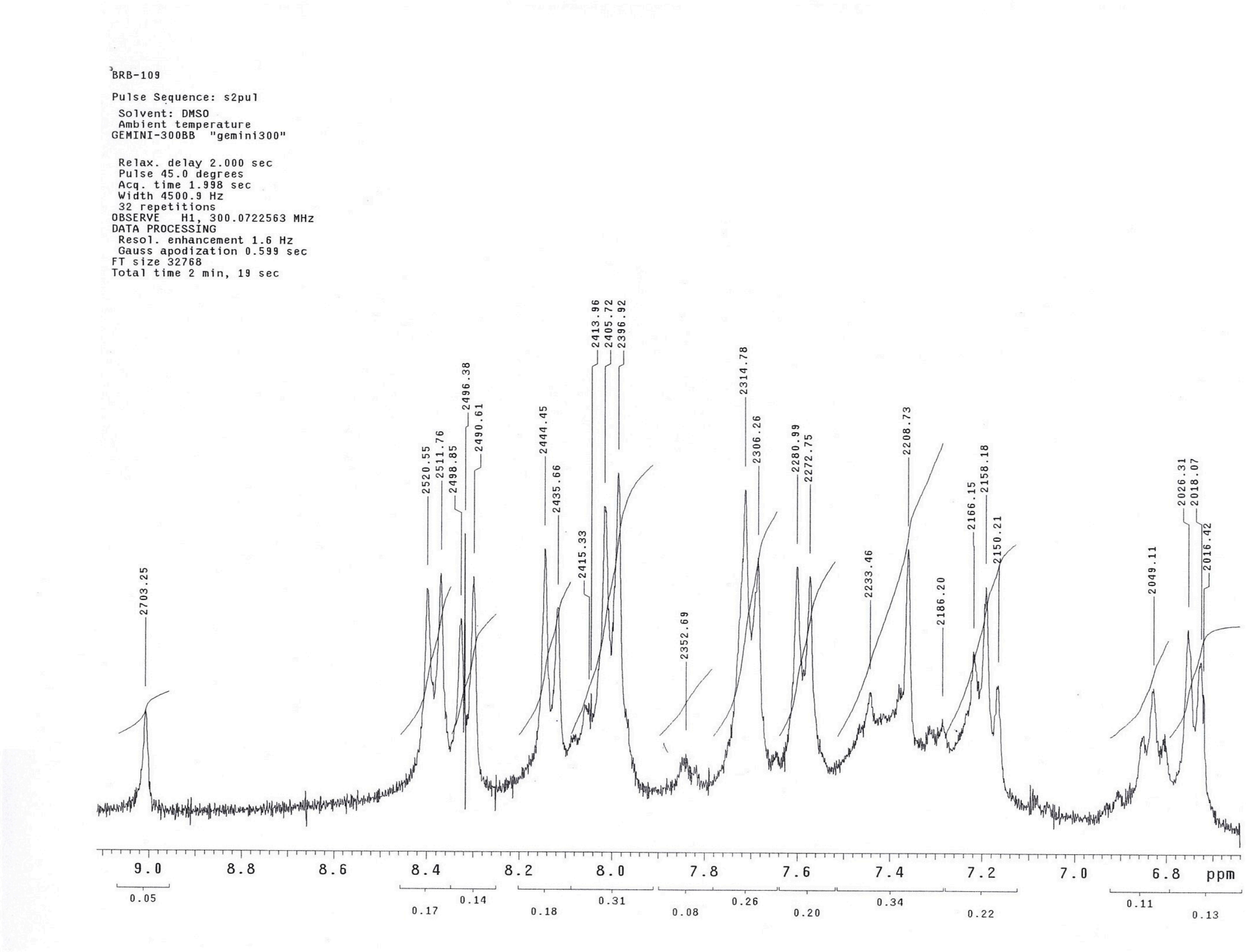

Figure S15. The ^{1}H-NMR spectrum of triazinone **3b**.

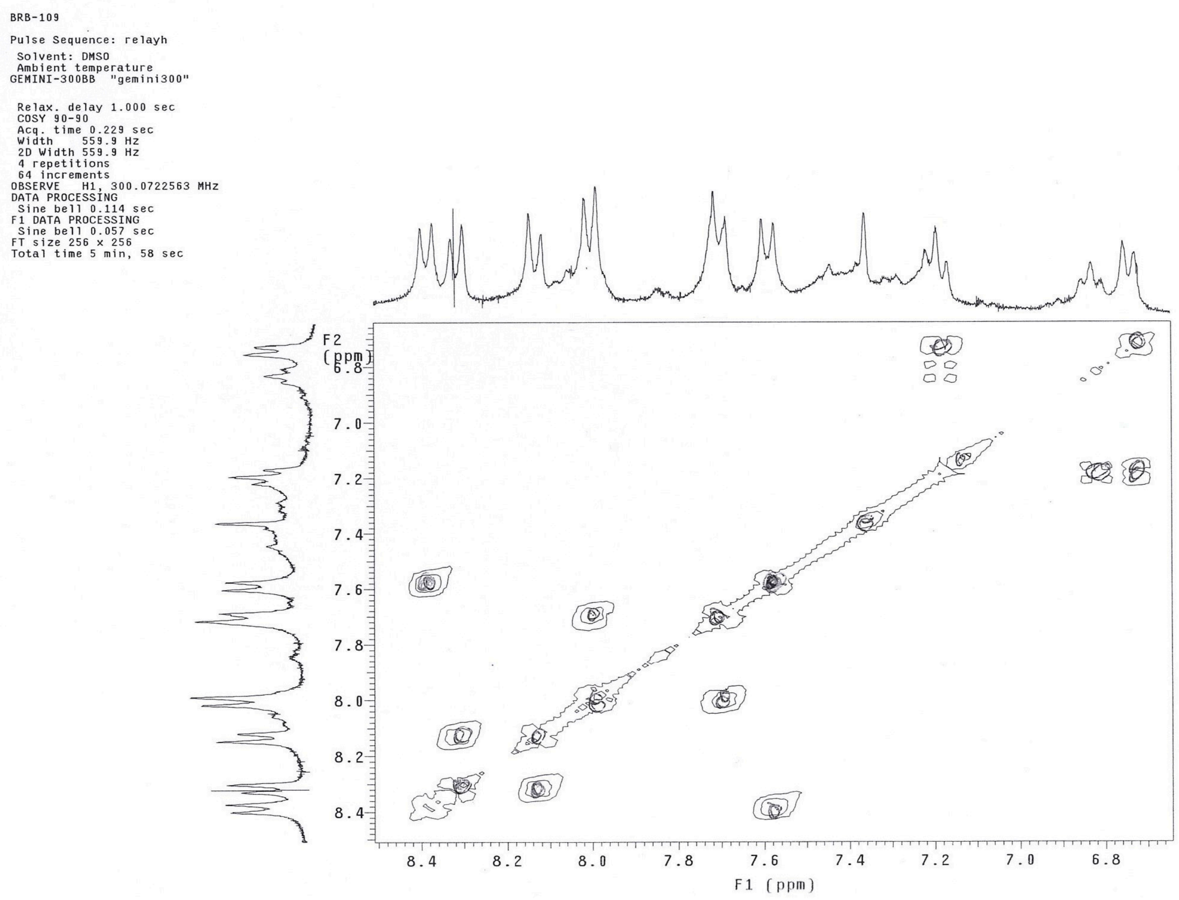

Figure S16. The ^{1}H-^{1}H COSY spectrum of triazinone **3b**.

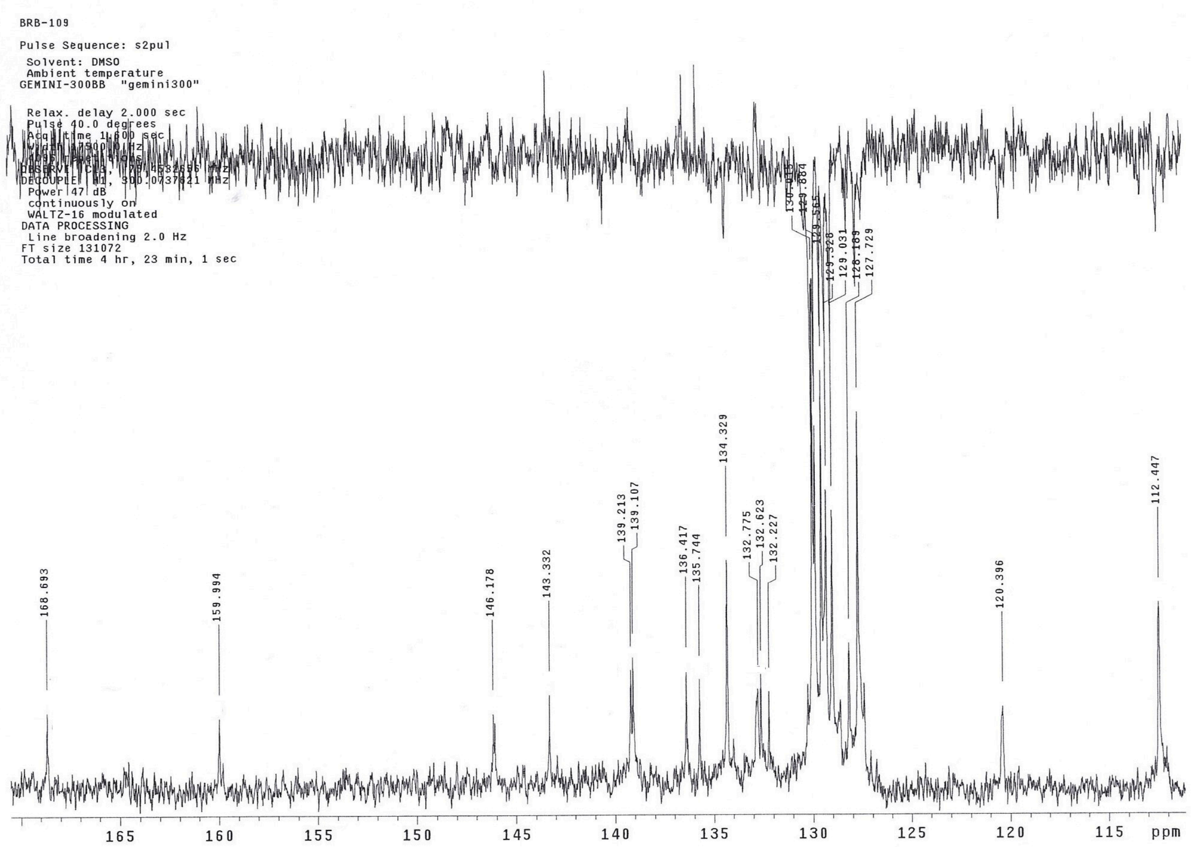

Figure S17. The ^{13}C-NMR spectrum of triazinone **3b**.

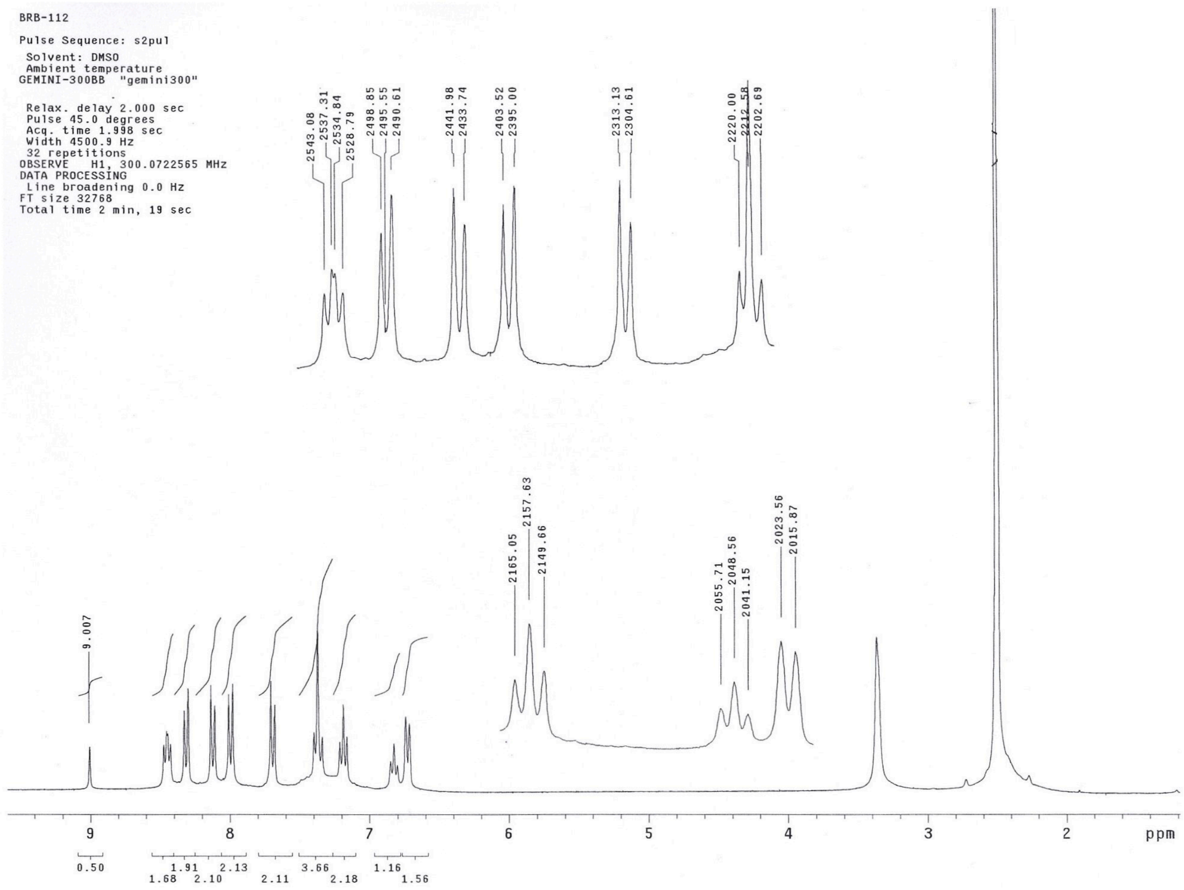

Figure S18. The ^{1}H-NMR spectrum of triazinone **3c**.

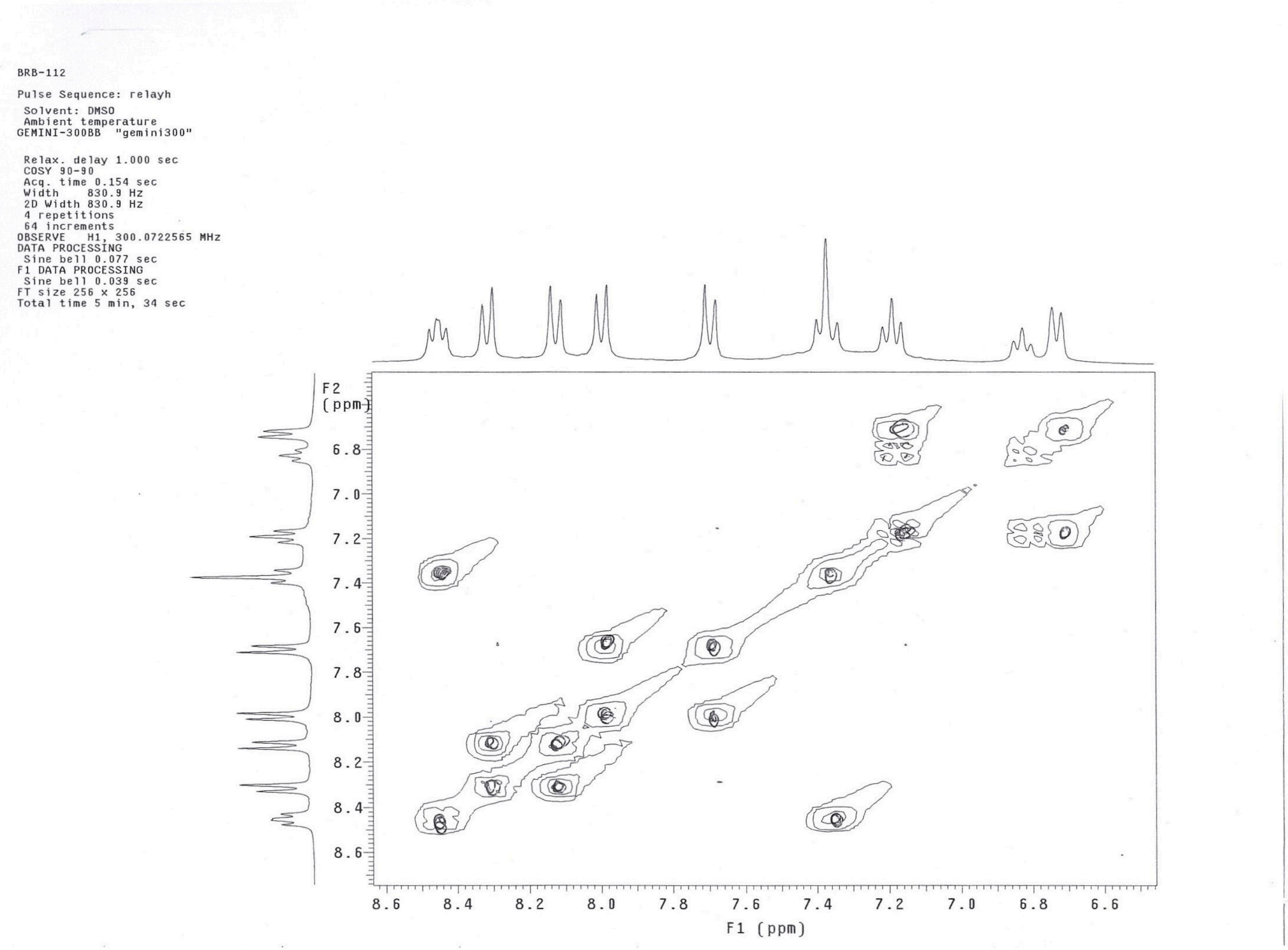

Figure S19. The ^{1}H-^{1}H COSY spectrum of triazinone **3c**.

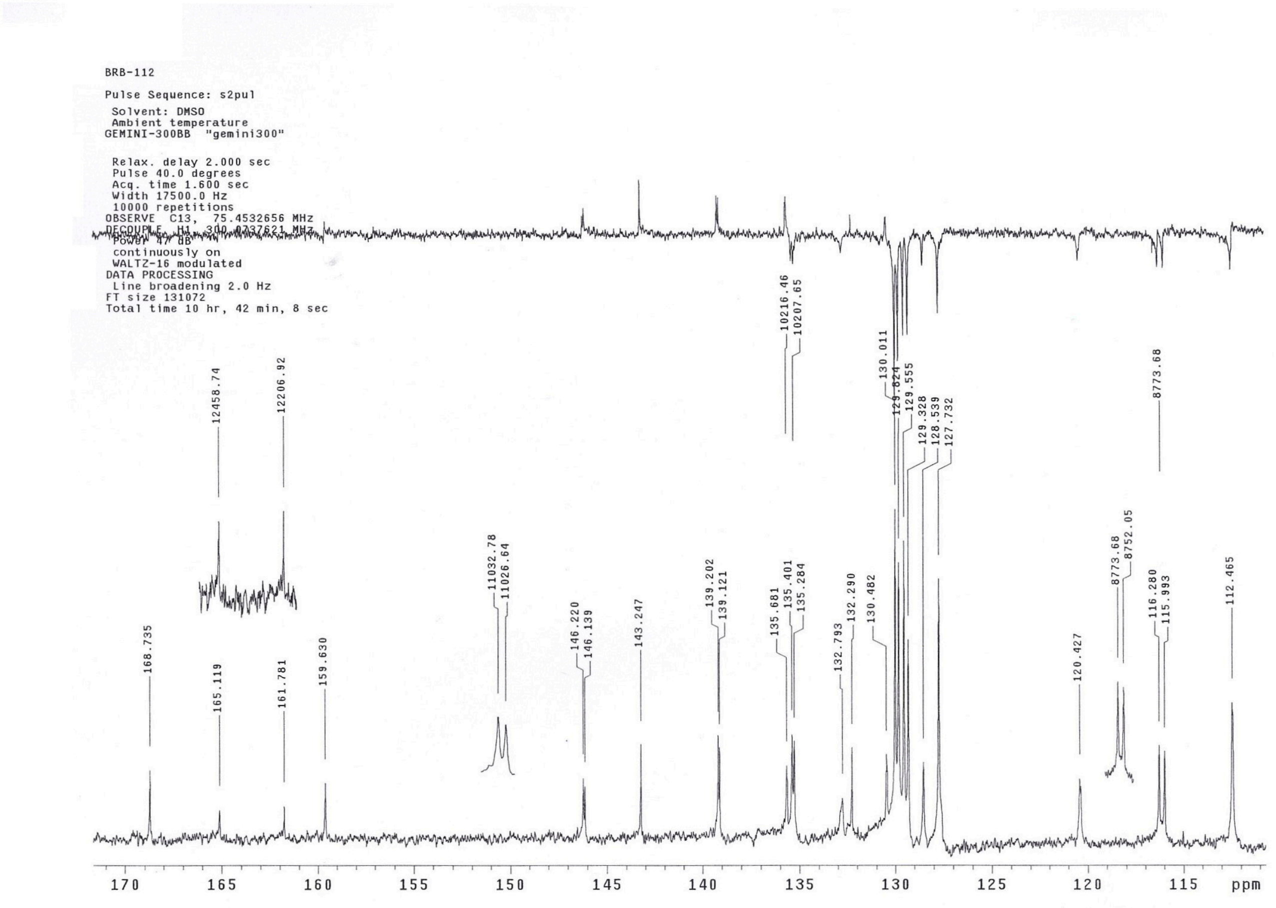

Figure S20. The ^{13}C-NMR spectrum of triazinone **3c**.

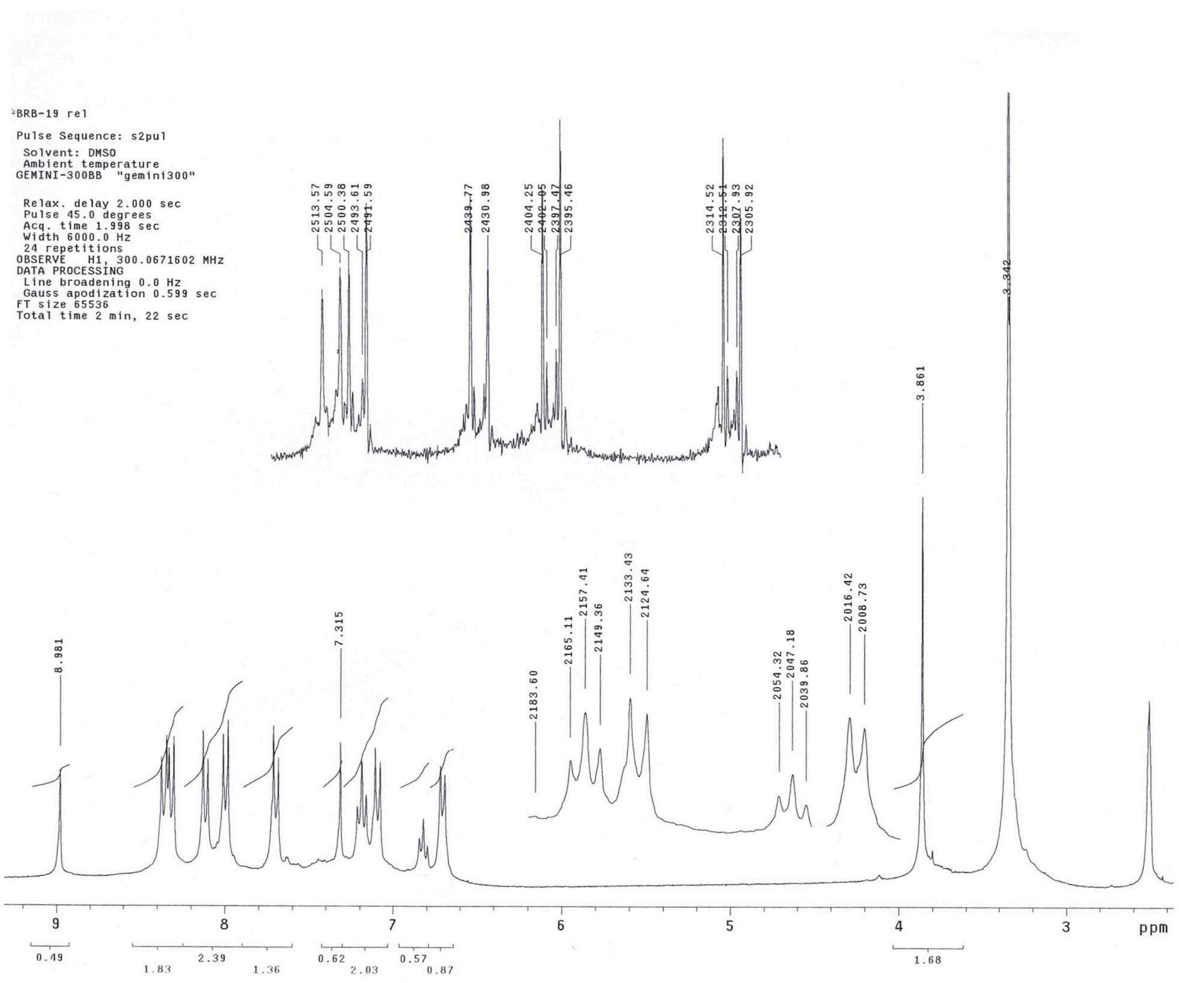

Figure S21. The ^{1}H-NMR spectrum of triazinone **3d**.

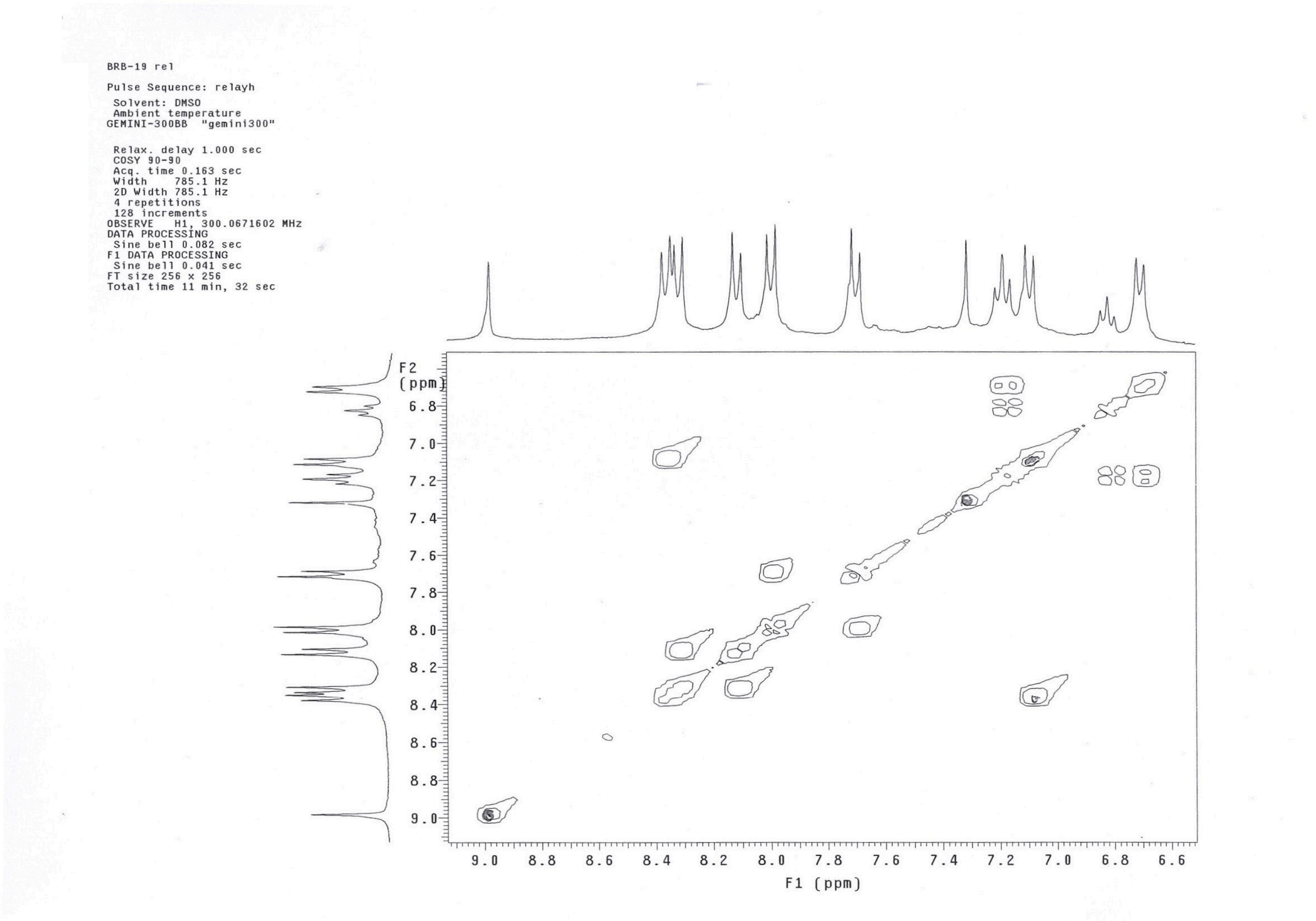

Figure S22. The ^1H-^1H COSY spectrum of triazinone **3d**.

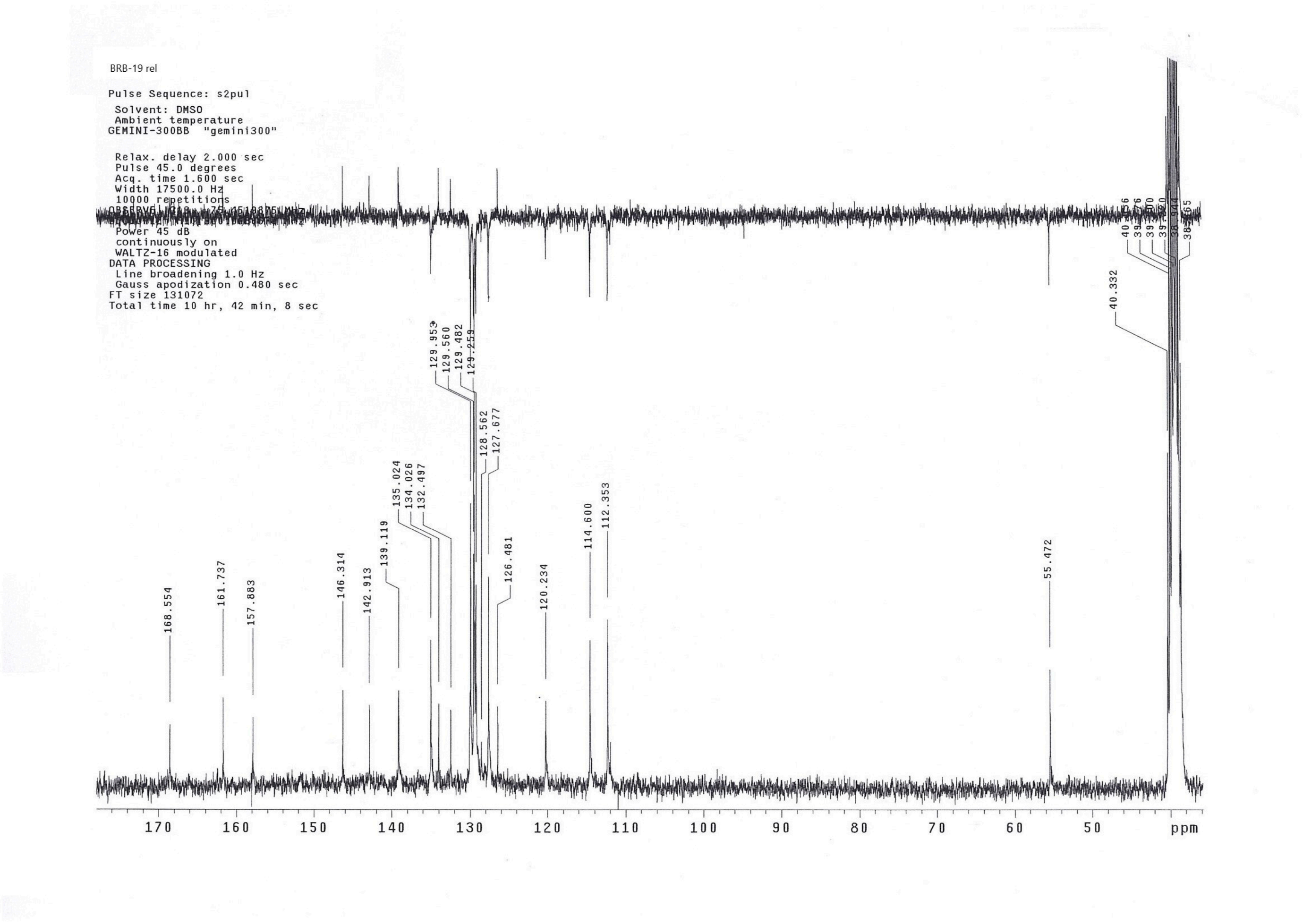

Figure S23. The ^{13}C-NMR spectrum of triazinone **3d**.

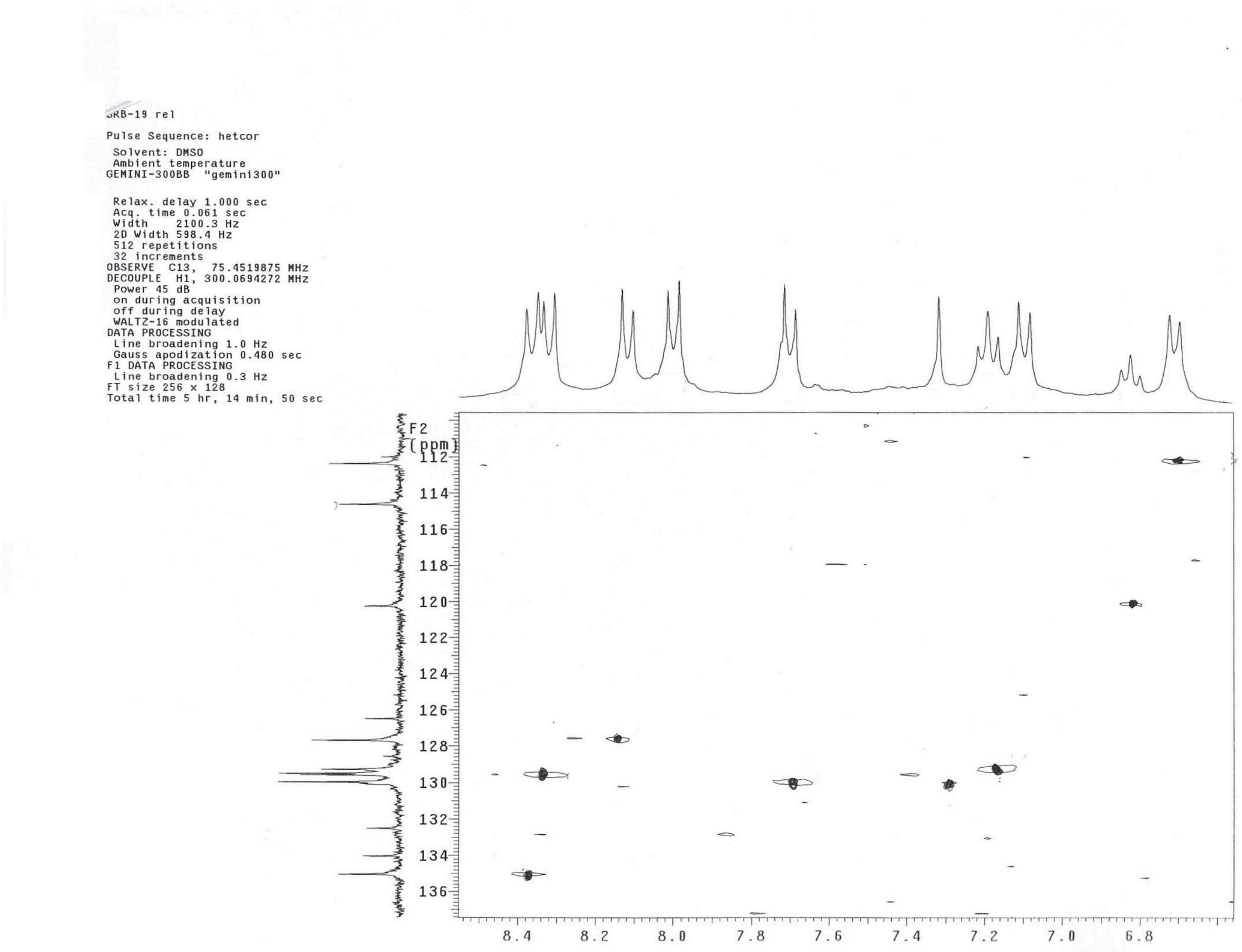

Figure S24. The ^{1}H-^{13}C HETCOR spectrum of triazinone **3d**.

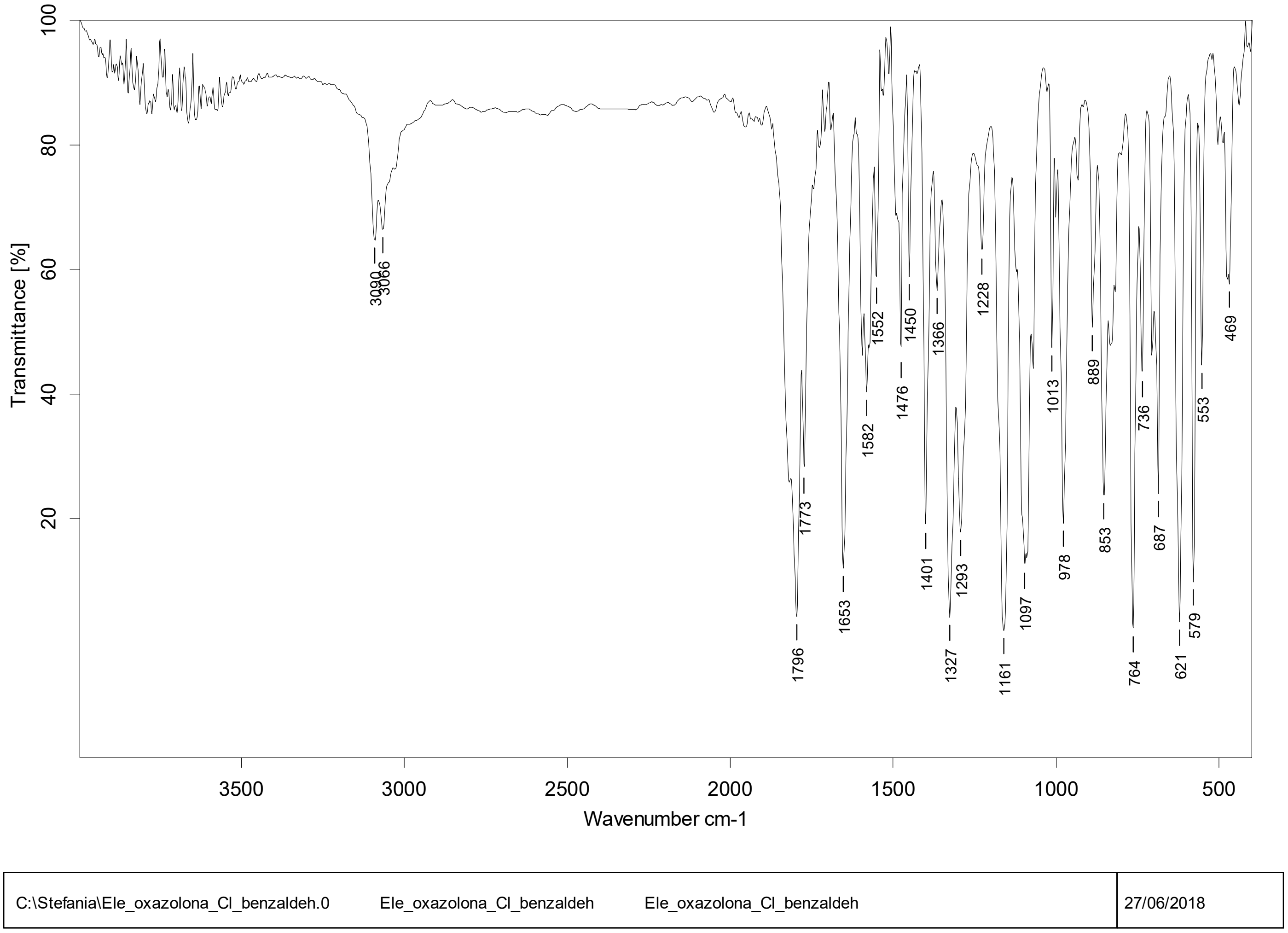

Figure S25. The IR spectrum of oxazol-5(4*H*)-one **2a**.

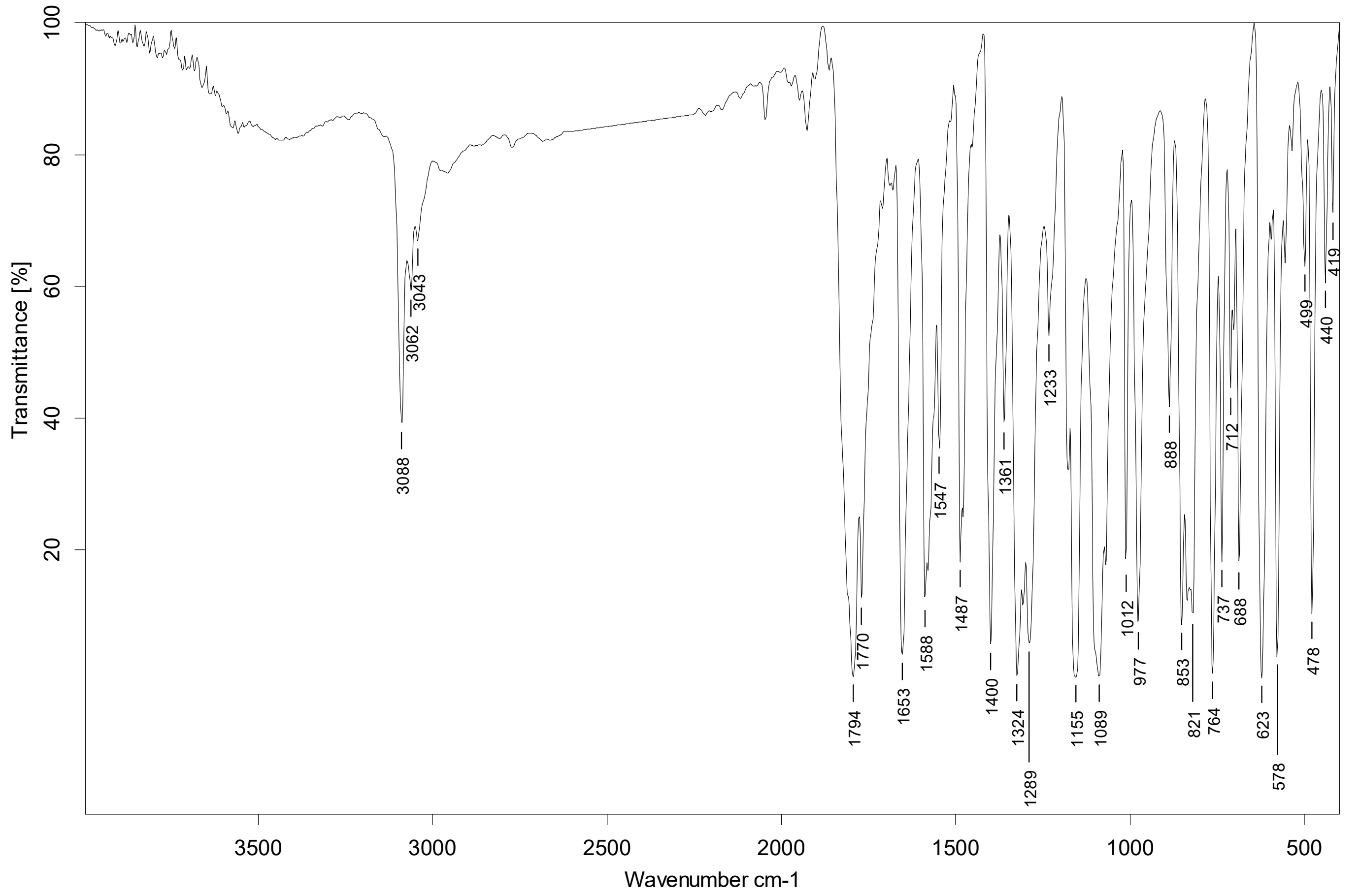

C:\Stefania\Elena_Rosca dr\O (Cl, 4Cl).0	O (Cl, 4Cl)	O (Cl, 4Cl)	30/09/2019

Figure S26. The IR spectrum of oxazol-5(4*H*)-one **2b**.

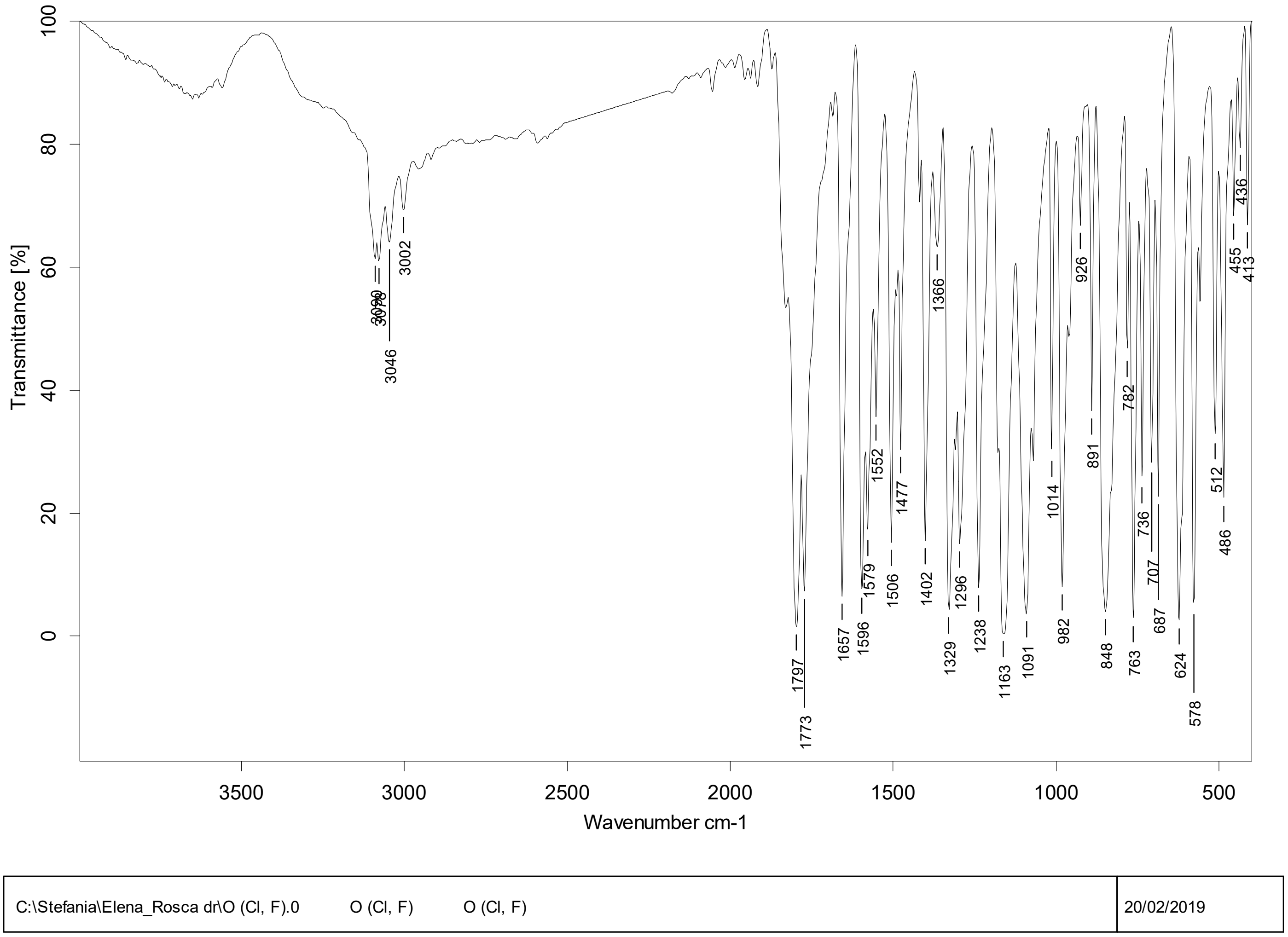

Figure S27. The IR spectrum of oxazol-5(4*H*)-one **2c**.

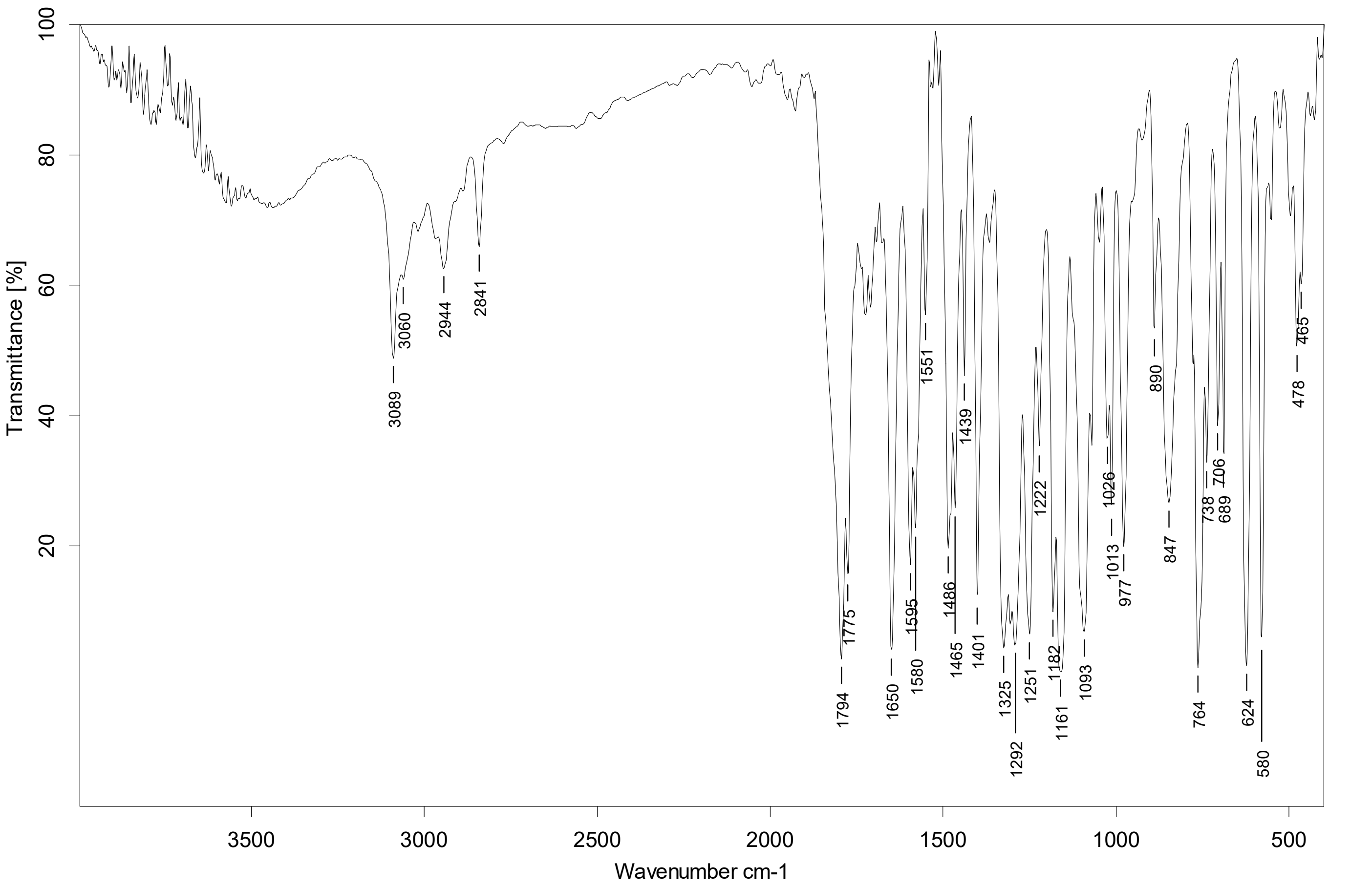

C:\Stefania\Ele_oxazolona_Cl_pmetoxi.0 Ele_oxazolona_Cl_pmetoxi Ele_oxazolona_Cl_pmetoxi	27/06/2018

Figure S28. The IR spectrum of oxazol-5(4*H*)-one **2d**.

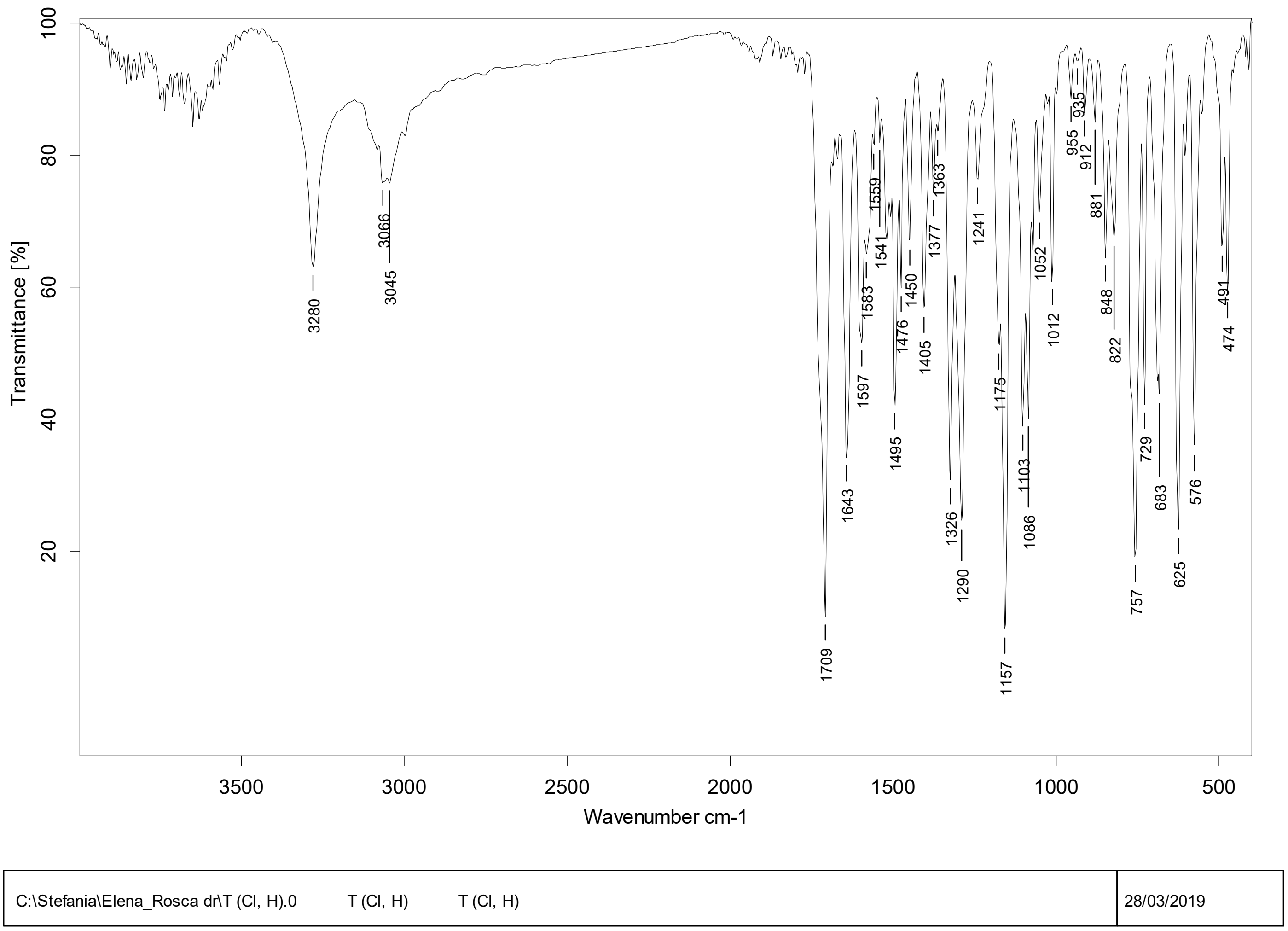

Figure S29. The IR spectrum of triazinone **3a**.

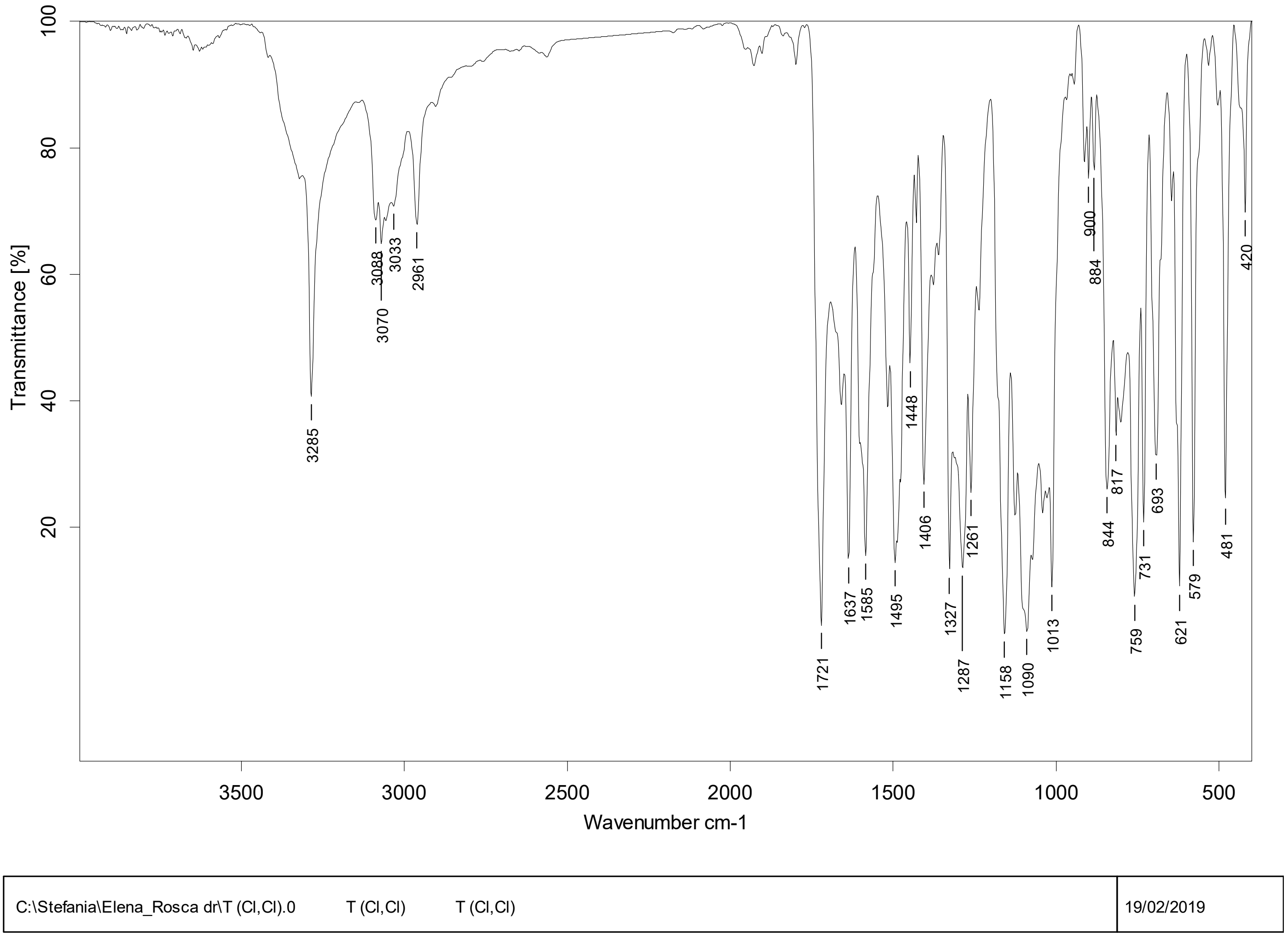

Figure S30. The IR spectrum of triazinone **3b**.

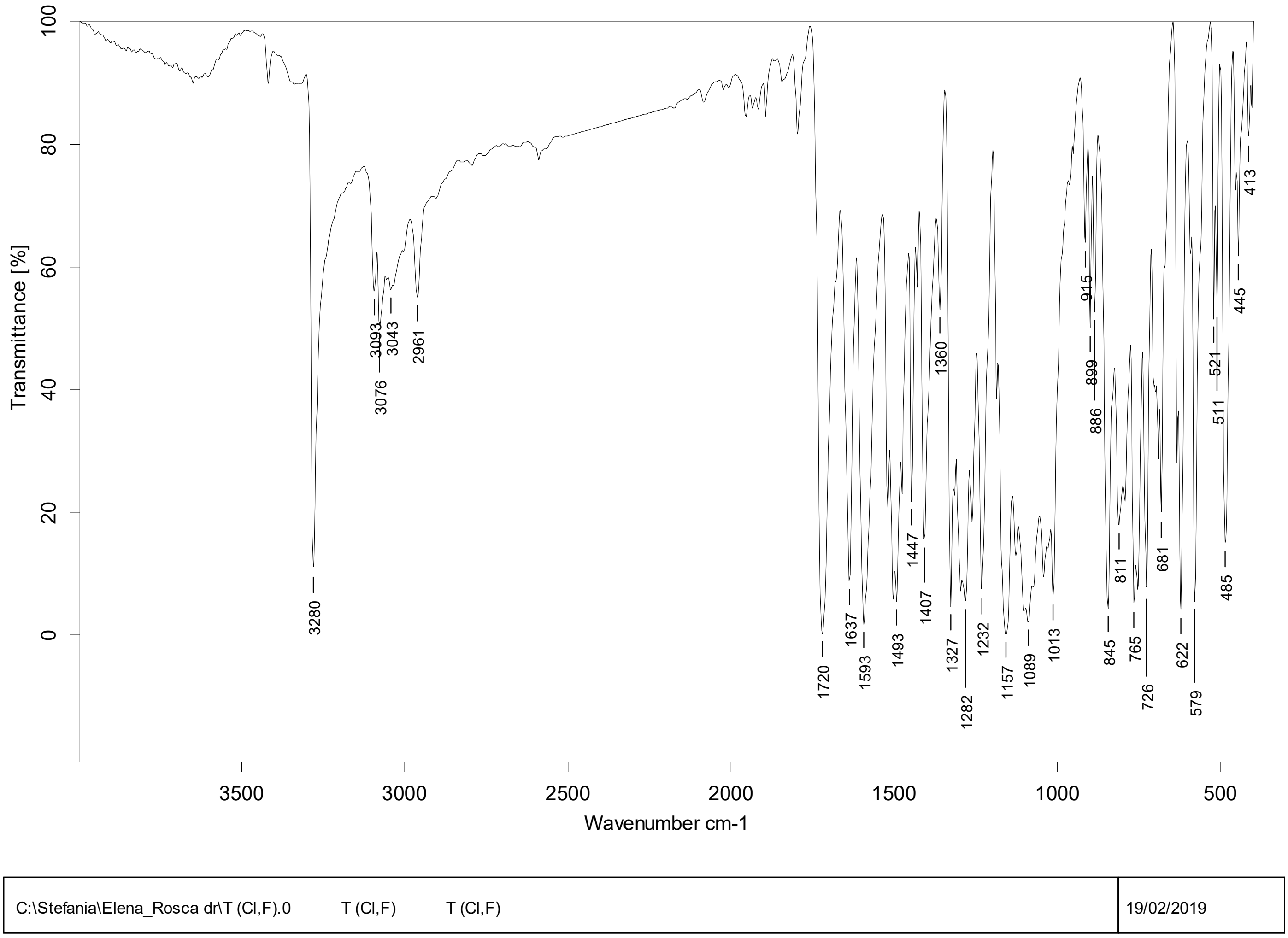

Figure S31. The IR spectrum of triazinone **3c**.

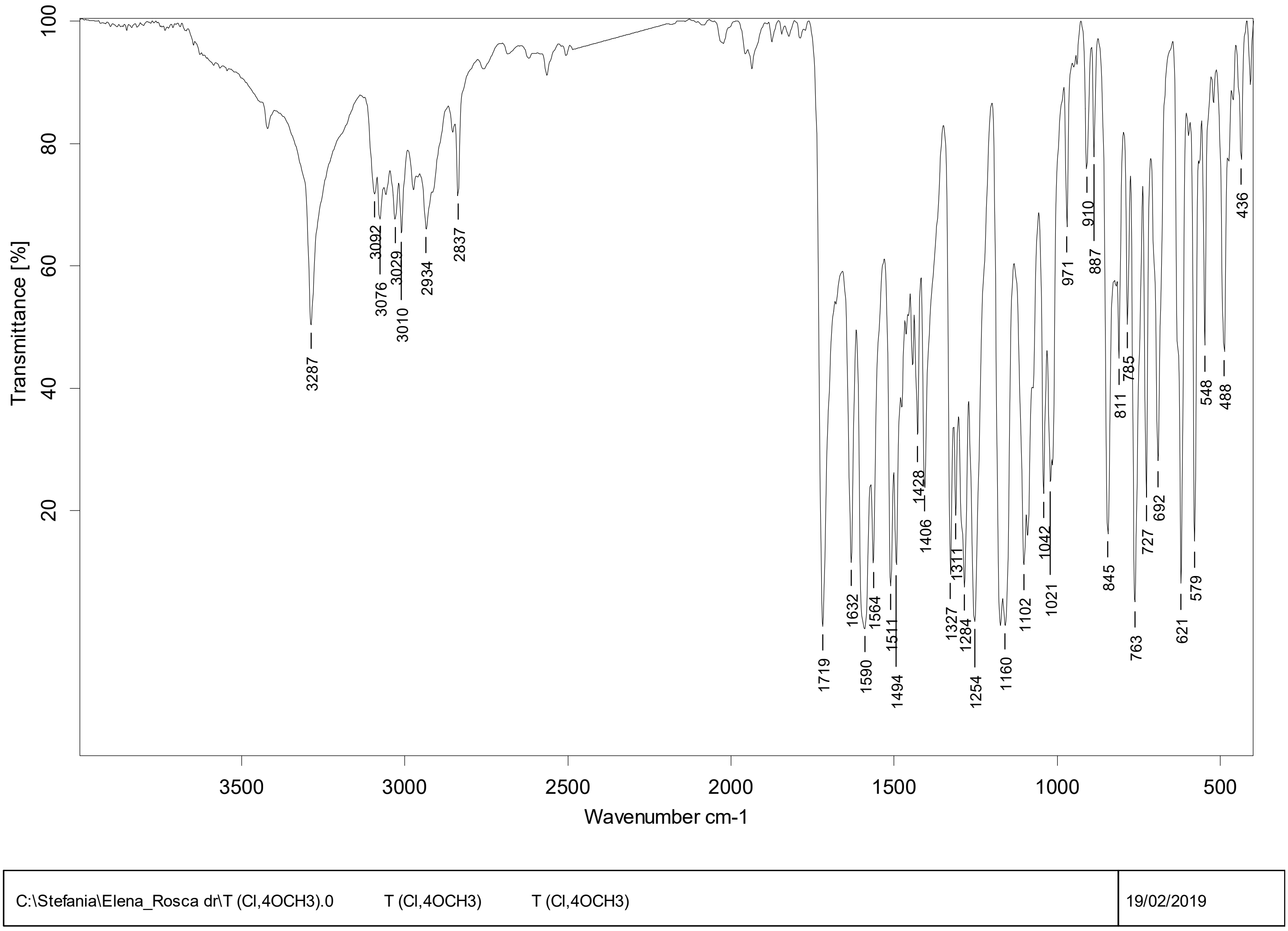

Figure S32. The IR spectrum of triazinone **3d**.

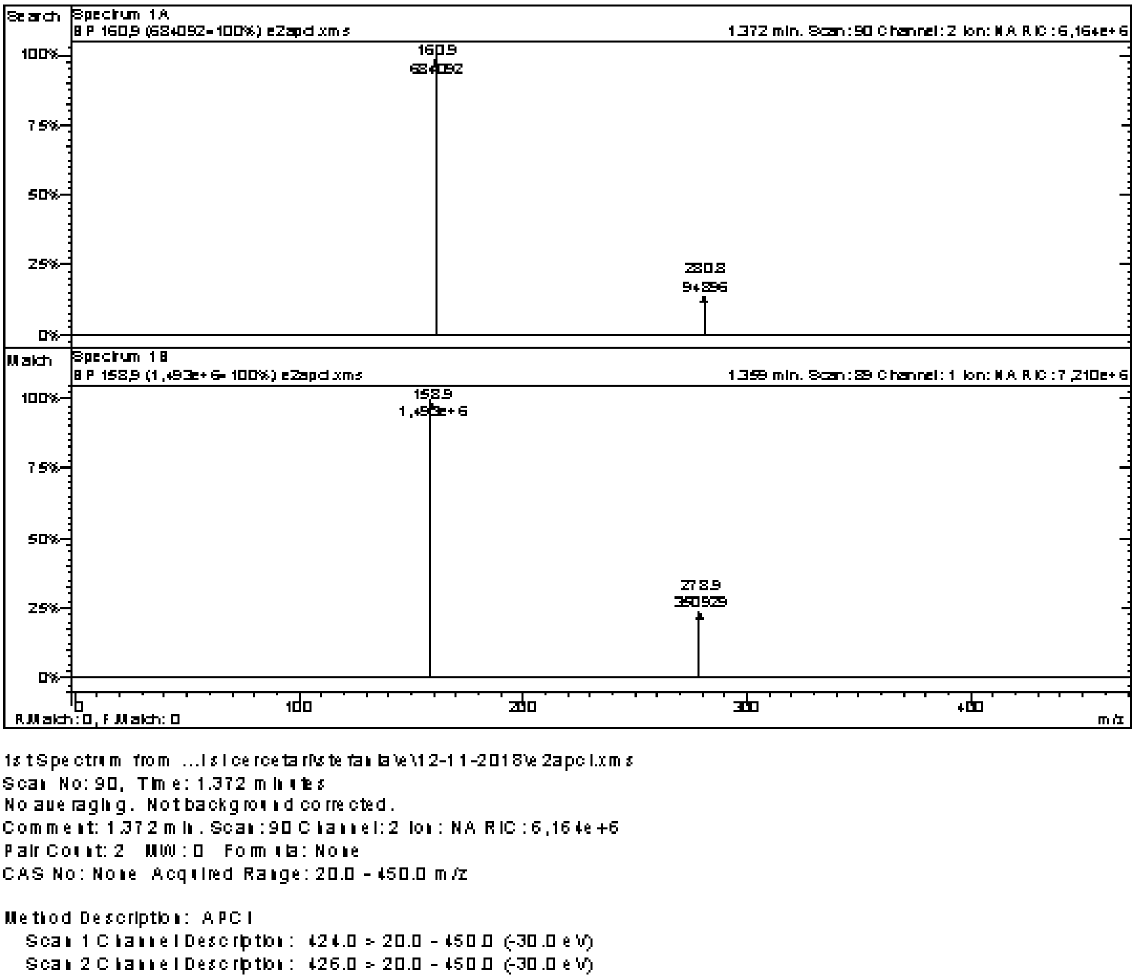

Figure S33. The +APCI-MS spectrum of oxazol-5(4*H*)-one **2a**.

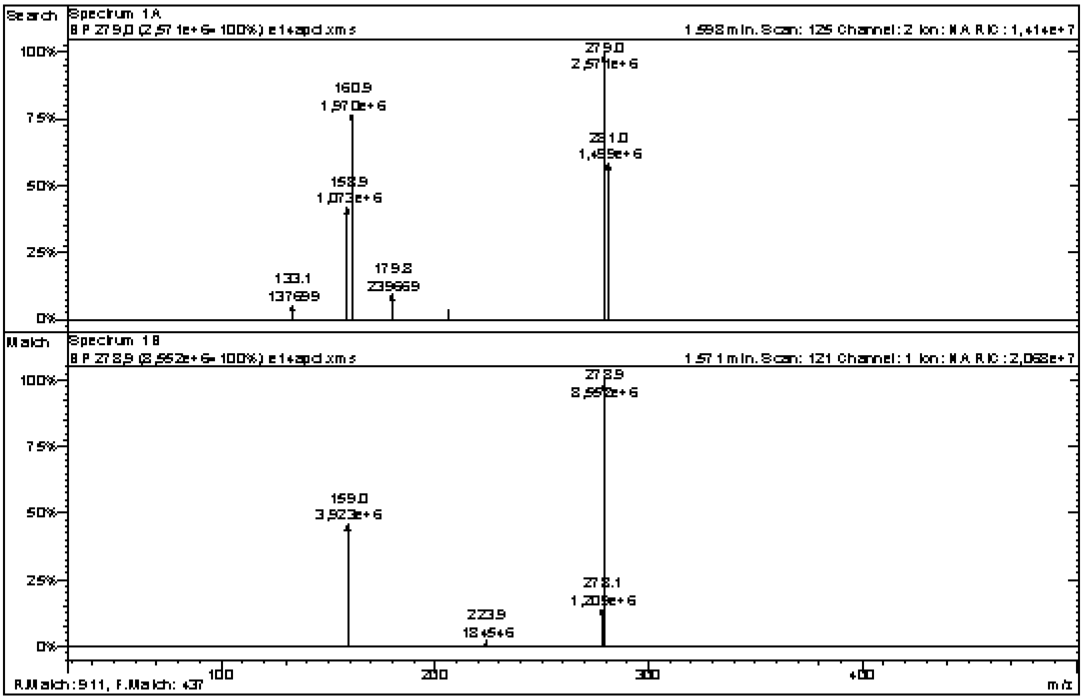

1st Spectrum from ...s\cercetari\stefania\e\12-11-2018\e14apci.xms
Scan No: 125, Time: 1.598 minutes
No averaging. Not background corrected.
Comment: 1.598 min. Scan: 125 Channel: 2 Ion: NA RIC: 1,414e+7
Pair Count: 7 MW: 0 Formula: None
CAS No: None Acquired Range: 50.0 - 480.0 m/z

Method Description: APCI
Scan 1 Channel Description: 458.0 > 50.0 - 480.0 (-20.0 eV)
Scan 2 Channel Description: 460.0 > 50.0 - 480.0 (-20.0 eV)
Scan 3 Channel Description: 462.0 > 50.0 - 480.0 (-20.0 eV)

Figure S34. The +APCI-MS spectrum of oxazol-5(4*H*)-one **2b**.

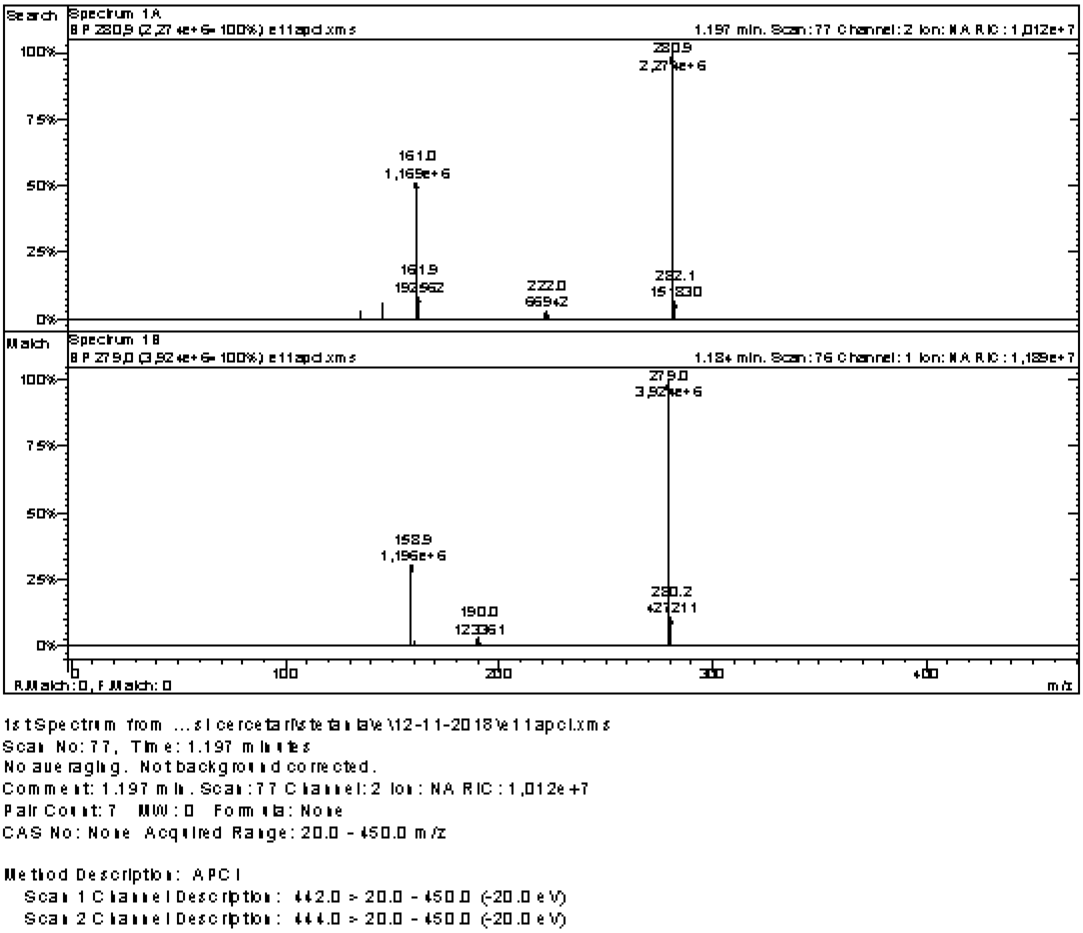

Figure S35. The +APCI-MS spectrum of oxazol-5(4*H*)-one **2c**.

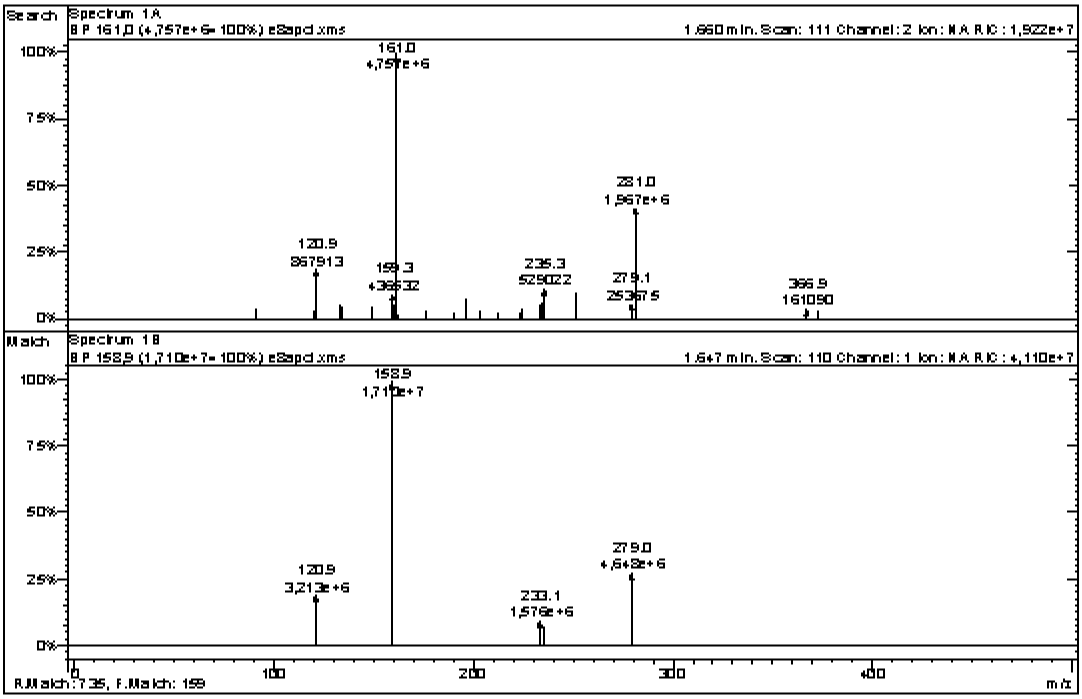

1st Spectrum from ...\sicercetari\stefania\e\12-11-2018\e8apci.xms
Scan No: 111, Time: 1.660 minutes
No averaging. Not background corrected.
Comment: 1.660 min. Scan: 111 Channel: 2 Ion: NA RIC: 1,922e+7
Pair Count: 25 MW: 0 Formula: None
CAS No: None Acquired Range: 20.0 - 480.0 m/z

Method Description: APCI
Scan 1 Channel Description: 454.0 > 20.0 - 480.0 (-30.0 eV)
Scan 2 Channel Description: 456.0 > 20.0 - 480.0 (-30.0 eV)

Figure S36. The +APCI-MS spectrum of oxazol-5(4*H*)-one **2d**.

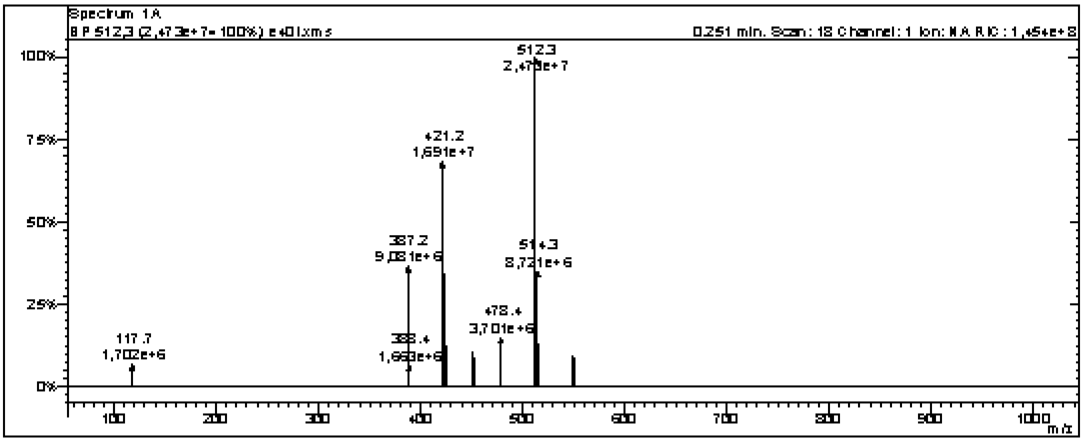

Spectrum from ...d\ils\ce rce tar\Stefania\e\11-07-2019\e40t.xms
Scan No: 18, Time: 0.251 minutes
No averaging. Not background corrected.
Comment: 0.251 min. Scan: 18 Channel: 1 Ion: NA RIC: 1,454e+8
Pair Count: 16 MW: 0 Formula: None
CAS No: None Acquired Range: 100.0 - 1000.0 m/z

Method Description: APCI
Scan 1 Channel Description: 100.0 - 1000.0 >
Scan Information: cp = 0.0 PSI
Precursor Mass Range: 100.0 - 1000.0 m/z

Ion	Int	%BP	Ion	Int	%BP	Ion	Int	%BP
117.7	1,702e+6	6.9	424.3	3,076e+6	12.4	513.3	7,490e+6	30.3
387.2	9,081e+6	36.7	451.3	2,593e+6	10.5	514.3	8,721e+6	35.3
388.4	1,663e+6	6.7	453.3	2,175e+6	8.8	515.4	3,352e+6	13.6
421.2	1,691e+7	68.4	478.4	3,701e+6	15.0	548.4	2,365e+6	9.6
422.3	2,146e+6	8.7	512.3	2,473e+7	100.0	550.3	2,281e+6	9.2
423.1	8,659e+6	35.0						

Figure S37. The –APCI-MS spectrum of triazinone **3a**.

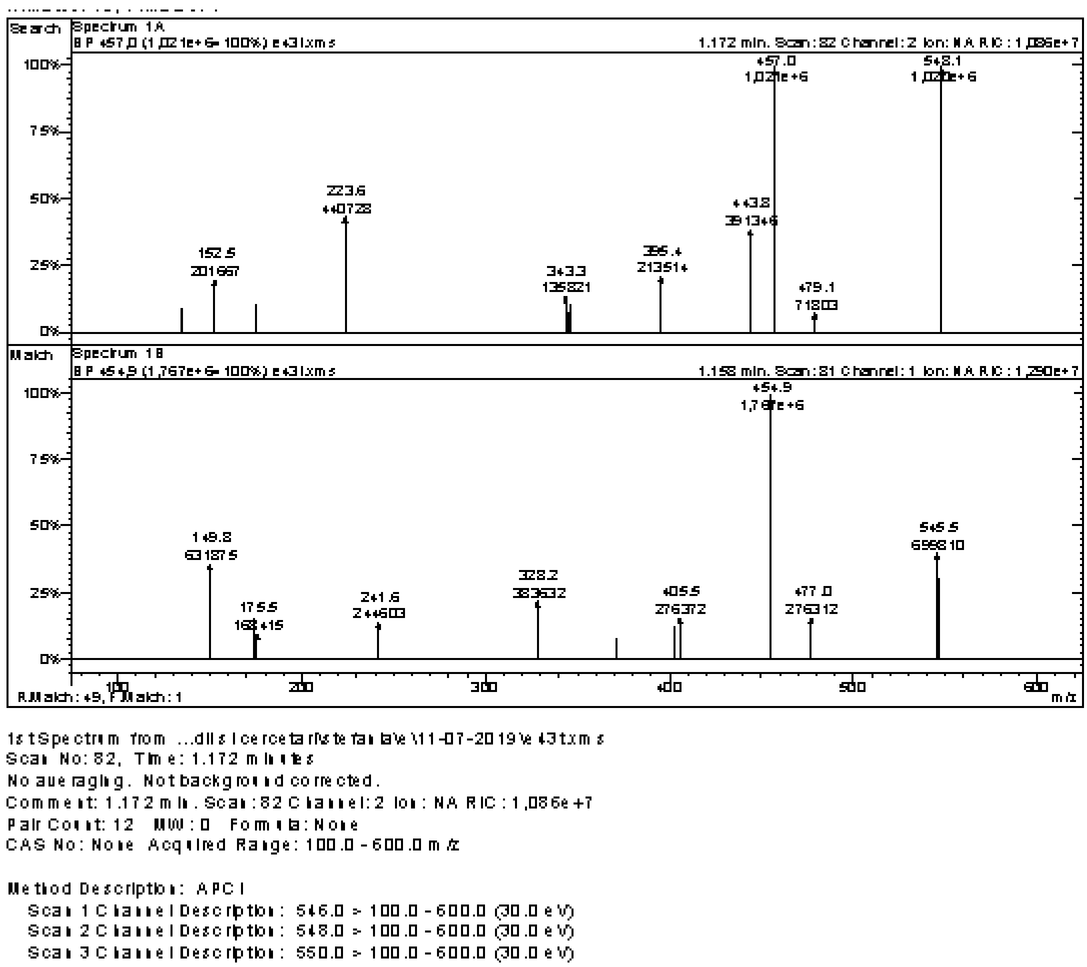

Figure S38. The –APCI-MS spectrum of triazinone **3b**.

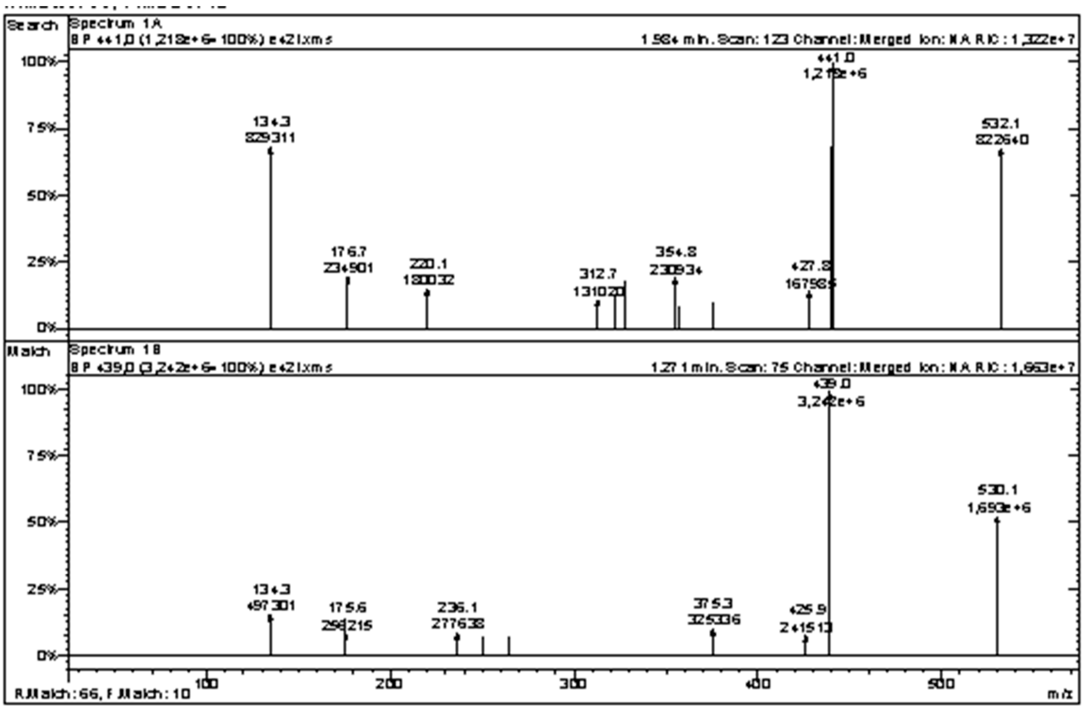

1st Spectrum from ...dllslcerceta rlsteta la\e\11-07-2019\e42txms
Scan No: 123, Time: 1.984 minutes
No averaging. Not background corrected.
Comment: 1.984 min. Scan: 123 Channel: Merged Ion: NA RIC: 1,322e+7
Pair Count: 13 MW: 0 Formula: None
CAS No: None Acquired Range: 50.0 - 550.0 m/z

Figure S39. The –APCI-MS spectrum of triazinone **3c**.

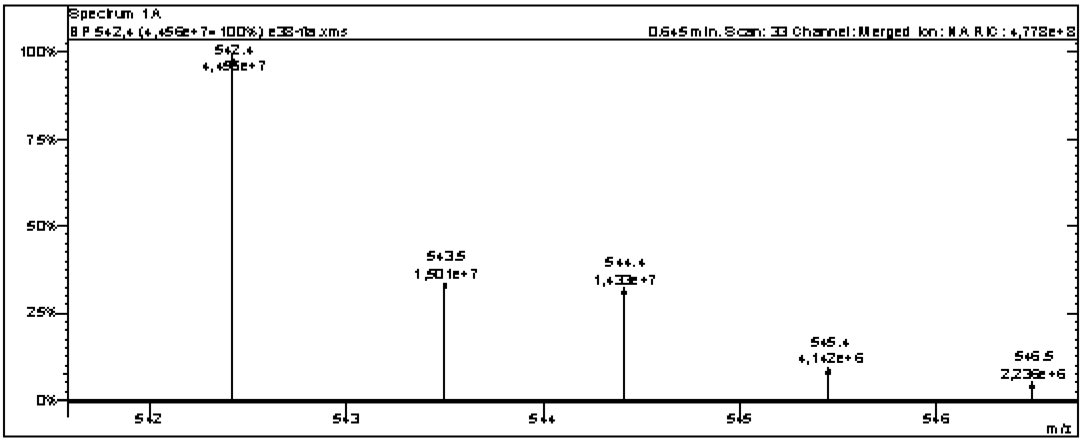

Spectrum from ...slcerceta rlsteta la\e\07-11-2018\e38-fla.xms
Scan No: 33, Time: 0.645 minutes
No averaging. Not background corrected.
Comment: 0.645 min. Scan: 33 Channel: Merged Ion: NA RIC: 4,778e+8
Pair Count: 30 MW: 0 Formula: None
CAS No: None Acquired Range: 150.0 - 1500.0 m/z

Method Description: ESI
Scan 1 Channel Description: 150.0 - 1500.0 >
Scan Information: cp = 0.0 PSI
Precursor Mass Range: 150.0 - 1500.0 m/z

Ion	Int	%BP	Ion	Int	%BP	Ion	Int	%BP
...	...		544.4	1,433e+7	32.2	546.5	2,236e+6	5.0
542.4	4,456e+7	100.0	545.4	4,142e+6	9.3	...	...	
543.5	1,501e+7	33.7						

Figure S40. The –APCI-MS spectrum of triazinone **3d**.
